# Epidemiology of gonorrhea in countries of the Middle East and North Africa: systematic review, meta analyses, and meta regressions

**DOI:** 10.1186/s44263-024-00088-9

**Published:** 2024-08-19

**Authors:** Hiam Chemaitelly, Manale Harfouche, Alex Smolak, Rwedah Ageeb, Yousra A. Mohamoud, Ahmed S. Alaama, Joumana G. Hermez, Laith J. Abu-Raddad

**Affiliations:** 1 Infectious Disease Epidemiology Group, Weill Cornell Medicine-Qatar, Cornell University, Qatar Foundation–Education City, Doha, Qatar; 2 World Health Organization Collaborating Centre for Disease Epidemiology Analytics on HIV/AIDS, Sexually Transmitted Infections, and Viral Hepatitis, Weill Cornell Medicine–Qatar, Cornell University, Qatar Foundation–Education City, Doha, Qatar; 3 Department of Population Health Sciences, Weill Cornell Medicine, Cornell University, New York, NY USA; 4 Department of Communicable Diseases, HIV/Hepatitis/STIs Unit, World Health Organization Regional Office for the Eastern Mediterranean, Cairo, Egypt; 5 Department of Public Health, College of Health Sciences, QU Health, Qatar University, Doha, Qatar; 6 College of Health and Life Sciences, Hamad bin Khalifa University, Doha, Qatar

**Keywords:** *Neisseria gonorrhoeae*, Gonorrhea, Sexually transmitted infection, Prevalence, Infertility, Middle East and North Africa

## Abstract

**Background:**

The epidemiology of *Neisseria gonorrhoeae* (NG) infection in the Middle East and North Africa (MENA) region remains poorly understood, despite the global recognition of its disease burden and the growing concern regarding antimicrobial resistance. This study aimed to systematically review the evidence on NG prevalence in MENA, estimate the pooled mean prevalence across different populations, and explore population-level associations with prevalence as well as sources of between-study heterogeneity.

**Methods:**

The study conducted a systematic review, risk of bias assessment, meta-analyses, and meta-regressions, utilizing both published and unpublished evidence sourced from international, regional, and national databases, in adherence to PRISMA guidelines. Random-effects meta-analyses and meta-regressions were employed to analyze the data.

**Results:**

The study identified 341 NG prevalence measures from 21 countries in MENA. The pooled mean prevalence of current urogenital infection was 1.9% (95% confidence interval (CI) 1.1–2.8%) in the general population, with a higher pooled prevalence in studies with sample sizes < 200 (3.1%; 95% CI 1.5–5.0%) compared to those with sample sizes ≥ 200 (1.1%; 95% CI 0.5–1.9%). Among specific populations, the pooled prevalence was 6.5% (95% CI 4.4–9.0%) in female sex workers, 7.5% (95% CI 2.8–14.0%) in attendees of infertility clinics, 3.0% (95% CI 0.4–7.0%) in women with miscarriage or ectopic pregnancy, 3.9% (95% CI 2.7–5.3%) in symptomatic women, and 41.4% (95% CI 34.9–48.1%) in symptomatic men. For male sex workers and men who have sex with men, the pooled prevalence of current urogenital infection was 1.6% (95% CI 0.4–3.4%), while the prevalence of current anorectal infection was 10.4% (95% CI 4.6–18.0%). Through multivariable meta-regressions, 64% of the prevalence variation was explained, revealing a hierarchical pattern in prevalence by population type and sex, and a prevalence decline at a rate of 1% per year.

**Conclusions:**

NG prevalence in MENA is comparable to the global prevalence, underscoring a neglected and underrecognized disease burden, with social and economic consequences. Persistent transmission of NG among key populations and other populations at risk increases the potential for the emergence of new drug-resistant strains. MENA is far from achieving the World Health Organization’s target of reducing NG incidence by 90% by 2030.

**Supplementary Information:**

The online version contains supplementary material available at 10.1186/s44263-024-00088-9.

## Background

Gonorrhea, caused by the bacterium *Neisseria gonorrhoeae* (NG), is a common sexually transmitted infection (STI) [1–3]. NG infects urogenital, anorectal, or oropharyngeal mucosa [1, 2, 4]. The infection is often asymptomatic, leading to underdiagnosis and undertreatment, particularly in women [1, 2, 4]. Untreated NG can result in complications such as vaginal discharge, bleeding, urethritis, cervicitis, pelvic inflammatory disease, ectopic pregnancy, and infertility [1, 2, 5, 6]. The World Health Organization (WHO) estimated 86.9 million new infections worldwide in 2016 [7], with recent data showing increasing incidence in specific population groups across several countries [8–10].

The global health concern associated with gonorrhea has escalated due to widespread gonococcal antimicrobial resistance (AMR) and the emergence of extensively drug-resistant NG strains [11–14]. This includes strains resistant to extended-spectrum cephalosporins, which are currently the last line of defense against this infection [2, 11, 12, 15]. These treatment challenges have further complicated gonorrhea control efforts. Recognizing the urgency, the WHO declared gonococcal AMR a global high priority [16] and launched a global action plan to control NG transmission [17].

The WHO’s “Global Health Sector Strategy on STIs” addresses STIs as a critical public health concern [18]. It aims to reduce NG incidence worldwide by 90% by 2030 through evidence-based interventions and improved access to quality services [18]. As stated in the strategy, the first strategic direction emphasizes “the need to understand the STI epidemic as a basis for advocacy, political commitment, national planning, resource mobilization, and allocation, implementation, and programme improvement” [19]. Preventing and controlling gonorrhea spread and gonococcal AMR is a global health priority, requiring a comprehensive understanding of its epidemiology. The potential availability of vaccination as an intervention [20–23] also emphasizes the importance of understanding NG epidemiology across various population groups. This knowledge is essential in guiding the targeted deployment of the vaccine once it becomes available in the coming years.

Despite the urgency, the Middle East and North Africa (MENA) region, which accounts for 10% of the world’s population [24], faces significant challenges with weak STI surveillance systems, scarce sexual health programs, and a lack of understanding of NG infection rates and disease burden [25–31]. In light of this, this study aims to analyze and quantify the epidemiology of NG in MENA by (1) systematically reviewing and synthesizing all available published and unpublished records on NG prevalence, (2) estimating the pooled mean prevalence among different populations, and (3) identifying population-level associations with prevalence and sources of between-study heterogeneity.

Both overall (i.e., encompassing the entire sample) and stratified measures were extracted from the relevant studies included in this review. The objective was to investigate the natural heterogeneity in NG epidemiology by stratifying the measures based on epidemiological factors that influence the infection’s epidemiology [7, 32–35]. Meta-regression analyses were conducted on these stratified measures to evaluate the effects of these epidemiological factors on NG prevalence, explore temporal trends, and identify sources of between-study heterogeneity. This analytical approach enables the generation of insights into the infection's epidemiology by explaining the underlying variations in available measures [36].

## Methods

### Data sources and search strategy

A systematic review of epidemiological evidence on NG prevalence in MENA was conducted, following the Cochrane Collaboration's methods for guidance [37]. The findings were reported in accordance with the Preferred Reporting Items for Systematic Reviews and Meta-analyses (PRISMA) guidelines [38, 39], utilizing the checklist provided in Additional file 1: Table S1. The literature search was comprehensive and encompassed international databases (PubMed and Embase), regional databases (WHO Index Medicus for the Eastern Mediterranean Region, the Iraqi Academic Scientific Journals’ database, the Scientific Information Database of Iran, and the PakMediNet of Pakistan), as well as country-level and international organizations’ reports and records accessible through the MENA HIV/AIDS Epidemiology Synthesis Project archive [26, 29]. The search covered records up to February 28, 2023.

The search criteria utilized in this study were deliberately broad, aiming to cast a wide and inclusive net. Index terms were expanded to cover all subheadings, and free text terms were incorporated (Additional file 1: Table S2). No restrictions were applied regarding language or year. The list of countries included in MENA can be found in Additional file 1: Box S1. The definition of MENA follows earlier conventions adopted in infectious disease research [28, 29, 40–42], and is based on the definitions provided by the WHO’s Regional Office for the Eastern Mediterranean and the Joint United Nations Programme on HIV/AIDS.

### Study selection process and inclusion and exclusion criteria

The search results were imported into the reference manager Endnote (Thomson Reuters, USA) for deduplication and screening purposes. Initially, titles and abstracts were screened to identify relevant and potentially relevant reports. Full texts of these reports were then retrieved and screened for relevancy. Relevant reports included those presenting primary data on NG prevalence in any of the 23 MENA countries (Additional file 1: Box S1), based on laboratory testing methods such as nucleic acid amplification test (NAAT)/polymerase chain reaction (PCR), culture, wet mount, and gram stain, irrespective of the prevalence values measured. Excluded reports encompassed NG prevalence studies relying on self-reporting, studies involving fewer than 10 individuals, and investigations focusing on testing specimens of the upper genital tracts. Case reports, case series, reviews, editorials, and reports concerning NG in foreign military personnel stationed in the region were also excluded. Bibliography screening of relevant articles and literature reviews was also conducted manually to identify any additional eligible reports.

In this article, the term “record” refers to a document such as an article or public health report that includes prevalence measures for one or more populations. On the other hand, the term “study” refers to a specific prevalence measure conducted in a particular population. Duplicate findings from studies were included only once, prioritizing the more detailed record.

### Data extraction and data synthesis

HC, MH, AS, RA and YM conducted the extraction and double extraction of overall outcome measures and their stratifications from the relevant records. Stratified data extraction was performed if the sample size in each stratum was ≥ 10. The extraction process followed a pre-piloted list, which can be found in Additional file 1: Box S2. The stratified data were extracted based on a predetermined hierarchy informed by epidemiological relevance and prior knowledge of HIV/STI epidemiology [6, 35, 43, 44]. This hierarchy included factors such as anatomical site/mode of transmission, population type, sex, year of data collection, and age group.

Population type was classified according to risk of exposure to NG (Table 1), based on the characteristics of the population rather than the recruitment study site. For example, pregnant women attending family planning clinics (a healthcare-seeking population) were considered part of the general population because they were seeking routine care unrelated to NG infection. Any population attending a clinical setting with indications, symptoms, or exposures potentially related to NG infection or any other STIs was not considered part of the general population.

**Table 1:** Definitions of population type classifications

1. General populations (populations at low risk): these include populations at low risk of exposure to gonorrhea such as antenatal clinic attendees, blood donors, and pregnant women, among others.
2. Intermediate-risk populations: these include populations who presumably have frequent sexual contact with populations engaging in high sexual risk behavior, and have therefore a higher risk of exposure to gonorrhea than the general population. These comprise prisoners, people who inject drugs, truck drivers, and migrant workers, among others.
3. Female sex workers: these include women who are engaged in sex work, that is the exchange of sex for money (sex work as a profession).
4. Male sex workers and men who have sex with men: these include men who engage in same-sex sexual activities, specifically anal sex, and men who are engaged in providing sexual services in return for payment.
5. Symptomatic women: these include women with clinical manifestations related to gonorrhea or suspected of having gonorrhea, such as those with vaginal discharge.
6. Symptomatic men: these include men with clinical manifestations related to gonorrhea or suspected of having gonorrhea, such as those with urethral discharge.
7. Symptomatic mixed sexes: these include populations with undetermined sex with clinical manifestations related to gonorrhea or suspected of having gonorrhea, such as those with vaginal discharge or urethral discharge.
8. Infertility clinic attendees: these were included in a separate category given the uncertainty around their risk of exposure to gonorrhea, and the possible biological link between gonorrhea and infertility.
9. Women with miscarriage or ectopic pregnancy: these were included in a separate category given the uncertainty around their risk of exposure to gonorrhea, and the possible biological link between gonorrhea and miscarriage or ectopic pregnancy.
10. STI clinic attendees: these include patients attending STI clinics.
11. Individuals living with HIV and individuals in HIV-discordant couples: these include populations who are living with HIV or are in a spousal relationship with an individual living with HIV.
12. Patients with confirmed/suspected STIs and related infections: these include populations who are diagnosed with STIs or suspected to have concomitant STIs or other related infections.
13. Other populations: these include populations not satisfying the above definitions or populations with an undetermined risk of acquiring gonorrhea.

*Abbreviations*: *STI* Sexually transmitted infection, *HIV* Human immunodeficiency virus

For studies reporting an overall measure for both men and women, sex classification was determined based on the predominant sex in the sample, with a threshold of over 60%. Studies reporting NG prevalence among children below 15 years old were reported but not included in the subsequent analyses.

Studies that utilized the same assay to test different biological specimens within a specific population were included only once. The selection followed a sequential order, prioritizing NG detection in endocervical swabs for women, followed by vaginal swabs and urine samples. For men, the priority order was urethral swabs, followed by urine and semen samples.

On the other hand, studies that employed different assays on the same biological specimens were extracted separately. This approach aimed to evaluate the assay effect on the heterogeneity of NG prevalence and to generate adjustment factors [45–47] for estimating NG prevalence in future mathematical modeling studies that investigate NG infection and its disease burden.

### Precision and risk of *bias* assessments

All included studies were assessed for precision and risk of bias (ROB). The precision of each study was classified as either “low” or “high” based on the sample size (< 200 participants versus ≥ 200 participants). Informed by the Cochrane Collaboration approach [37], each study was categorized as having either “low” or “high” ROB in two quality domains: sampling methodology (probability-based versus non-probability-based) and response rate (≥ 80% response rate versus < 80%). If a study had missing information for a specific domain, it was classified as having “unclear” ROB for that domain. These data were also included in meta-regression analyses to examine their effect on the observed NG prevalence, following the methodology used in our previous studies [35, 41–44, 48].

### *Meta*-analyses

Dersimonian-Laird random-effects models were employed to conduct meta-analyses [49] for NG prevalence, applying the Freeman-Tukey double arcsine transformation to stabilize the variance [50, 51]. Before applying this transformation, its appropriateness for the analysis was evaluated by examining the distribution of study sample sizes and effect sizes to ensure that these distributions were not severely skewed, which could potentially introduce bias [52]. Pooled mean prevalence estimates, along with their corresponding 95% confidence intervals (CI), were calculated for each population type based on the anatomical site and assay type, provided that the stratum contained ≥ 3 measures. Pooled mean prevalence was also estimated by MENA country and by study precision for urogenital NG prevalence among general populations, considering the available number of studies for these populations and the epidemiological relevance. Forest plots were generated to visualize the results.

Heterogeneity was assessed using Cochran’s *Q* statistic (*p* value < 0.1) to confirm the existence of heterogeneity across studies, *I*^2^ to quantify the magnitude of between-study variation that is due to true differences in prevalence across studies rather than chance, and prediction interval to estimate the distribution of true prevalence around the pooled mean [49, 53]. Meta-analyses were conducted using the statistical computing and data visualization program R version 4.1.3 [54], utilizing the “meta” package [55].

Considering the heterogeneity among the prevalence measures, the pooled means should be interpreted as average summary measures [36, 44], not definitive estimates of prevalence. The meta-regression analyses described below investigated and explained the sources of variation in prevalence measures, considering both epidemiological factors and study methods.

### *Meta*-regressions

Univariable and multivariable random-effects meta-regression analyses were conducted on log-transformed prevalence measures to explore the factors influencing NG prevalence and explain the heterogeneity observed between studies in MENA. This approach aimed to identify potential predictors associated with higher NG prevalence within the region. The predictors were selected based on their epidemiological relevance and prior knowledge of HIV/STI epidemiology [36, 43, 44, 48], as described in Additional file 1: Box S3. Variables with a *p* value ≤ 0.10 in the univariable analysis were included in the multivariable analysis. Associations with a *p* value ≤ 0.05 in the multivariable analysis were deemed statistically significant.

Missing values for the year of data collection were imputed using the year of publication data adjusted by the median difference between the year of publication and the year of data collection for studies with complete information. Meta-regressions were conducted using the statistical analysis software Stata/SE version 16 [56], utilizing the “metareg” package [57].

## Results

### Search results and scope of evidence

**Fig. 1:**
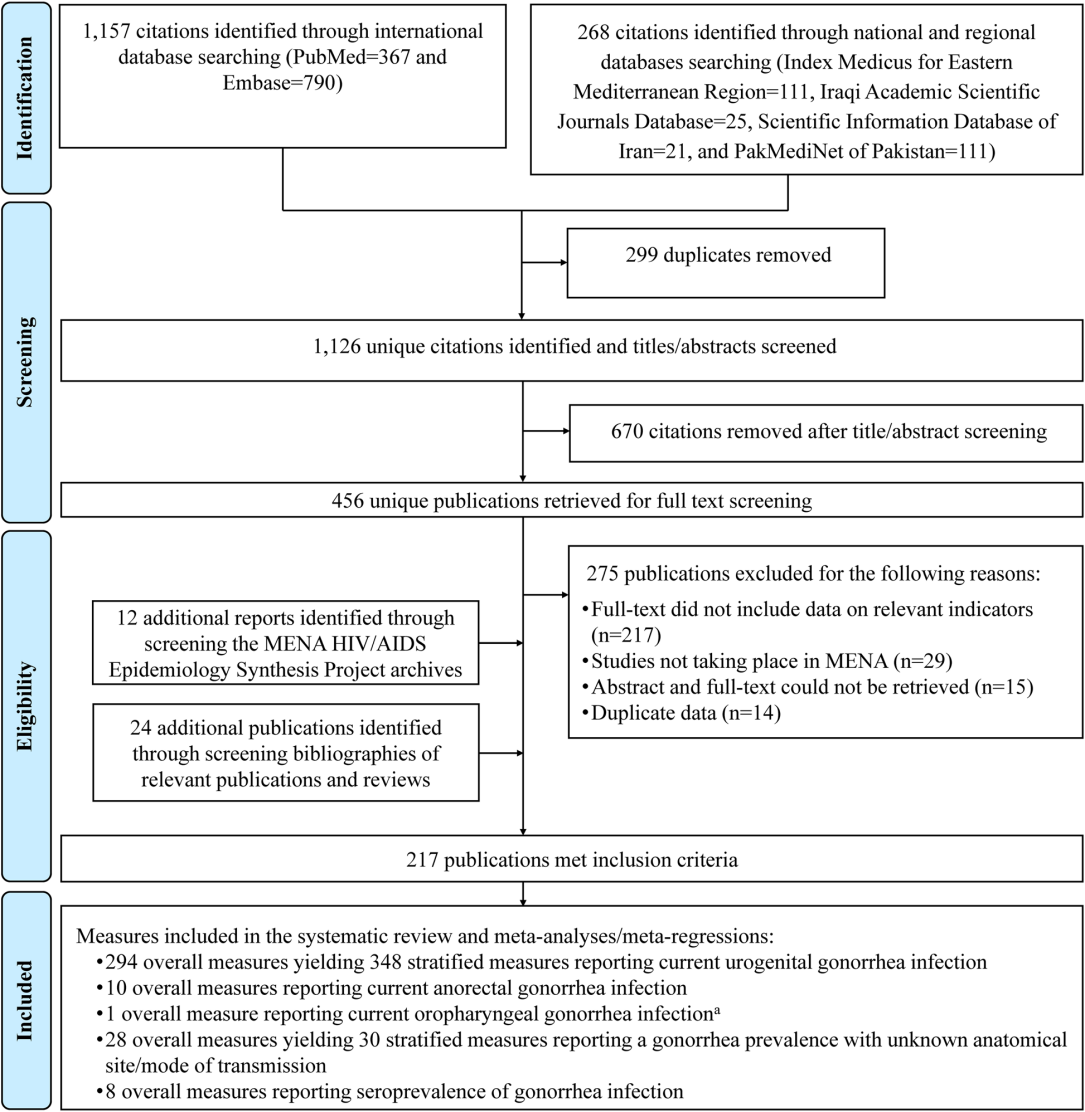
Study selection flowchart for assessing *Neisseria gonorrhoeae* prevalence in the Middle East and North Africa, compliant with PRISMA guidelines *Abbreviations:* *AIDS* Acquired immunodeficiency syndrome, *HIV* Human immunodeficiency virus, *MENA* Middle East and North Africa, *PRISMA* Preferred Reporting Items for Systematic Reviews and Meta-analyses ^a^The publication reporting this measure has insufficient clarity in its methods, making it difficult to determine the accuracy of the reported prevalence

The PRISMA study selection process is illustrated in Fig. 1. The initial search conducted in international databases (PubMed 367 and Embase 790) identified 1157 records. Regional databases yielded 268 records, with contributions from the Index Medicus for Eastern Mediterranean Region (111 records), Iraqi Academic Scientific Journals Database (25 records), Scientific Information Database of Iran (21 records), and PakMediNet of Pakistan (111 records).

After removing duplicate records and conducting title and abstract screening, as well as full-text screening, 181 records were deemed relevant. By screening the MENA HIV/AIDS Epidemiology Synthesis Project archive, 12 more relevant records were identified [58–69]. By screening bibliographies of relevant articles and reviews, an additional 24 relevant records were found [70–93]. Overall, a total of 217 records met the inclusion criteria for the study.

Among the records that met the inclusion criteria, the extracted NG prevalence measures included 294 overall urogenital measures (348 measures when stratified by different factors), 10 overall anorectal measures, 1 overall oropharyngeal measure, 28 overall measures of unspecified anatomical sites (30 stratified measures), and 8 overall serological measures.

The evidence covered data from 21 out of the 23 MENA countries. The largest volume of data was obtained from Iran, with 58 reports including 123 prevalence measures among 32,988 individuals. Iraq followed with 37 reports including 76 prevalence measures among 8379 individuals.

### Gonorrhea prevalence overview

The overall NG prevalence measures in MENA are summarized in Additional file 1: Table S3 and Table S4, categorized by anatomical site and population type. The extracted measures span a wide timeframe, with the earliest measure published in 1977. Notably, 24.9% of the measures (85 measures) were published in 2015 and onwards.

Among 294 studies reporting urogenital NG prevalence measures, 13.3% reported zero prevalence. For the 10 anorectal NG prevalence measures, one study reported zero prevalence. Only one study reported on oropharyngeal NG prevalence, which was found to be 99.1%, raising concerns about the validity of the laboratory methods used [94]. The study had insufficient clarity in its methods making it difficult to determine the accuracy of the reported prevalence.

Tables 2, 3 and 4 summarize the ranges and medians of stratified NG prevalence measures by population type, anatomical site, and assay type. Additional file 1: Table S5 complements this information by reporting prevalence measures by MENA country and study precision (< 200 participants versus ≥ 200 participants).

**Table 2:** Results of meta-analyses on studies reporting *Neisseria gonorrhoeae* prevalence in general populations, intermediate-risk populations, infertility clinic attendees, women with miscarriage or ectopic pregnancy, and other populations in the Middle East and North Africa

Population type^a^		Stratified prevalence measures	Sample size	NG prevalence (%)		Pooled mean NG prevalence	Heterogeneity measures		
		Total n	Total *N*	Range	Median	Mean (%) (95% CI)	*Q*^b^ *(p* value)	*I*^2c^ (%) (95% CI)	Prediction interval^d^ (%)
General populations									
Current urogenital infection	NAAT/PCR	39	25,592	0.0–30.0	1.0	1.5 (0.7–2.6)	779.8 (*p* < 0.001)	95.4 (94.1–96.0)	0.0–11.3
	Culture	26	8567	0.0–20.0	0.8	1.0 (0.3–1.9)	121.6 (*p* < 0.001)	79.4 (70.5–85.7)	0.0–7.3
	Gram stain	16	6266	0.0–40.0	3.4	5.7 (1.6–11.6)	231.0 (*p* < 0.001)	93.5 (90.9–95.3)	0.0–40.4
	**Overall**	**81**	**40,425**	**0.0–40.0**	**1.0**	**1.9 (1.1–2.8)**	**1,161.5 (*p* < 0.001)**	**93.1 (92.0**–**94.1)**	**0.0**–**14.5**
Unspecified/mixed anatomical site	NAAT/PCR	3	1415	0.7–1.2	0.9	0.8 (0.4–1.4)	1.1 (*p* = 0.576)	0.0 (0.0–89.6)	0.0–6.8
	Culture	1	150	–	–	2.0 (0.4–5.7)	–	–	–
	Other/unclear assay^e^	6	15,028	0.4–5.0	1.0	1.3 (0.2–2.9)	20.9 (*p* < 0.001)	76.1 (46.3–89.3)	0.0–8.3
	**Overall**	**10**	**16,593**	**0.4–5.0**	**1.0**	**1.0 (0.4–1.8)**	**30.8 (*p *< 0.001)**	**70.7 (44.1**–**84.7)**	**0.0**–**3.8**
Sera	Blood tested for IgG antibodies	3	197	0.0–2.0	0.0	0.9 (0.0–3.2)	0.81 (*p* = 0.667)	0.0 (0.0–89.6)	0.0–30.4
Intermediate risk populations									
Current urogenital infection	NAAT/PCR	10	3151	0.0–3.5	1.0	0.9 (0.4–1.7)	32.2 (*p* < 0.001)	72.1 (47.0–85.3)	0.0–4.3
	Culture	3	877	0.0–0.0	0.0	0.0 (0.0–0.2)	0.11 (*p* = 0.948)	0.0 (0.0–89.6)	0.0–5.6
	Other/unclear assay^e^	1	199	–	–	4.5 (2.1–8.4)	–	–	–
	**Overall**	**14**	**4227**	**0.0–4.5**	**0.9**	**0.8 (0.2–1.5)**	**61.3 (*p* < 0.001)**	**78.8 (65.0**–**87.2)**	**0.0**–**4.8**
Unspecified/mixed anatomical site	NAAT/PCR	1	400	–	–	0.5 (0.1–1.8)	–	–	–
	**Overall**	**1**	**400**	**–**	**–**	**0.5 (0.1–1.8)**	–	–	–
Infertility clinic attendees									
Current urogenital infection	NAAT/PCR	15	1740	0.0–70.0	2.0	6.0 (0.7–15.3)	255.5 (*p* < 0.001)	94.5 (92.4–96.0)	0.0–61.4
	Culture	16	1768	0.0–75.0	4.1	9.2 (2.3–19.3)	211.3 (*p* < 0.001)	92.9 (90.0–95.0)	0.0–65.3
	**Overall**	**31**	**3508**	**0.0–75.0**	**2.3**	**7.5 (2.8–14.0)**	**467.8 (*p* < 0.001)**	**93.6 (91.9**–**94.9)**	**0.0**–**58.6**
Unspecified/mixed anatomical site	Other/unclear assay^e^	1	373	–	–	14.2 (10.8–18.2)	–	–	–
	**Overall**	**1**	**373**	**–**	**–**	**14.2 (10.8–18.2)**	–	–	–
Sera	Blood tested for antibodies	1	79	–	–	2.5 (0.3–8.8)	–	–	–
Women with miscarriage or ectopic pregnancy									
Current urogenital infection	NAAT/PCR	4	339	0.0–7.6	3.4	2.8 (0.1–8.0)	14.3 (*p* = 0.002)	79.1 (44.1–92.2)	0.0–38.4
	Culture	1	81	–	–	3.7 (0.8–10.4)	–	–	–
	**Overall**	**5**	**420**	**0.0–7.6**	**3.7**	**3.0 (0.4–7.0)**	**14.4 (*p* = 0.006)**	**72.3 (30.3**–**89.0)**	**0.0**–**21.6**
Sera	Blood tested for antibodies	2	90	0.0–13.3	6.7	4.4 (1.2–11.0)^f^	–	–	–
Other populations^g^									
Current urogenital infection	Culture	1	72	–	–	1.4 (0.0–7.5)	–	–	–
	Gram stain	1	200	–	–	2.0 (0.5–5.0)	–	–	–
	Other/unclear assay^e^	2	4030	2.7–2.7	2.7	2.7 (2.2–3.2)^f^	–	–	–
	**Overall**	**4**	**4302**	**1.4–2.8**	**2.2**	**2.2 (1.7–2.7)**	**0.5 (*p* = 0.915)**	**0.0 (0.0**–**84.7)**	**1.2**–**3.3**
Sera	Blood tested for antibodies	2	258	2.3–11.1	6.7	3.5 (1.6–6.5)^f^	–	–	–

*Abbreviations*:* CI* Confidence interval, *NAAT* Nucleic acid amplification test, *NG* *Neisseria gonorrhoeae*, *PCR* Polymerase chain reaction

A minimum of three studies were required to conduct a meta-analysis

Bolded numbers represent overall pooled estimates

^a^ Population type classification can be found in Table 1

^b^*Q*: The Cochran’s *Q* statistic is a measure assessing the existence of heterogeneity in pooled outcome measures, here NG prevalence

^c^*I*^*2*^: A measure that assesses the magnitude of between-study variation that is due to actual differences in NG prevalence across studies rather than chance

^d^Prediction interval: A measure that estimates the distribution (95% interval) of true NG prevalence around the estimated mean

^e^Other/unclear assay include enzyme immunoassay, indirect hemagglutination, or mixed/unclear testing technique

^f^Two prevalence measures are not sufficient to conduct a random-effects meta-analysis. The pooled measure was calculated as the arithmetic mean of the two measures and their 95% confidence intervals

^g^Other populations include populations with an undetermined risk of acquiring NG infection such as victims of sexual assault and mixed populations, among others

**Table 3:** Results of meta-analyses on studies reporting *Neisseria gonorrhoeae* prevalence in higher-risk populations, STI clinic attendees, and individuals living with HIV and individuals in HIV-discordant couples in the Middle East and North Africa

Population type^a^		Stratified prevalence measures	Sample size	NG prevalence (%)		Pooled mean NG prevalence	Heterogeneity measures		
		Total *n*	Total *N*	Range	Median	Mean (%) (95% CI)	*Q*^b^ *(p* value)	*I*^2c^ (%) (95% CI)	Prediction interval^d^ (%)
Female sex workers									
Current urogenital infection	NAAT/PCR	14	5976	0.8–12.3	8.4	6.0 (3.7–8.9)	243.2 (*p* < 0.001)	94.7 (92.5–96.2)	0.0–20.4
	Culture	2	466	1.4–3.7	2.6	2.4 (1.2–4.2)^e^	–	–	–
	Gram stain	6	921	0.0–16.6	11.3	11.4 (8.8–14.3)	7.3 (*p* = 0.202)	31.1 (0.0–72.0)	6.0–18.1
	**Overall**	**22**	**7363**	**0.0–16.6**	**8.4**	**6.5 (4.4–9.0)**	**327.7 (*p* < 0.001)**	**93.6 (91.5–95.1)**	**0.0–20.9**
Unspecified/mixed anatomical site	Culture	1	89	–	–	11.2 (5.5–19.7)	–	–	–
	**Overall**	**1**	**89**	**–**	**–**	**11.2 (5.5–19.7)**	**–**	**–**	**–**
Male sex workers and men who have sex with men^f^									
Current urogenital infection	NAAT/PCR	12	2680	0.0–8.8	2.2	1.6 (0.4–3.4)	81.8 (*p* < 0.001)	86.5 (78.3–91.7)	0.0–11.0
	**Overall**	**12**	**2680**	**0.0–8.8**	**2.1**	**1.6 (0.4–3.4)**	**81.8 (*p *< 0.001)**	**86.5 (78.3–91.7)**	**0.0–11.0**
Current anorectal infection	NAAT/PCR	9	2145	0.0–29.4	11.1	10.4 (4.6–18.0)	249.1 (*p* < 0.001)	96.8 (95.4–97.8)	0.0–44.5
	**Overall**	**9**	**2145**	**0.0–29.4**	**11.1**	**10.4 (4.6–18.0)**	**249.1 (*p* < 0.001)**	**96.8 (95.4–97.8)**	**0.0–44.5**
Unspecified/mixed anatomical site	Other/unclear assay^g^	1	2531	–	–	36.1 (34.2–38.0)	–	–	–
	**Overall**	**1**	**2531**	**–**	**–**	**36.1 (34.2–38.0)**	**–**	**–**	**–**
STI clinic attendees									
Current urogenital infection	NAAT/PCR	4	2313	0.2–3.4	0.5	0.8 (0.0–2.3)	18.2 (*p* < 0.001)	83.5 (58.2–93.5)	0.0–13.1
	Culture	5	7912	1.7–44.1	14.9	17.5 (4.6–36.5)	996.9 (*p* < 0.001)	99.6 (99.5–99.7)	0.0–92.4
	Gram stain	2	292	8.3–24.6	16.5	21.2 (16.7–26.4)^e^	–	–	–
	**Overall**	**11**	**10,517**	**0.2–44.1**	**7.1**	**9.0 (2.6–18.6)**	**1,885.2 (*p* < 0.001)**	**99.5 (99.4–99.6)**	**0.0–57.7**
Unspecified/mixed anatomical site	Culture	3	3077	6.0–13.0	6.7	8.6 (4.3–14.2)	45.3 (*p* < 0.001)	95.6 (90.3–98.0)	0.0–95.0
	Other/unclear assay^g^	4	2626	2.1–45.1	25.2	21.7 (5.9–43.9)	255.8 (*p* < 0.001)	98.8 (98.2–99.2)	0.0–100
	**Overall**	**7**	**5703**	**2.1–45.1**	**13.0**	**15.5 (6.2–28.0)**	**546.0 (*p* < 0.001)**	**98.9 (98.5–99.2)**	**0.0–67.3**
Individuals living with HIV and individuals in HIV–discordant couples									
Current urogenital infection	NAAT/PCR	4	71	0.0–18.0	4.5	4.4 (0.2–11.5)	2.5 (*p* = 0.474)	0.0 (0.0–84.7)	0.0–22.8
	Culture	2	41	0.0–23.3	11.7	17.0 (7.1–32.1)^e^	–	–	–
	**Overall**	**6**	**112**	**0.0–23.0**	**4.5**	**6.7 (0.9–15.7)**	**9.4 (*p* = 0.094)**	**46.8 (0.0–78.9)**	**0.0–36.6**
Unspecified/mixed anatomical site	Culture	2	806	1.2–6.3	3.8	4.8 (3.5–6.6)^e^	–	–	–
	**Overall**	**2**	**806**	**1.2–6.3**	**3.8**	**4.8 (3.5–6.6)**^**e**^	**–**	**–**	**–**

*Abbreviations*: *CI* Confidence interval, *HIV* Human immunodeficiency virus, *NAAT* Nucleic acid amplification test, *NG Neisseria gonorrhoeae*, *PCR* Polymerase chain reaction, *STI* Sexually transmitted disease

A minimum of three studies were required to conduct a meta-analysis

Bolded numbers represent overall pooled estimates

^a^ Population type classification can be found in Table 1

^b^*Q*: The Cochran’s *Q* statistic is a measure assessing the existence of heterogeneity in pooled outcome measures, here NG prevalence

^c^*I*^2^: A measure that assesses the magnitude of between-study variation that is due to actual differences in NG prevalence across studies rather than chance

^d^Prediction interval: A measure that estimates the distribution (95% interval) of true NG prevalence around the estimated mean

^e^Two prevalence measures are not sufficient to conduct a random-effects meta-analysis. The pooled measure was calculated as the arithmetic mean of the two measures and their 95% confidence intervals

^f^The majority of studies were on male sex workers, primarily from Pakistan, while a smaller proportion of studies were on men who have sex with men

^g^Other/unclear assay include enzyme immunoassay, indirect hemagglutination, or mixed/unclear testing technique

**Table 4:** Results of meta-analyses on studies reporting *Neisseria gonorrhoeae* prevalence in symptomatic populations and patients with confirmed or suspected STIs and related infections in the Middle East and North Africa

Population type^a^		Stratified prevalence measures	Sample size	NG prevalence (%)		Pooled mean NG prevalence	Heterogeneity measures		
		Total *n*	Total *N*	Range	Median	Mean (%) (95% CI)	*Q*^b^ (*p* value)	*I*^2c^ (%) (95% CI)	Prediction interval^d^ (%)
Symptomatic women									
Current urogenital infection	NAAT/PCR	30	8008	0.0–30.0	3.2	3.4 (2.0–5.2)	227.1 (p<0.001)	87.2 (82.9–90.5)	0.0–16.1
	Culture	27	8633	0.0–25.0	3.8	4.3 (2.4–6.7)	466.8 (p<0.001)	94.4 (92.9–95.6)	0.0–21.6
	Gram stain	14	2028	0.0–38.1	2.6	3.8 (0.8–8.7)	195.0 (p<0.001)	93.3 (90.4–95.3)	0.0–32.2
	Wet mount	5	387	0.0–14.3	5.3	3.1 (0.0–10.0)	9.6 (p=0.048)	58.3 (0.0–84.5)	0.0–31.2
	Other/unclear assay^e^	2	446	0.0–26.0	13.0	2.7 (1.4–4.6)^f^	–	–	–
	**Overall**	**78**	**19,502**	**0.0–38.1**	**3.1**	**3.9 (2.7–5.3)**	**965.3 (p<0.001)**	**92.0 (90.7–93.2)**	**0.0–20.5**
Current anorectal infection	Culture	1	200	–	–	1.2 (1.1–6.4)	–	–	–
	**Overall**	**1**	**200**	**–**	**–**	**1.2 (1.1–6.4)**	**–**	**–**	**–**
Unspecified/mixed anatomical site	NAAT/PCR	1	441	–	–	0.9 (0.2–2.3)	–	–	–
	Culture	1	400	–	–	19.2 (15.5–23.5)	–	–	–
	Other/unclear assay^e^	3	447	1.4–5.0	4.0	3.3 (1.3–6.1)	3.6 (p=0.162)	45.1 (0.0–83.7)	0.0–54.5
	**Overall**	**5**	**1288**	**1.0–19.2**	**4.0**	**4.8 (0.8–11.7)**	**115.8 (p<0.001)**	**96.5 (94.2–98.0)**	**0.0–42.7**
Symptomatic men									
Current urogenital infection	NAAT/PCR	7	1130	11.4–63.0	40.0	39.2 (27.1–52.1)	76.1 (p<0.001)	92.4 (86.9–95.6)	4.4–82.3
	Culture	26	5109	2.0–94.0	41.5	41.6 (30.9–52.8)	1,648.7 (p<0.001)	98.5 (98.2–98.7)	0.6–93.1
	Gram stain	33	11,003	3.5–96.0	46.0	44.6 (34.9–54.4)	2,286.4 (p<0.001)	98.6 (98.4–98.8)	1.7–94.1
	Other/unclear assay^e^	3	460	0.6–28.0	26.8	14.9 (0.7–41.4)	89.6 (p<0.001)	97.8 (95.8–98.8)	0.0–100
	**Overall**	**69**	**17,702**	**0.6–96.0**	**43.0**	**41.4 (34.9–48.1)**	**4,471.2 (p<0.001)**	**98.5 (98.3–98.6)**	**1.6–90.7**
Unspecified/mixed anatomical site	NAAT/PCR	1	422	–	–	41.7 (36.9–46.6)	–	–	–
	Gram stain	1	162	–	–	67.3 (59.5–74.4)	–	–	–
	**Overall**	**2**	**584**	**41.6–67.3**	**54.5**	**48.8 (44.7–52.9)**^**f**^	**–**	**–**	**–**
Symptomatic patients (mixed sexes)									
Current urogenital infection	NAAT/PCR	1	168	–	–	22.6 (16.5–29.7)	–	–	–
	Culture	1	95	–	–	26.3 (17.8–36.3)	–	–	–
	**Overall**	**2**	**263**	**23.0–26.3**	**24.7**	**23.9 (18.9–29.5)**^**f**^	**–**	**–**	**–**
Patients with confirmed or suspected STIs and related infections									
Current urogenital infection	NAAT/PCR	4	335	3.8–96.2	12.5	29.0 (0.0–79.7)	280.5 (p<0.001)	98.9 (98.4–99.3)	0.0–100
	Culture	7	1174	4.7–61.3	21.7	26.4 (11.1–45.4)	315.7 (p<0.001)	98.1 (97.3–98.7)	0.0–91.2
	Gram stain	2	160	55.0–93.7	74.4	74.4 (66.9–80.9)^f^	–	–	–
	**Overall**	**13**	**1669**	**3.8–96.3**	**21.7**	**34.8 (16.2–56.2)**	**835.1 (p<0.001)**	**98.6 (98.2–98.9)**	**0.0–100**

*Abbreviations*: *CI* Confidence interval, *NAAT* Nucleic acid amplification test, *NG Neisseria gonorrhoeae*, *PCR* Polymerase chain reaction, *STI* Sexually transmitted infection

A minimum of three studies were required to conduct a meta-analysis

Bolded numbers represent overall pooled estimates

^a^Population type classification can be found in Table 1

^b^*Q*: The Cochran’s *Q* statistic is a measure assessing the existence of heterogeneity in pooled outcome measures, here NG prevalence

^c^*I*^2^: A measure that assesses the magnitude of between–study variation that is due to actual differences in NG prevalence across studies rather than chance

^d^Prediction interval: A measure that estimates the distribution (95% interval) of true NG prevalence around the estimated mean

^e^Other/unclear assay include enzyme immunoassay, indirect hemagglutination, or mixed/unclear testing technique

^f^Two prevalence measures are not sufficient to conduct a random-effects meta-analysis. The pooled measure was calculated as the arithmetic mean of the two measures and their 95% confidence intervals

### Precision and risk of *bias* assessments

The study-specific precision and ROB assessments are summarized in Additional file 1: Table S6. Among the included studies, 189 studies (55.4%) had sample sizes of < 200 participants, indicating low precision. Meanwhile, 291 studies (85.3%) utilized non-probability-based (convenience) sampling, particularly those conducted in clinical settings (Additional file 1: Table S3 and Table S4). Remarkably, 61.5% of studies focusing on high-risk populations, including female sex workers (FSWs), male sex workers (MSWs), and men who have sex with men (MSM), employed probability-based sampling methods, often utilizing respondent-driven sampling.

The response rate was unclear in 158 studies (46.3%), and 15 studies (4.4%) were identified as having high ROB in terms of this quality domain. Only 23 studies (6.7%) demonstrated low ROB in both quality domains, while none had high ROB in both quality domains.

### Pooled mean estimates of gonorrhea prevalence

Pooled mean NG prevalence by population type, anatomical site, and assay type is summarized in Tables 2, 3 and 4. For current urogenital infection, the pooled prevalence was 1.9% (95% CI 1.1–2.8%) among general populations, 7.5% (95% CI 2.8–14.0%) among infertility clinic attendees, 6.5% (95% CI 4.4–9.0%) among FSWs, 9.0% (95% CI 2.6–18.6%) among STI clinic attendees, 3.9% (95% CI 2.7–5.3%) among symptomatic women, and 41.4% (95% CI 34.9–48.1%) among symptomatic men. Among MSWs and MSM, the pooled prevalence for current urogenital infection was 1.6% (95% CI 0.4–3.4%), and for current anorectal infection, it was 10.4% (95% CI 4.6–18.0%).

**Fig. 2:**
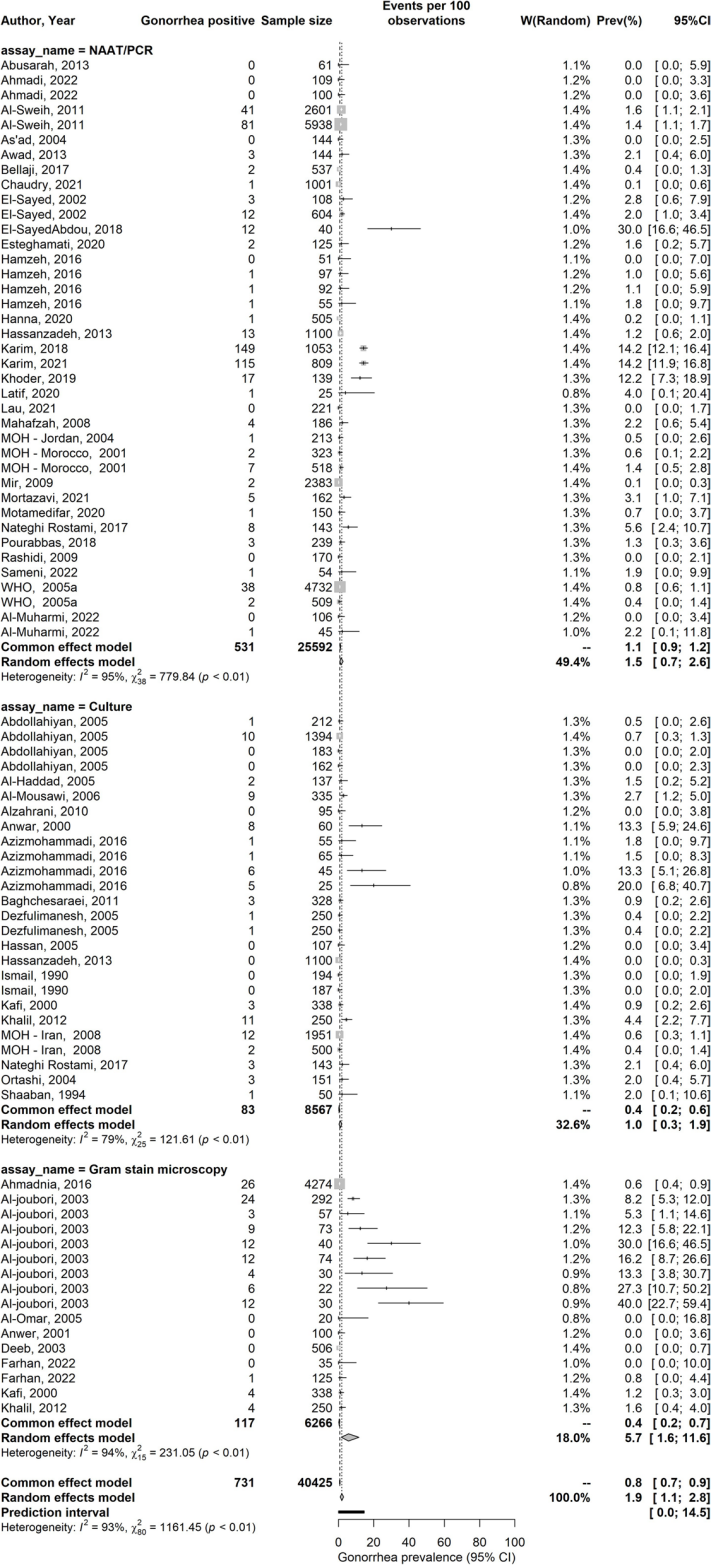
Forest plot of pooled mean prevalence of *Neisseria gonorrhoeae* in urogenital specimens among general populations in the Middle East and North Africa *Abbreviations:* *NAAT* Nucleic acid amplification test, *PCR* Polymerase chain reaction

Additional file 1: Table S5 summarizes the pooled mean urogenital NG prevalence among general populations, stratified by both MENA country and study precision. The pooled prevalence exhibited variation across MENA countries. Studies with sample sizes of 200 participants or more yielded a pooled prevalence of 1.1% (95% CI: 0.5-1.9%), whereas studies with smaller sample sizes (< 200 participants) had a higher pooled prevalence of 3.1% (95% CI: 1.5-5.0%). Forest plots of the meta-analyses can be found in Fig. 2 and in Additional file 1: Figure S1 and Figure S2. Most meta-analyses demonstrated significant heterogeneity (*p* value < 0.1), primarily attributed to true variation in prevalence rather than chance (*I*^2^ > 50%) (Tables 2, 3 and 4). This observation was further confirmed by wide prediction intervals, indicating considerable variability in NG prevalence across the studies.

### Predictors of prevalence and sources of between-study heterogeneity

To explore potential associations and explain the observed between-study heterogeneity in urogenital NG prevalence measures, univariable and multivariable meta-regression analyses were conducted. The results of these analyses are presented in Table 5. Two multivariable models were utilized: one with the year of data collection as a categorical variable and another with it as a linear term. To address collinearity issues, sensitivity analyses were performed by including the year of publication instead of the year of data collection (Additional file 1: Table S7), and by incorporating national income instead of the MENA subregion (Additional file 1: Table S8).

**Table 5:** Univariable and multivariable meta-regression analyses for *Neisseria gonorrhoeae* prevalence in urogenital specimens in the Middle East and North Africa using subregion and year of data collection variables

Urogenital specimens			Stratified prevalence measures	Sample size	Univariable analysis				Multivariable analyses			
			Total *n*	Total *N*	RR (95% CI)	*p* value	LT test *p*-value	Adjusted *R*^2^	Model 1		Model 2	
									ARR (95% CI)	*p* value	ARR (95% CI)	*p* value
Population characteristics	Population type^a^	General populations	81	40,425	1.00	–	< 0.001	21.3	1.00	–	1.00	–
		Intermediate-risk populations	14	4227	0.61 (0.27–1.41)	0.250			0.46 (0.19–1.11)	0.085	0.46 (0.19–1.10)	0.082
		FSWs	22	7363	2.61 (1.48–4.61)	0.001			3.50 (2.05–5.99)	< 0.001	3.27 (1.90–5.61)	< 0.001
		MSWs and MSM^b^	12	2680	1.53 (0.63–3.71)	0.346			0.83 (0.32–2.15)	0.704	0.83 (0.33–2.14)	0.705
		Symptomatic women	78	19,502	1.65 (1.09–2.49)	0.017			1.81 (1.22–2.68)	0.003	1.79 (1.22–2.64)	0.003
		Symptomatic men	69	17,702	14.50 (9.80–21.70)	< 0.001			6.63 (3.52–12.40)	< 0.001	6.56 (3.50–12.20)	< 0.001
		Symptomatic patients (mixed sexes)	2	263	10.30 (2.23–47.50)	0.003			7.35 (1.78–30.20)	0.006	7.31 (1.79–29.80)	0.006
		Infertility clinic attendees	31	3508	3.70 (2.09–6.53)	< 0.001			2.92 (1.68–5.08)	< 0.001	2.90 (1.67–5.03)	< 0.001
		Women with miscarriage or ectopic pregnancy	5	420	1.86 (0.53–6.52)	0.331			1.72 (0.55–5.40)	0.348	1.66 (0.54–5.14)	0.376
		STI clinic attendees	11	10,517	2.37 (1.13–4.97)	0.022			2.56 (1.23–5.32)	0.012	2.70 (1.31–5.58)	0.007
		Individuals living with HIV and individuals in HIV-discordant couples	6	112	4.38 (1.20–16.00)	0.026			3.56 (1.07–11.80)	0.039	3.57 (1.08–11.80)	0.038
		Patients with confirmed or suspected STIs and related infections	13	1669	10.00 (5.12–19.50)	< 0.001			6.36 (3.19–12.70)	< 0.001	6.48 (3.26–12.80)	< 0.001
		Other populations^c^	4	4302	0.93 (0.26–3.33)	0.916			0.73 (0.20–2.63)	0.633	0.78 (0.22–2.80)	0.707
	Age group	< 25 years	11	807	1.00	–	0.529	0.0	–	–	–	–
		25–34 years	6	505	4.02 (0.72–22.30)	0.112			–	–	–	–
		35–44 years	4	400	2.09 (0.30–14.50)	0.456			–	–	–	–
		≥ 45 years	5	193	1.74 (0.20–15.30)	0.615			–	–	–	–
		Mixed ages	322	110,785	1.41 (0.44–4.45)	0.560			–	–	–	–
	Sex	Women	211	70,869	1.00	–	< 0.001	29.5	1.00	–	1.00	-
		Men	125	39,772	5.14 (3.75–7.03)	< 0.001			1.77 (1.09–2.87)	0.020	1.71 (1.06-2.76)	0.029
		Mixed sexes	12	2049	1.20 (0.49–2.95)	0.684			1.15 (0.51–2.62)	0.730	1.06 (0.47-2.36)	0.892
	MENA Subregions	Fertile crescent	109	16,834	1.00	–	< 0.001	7.5	1.00	–	1.00	–
		Horn of Africa	23	10,904	0.64 (0.31–1.34)	0.240			0.59 (0.35–1.00)	0.050	0.58 (0.34-0.98)	0.042
		Gulf	33	26,421	1.87 (1.01–3.45)	0.047			1.27 (0.80–2.00)	0.310	1.29 (0.82-2.03)	0.261
		Maghreb	44	12,155	2.01 (1.17–3.46)	0.011			1.52 (1.01–2.30)	0.046	1.44 (0.95-2.18)	0.082
		Iran	96	28,675	0.59 (0.37–0.94)	0.025			0.95 (0.67–1.35)	0.775	0.94 (0.67-1.32)	0.711
		Pakistan and Afghanistan	40	17,701	0.68 (0.37–1.25)	0.219			1.15 (0.70–1.90)	0.581	1.21 (0.74-1.98)	0.444
	National income	LIC	9	8819	1.00	–	< 0.001^d^	6.7	–	–	–	–
		LMIC	122	34,546	8.69 (2.65–28.50)	< 0.001			–	–	–	–
		UMIC	181	42,904	5.52 (1.69–17.90)	0.005			–	–	–	–
		HIC	36	26,421	13.30 (3.74–47.60)	< 0.001			–	–	–	–
Study methodology characteristics	Assay type	NAAT/PCR	144	51,503	1.00	–	< 0.001	14.4	1.00	–	1.00	–
		Culture	117	34,795	2.08 (1.41–3.07)	< 0.001			1.18 (0.85–1.64)	0.307	1.14 (0.82–1.58)	0.429
		Gram stain	75	21,069	4.62 (2.99–7.14)	< 0.001			2.10 (1.43–3.07)	< 0.001	1.87 (1.24–2.81)	0.003
		Wet mount	5	387	1.25 (0.26–6.01)	0.779			1.00 (0.30-3.34)	0.998	1.01 (0.31–3.35)	0.982
		Other/unclear	7	4936	2.14 (0.63–7.22)	0.221			0.92 (0.35–2.41)	0.859	0.84 (0.32–2.22)	0.730
	Sample size	< 200	187	15,748	1.00	–	< 0.001	6.1	1.00	–	1.00	–
		≥ 200	161	96,942	0.48 (0.34–0.68)	< 0.001			0.40 (0.31–0.52)	< 0.001	0.39 (0.30–0.50)	< 0.001
	Sampling method	Probability based	42	22,262	1.00	–	0.004	2.8	1.00	–	1.00	–
		Non-probability based	306	90,428	2.30 (1.30–4.06)	0.004			0.63 (0.39–1.01)	0.057	0.62 (0.39–1.00)	0.051
	Response rate	≥ 80%	170	61,786	1.00	–	< 0.001	6.1	1.00	–	1.00	–
		< 80%	10	4304	0.08 (0.02–0.32)	< 0.001			0.14 (0.05–0.40)	< 0.001	0.14 (0.05–0.41)	< 0.001
		Unclear	168	46,600	0.55 (0.39–0.78)	0.001			1.28 (0.95–1.71)	0.103	1.30 (0.97–1.75)	0.075
Temporal trend	Year of data collection category	< 2000	102	26,032	1.00	–	< 0.001	11.4	1.00	–	–	–
		2000–2009	127	54,690	0.37 (0.24–0.56)	< 0.001			0.88 (0.64–1.22)	0.454	–	–
		≥ 2010	119	31,968	0.30 (0.20–0.46)	< 0.001			0.73 (0.50–1.08)	0.114	–	–
	Year of data collection		348	112,690	0.96 (0.94–0.97)	< 0.001	< 0.001	14.4	–	–	0.99 (0.97–1.00)	0.033

*Abbreviations: ARR* Adjusted risk ratio, *CI* Confidence interval, *FSWs* Female sex workers, *HIC* High-income country, *HIV* Human immunodeficiency virus, *MENA* Middle East and North Africa, *MSM* Men who have sex with men, *MSWs* Male sex workers, *NAAT* Nucleic acid amplification test, *LIC* Low income country, *LMIC* Low-middle income country, *LT test* Likelihood ratio test, *PCR* Polymerase chain reaction, *RR* Risk ratio, *STI* Sexually transmitted infection, *UMIC* Upper-middle income country

Adjusted R^2^ in the final multivariable model 1 =63.98%

Adjusted R^2^ in the final multivariable model 2 =64.42%

^a^Population type classification can be found in Table 1

^b^The majority of studies were on male sex workers, primarily from Pakistan, while a smaller proportion of studies were on men who have sex with men

^c^Other populations include populations with an undetermined risk of acquiring *Neisseria gonorrhoeae* infection such as victims of sexual assault and mixed populations, among others

^d^National income was not included in the multivariable model due to collinearity with MENA subregion variable

The main analyses and sensitivity analyses produced similar results, collectively explaining approximately 64% of the variation in prevalence across the studies. Compared to general populations, the highest prevalence levels were observed among specific groups, including symptomatic patients, individuals with confirmed/suspected STIs, individuals living with HIV and individuals in HIV-discordant couples, attendees of infertility clinics, and FSWs (Table 5).

Prevalence of urogenital NG was higher in men compared to women and was especially higher among symptomatic men compared to symptomatic women (Table 5). Evidence suggested subregional variability, with low-income countries showing lower prevalence rates than higher-income countries (Additional file 1: Table S8). No significant differences in prevalence were observed based on age group. Prevalence declined at a rate of 1% per year.

Regarding the effects of study methods on prevalence, a higher prevalence was observed when NG was tested using Gram stain compared to NAAT or culture (Table 5). Studies with a response rate < 80% reported lower prevalence levels than those with a response rate ≥ 80%. A small-study effect was identified; studies having a sample size ≥ 200 reported approximately 60% lower prevalence. Though no statistically significant evidence was found for differences in prevalence based on the sampling method, there was a tendency for prevalence to be lower in non-probability-based samples.

## Discussion

Despite the sexually conservative norms and relatively low levels of viral STIs in MENA [26, 29, 42, 95, 96], the prevalence of NG in the general population was unexpectedly high at 1.9%. This prevalence level was higher than the global average at 0.8% but with overlapping 95% CIs [7]. The elevated NG prevalence aligns with the higher-than-expected prevalence of chlamydia [44], trichomoniasis [97], and syphilis [98] recently observed in the region. These findings suggest a significant but often overlooked bacterial and other curable STI disease burden in MENA, which may have substantial social and economic implications, particularly in the absence of adequate sexual health and STI programs [25–31]. Evidence suggests a decline in prevalence, albeit at a slow pace of approximately 1% per year. This rate of decline is far below what is sufficient to meet the WHO's target of reducing NG incidence by 90% by 2030.

The elevated NG prevalence suggests the presence of active transmission networks for NG and other STIs, but it may not necessarily indicate elevated levels of risky sexual behaviors. Rather, it could be attributed, in part, to inadequate access to and utilization of STI services. MENA faces limited capacity in terms of STI prevention and treatment [25–31]. Similar observations elsewhere have shown that limited bacterial STI diagnosis and specific treatment can lead to unusually high prevalence rates [99–101]. This is particularly relevant considering that NG infection is often asymptomatic, and if left untreated, can result in prolonged shedding, increasing the potential for transmission within the population.

Similar to chlamydia in MENA [44], the prevalence of NG was three times higher among attendees of infertility clinics and twice as high among women with miscarriages or ectopic pregnancies, compared to the general population. However, the latter effect size did not reach statistical significance, perhaps because of the relatively small number of studies. This contrasts with developed regions like Europe, where infection rates among infertility clinic attendees are similar to those in the general population [6, 35].

MENA has been reported to have the highest rate of primary infertility globally, a phenomenon that is not yet adequately understood [102]. In a cultural context where infertility has important socio-cultural consequences for women and their families [103, 104], it is plausible to consider NG infection as a poorly recognized cause of infertility in this region [105–108]. While the consequences of this mostly asymptomatic infection among women [4] may not be readily apparent, its impact on reproductive health outcomes could be visible, even if not explicitly linked to the underlying cause [44]. However, distinguishing the specific role of gonorrhea from that of chlamydia or other factors in different reproductive outcomes remains challenging [6, 109, 110].

The prevalence of NG infection followed a hierarchical pattern, with higher rates observed in higher-risk populations, such as FSWs, aligning with patterns seen in other STIs [28, 36, 42, 44, 111]. NG infection is often associated with recent risky sex [3, 32, 112], including frequent turnover in sexual partnerships and engagement in transactional sex [3, 4, 33, 34, 113, 114]. These findings suggest the existence of cores of risky sexual behaviors that are able to sustain NG transmission. This is further supported by data from MENA, which indicate the common occurrence of payment for sex among STI clinic attendees [115, 116], as well as considerable levels of sexual risk behavior among key populations [28, 43, 117, 118], where emerging and growing HIV epidemics are also observed [28, 43, 117–119]. These findings underscore the importance of understanding sexual behavior and sexual networks in both key populations and the general population in this region.

The prevalence of anorectal NG among MSWs and MSM was found to be high, at approximately 10%. This finding confirms that these populations are at a heightened risk of infection. However, only nine studies were available for this specific anatomical site within these specific populations, and they were conducted exclusively in Pakistan and Morocco. Therefore, these findings may not be representative of the broader MENA region.

As anticipated, the prevalence of NG infection was high among symptomatic individuals, especially men, and those with suspected exposure to STIs. This observation aligns with a more symptomatic course of NG infection in men [105, 120] and emphasizes the significant role of NG in causing urethritis in MENA. These findings also underscore the importance of conducting gonococcal AMR surveillance, particularly considering the limited evidence available on this global priority in this region [121–123].

This study is subject to limitations. The quality and quantity of available data varied across countries, population types, and anatomical sites. Data was not found for Qatar and Syria, and only limited data was available for Afghanistan, Algeria, Libya, and Palestine. There was a scarcity of data regarding anorectal and oropharyngeal NG infections. The majority of identified studies focused on reporting measures for urogenital NG infection among general populations, symptomatic women, and symptomatic men. Conversely, only a small proportion of studies examined key populations such as FSWs and MSM, who are most affected by NG infection.

NG exhibits a low prevalence in general populations worldwide [7, 35]. With a global prevalence estimated at only 0.8% [7], fewer than one in every 100 tests will detect a positive case. Consequently, studies with relatively small sample sizes often fail to detect any infections due to sampling variation. Among studies reporting urogenital NG prevalence in MENA, 13.3% reported zero prevalence, often because of insufficient sample sizes to detect such a low-prevalence infection. Notably, about half of the studies included fewer than 200 participants, highlighting the critical need for large sample sizes to accurately measure NG prevalence in general populations.

However, by pooling studies through the meta-analyses in this work, the limitation of inadequate sample size is partly mitigated by leveraging the collective statistical power of a large meta-analysis sample size, which combines the sample sizes of the individual studies. Furthermore, the meta-regression analyses quantified the effect of sample size on observed prevalence and revealed a small study effect. Specifically, studies with a sample size of 200 or more reported prevalences approximately 60% lower than those of smaller studies. This finding is likely due to publication bias, where studies reporting zero or very low prevalence are less likely to be published than those reporting higher prevalence.

While this study identified a substantial volume of data, caution is warranted when interpreting the findings. Heterogeneity in prevalence was observed across the analyzed studies; however, most of this heterogeneity was subsequently explained by epidemiological factors or study methods through meta-regression analyses. Variations were observed in assay types, sampling methods, and response rates among the studies. These factors were found to be associated with the reported prevalence, indicating methodological limitations in the available studies. The use of diagnostic assays varied over time, and convenience sampling was predominantly used instead of probability-based sampling.

Studies with lower-quality methods tended to report higher NG prevalence, while studies of higher-quality methods reported lower prevalence. Some studies reported unusually high values even in populations presumed to have a low risk of infection, suggesting the presence of unreported bias in sample recruitment or potential unidentified issues in laboratory methods. Inadequate descriptions of factors such as response rate, sampling method, or laboratory methods were observed among the studies. These limitations indicate that the findings may not fully capture the true prevalence and distribution of NG infections across MENA, and the reported pooled measures may overestimate the true NG prevalence.

These limitations highlight the need for improved study methods in investigating gonorrhea and other STIs in MENA. Implementing high-quality, population-based studies that employ probability-based sampling techniques, standardized protocols, and sensitive and specific diagnostic assays is critical to overcoming these limitations. Such improvements are essential to obtain a more representative picture of NG epidemiology in MENA.

Despite the limitations, the study identified a substantial volume of data, including published and unpublished sources, providing a detailed investigation of NG epidemiology in MENA for the first time. The study’s diverse results and analytics shed light on NG epidemiology in various populations and settings. The findings inform the development and expansion of STI and sexual health programs, inform gonococcal AMR surveillance, and identify priority populations for NG vaccination in MENA.

## Conclusions

In conclusion, NG prevalence in MENA is comparable to the global average prevalence, highlighting a neglected and underrecognized disease burden with potential social and economic implications. Urgent action is needed to address NG transmission and disease burden in MENA, as the current response falls far short of the WHO’s Global Health Sector Strategy on STIs. Lingering STI stigma, along with political and socio-cultural sensitivities, hampers progress in establishing an inclusive public health agenda and supportive environment for sexual health. To confront the STI burden effectively, targeted, culturally sensitive, and gender-specific programs must be developed. Integrating STIs with established HIV surveillance programs for key populations in the region [124, 125] is a practical approach that merits consideration [126–128]. The urgency of accelerating NG vaccine development is underscored by the findings, as the vaccine may provide a fundamental solution to address this infection and its drug resistance in MENA and beyond.

## Supplementary Information

Additional file 1: Contains supplementary data and analyses as follows: Table S1. Preferred Reporting Items for Systematic Reviews and Meta-analyses (PRISMA) checklist. Table S2. Data sources and search strategies for systematically reviewing *Neisseria gonorrhoeae* epidemiology in the Middle East and North Africa. Box S1. Countries included in the Middle East and North Africa definition and their subregional classification. Box S2. Variables extracted from relevant records meeting the inclusion criteria.  Box S3. Factors (variables) selected a priori and included in the univariable and multivariable meta-regression analyses. Table S3. Studies reporting *Neisseria gonorrhoeae* prevalence in urogenital specimens in the Middle East and North Africa. Table S4. Studies reporting *Neisseria gonorrhoeae* prevalence in anorectal, oropharyngeal, unspecified, or mixed anatomical sites, or serological specimens in the Middle East and North Africa. Table S5. Results of meta-analyses on studies reporting urogenital *Neisseria gonorrhoeae* prevalence in general populations by MENA country, and study precision. Table S6. Summary of precision assessment and risk of bias assessment for studies reporting *Neisseria gonorrhoeae* prevalence in the Middle East and North Africa. Figure S1. Forest plots presenting outcomes of the pooled mean *Neisseria gonorrhoeae* prevalence in urogenital specimens among different populations in the Middle East and North Africa. Figure S2. Forest plots presenting outcomes of the pooled mean *Neisseria gonorrhoeae* prevalence in anorectal, oropharyngeal, unspecified or mixed anatomical sites, or serological specimens among different populations in the Middle East and North Africa.          

## Data Availability

The dataset supporting the conclusions of this article is included within the article and its Supplementary Material.

## Abbreviations

AMR: Antimicrobial resistance
CI: Confidence interval
FSW: Female sex workers
MENA: Middle East and North Africa
MSM: Men who have sex with men
MSW: Male sex workers
NG: *Neisseria* *gonorrhoeae*
PRISMA: Preferred Reporting Items for Systematic Reviews and Meta-analyses
ROB: Risk of bias
STI: Sexually transmitted infection
WHO: World Health Organization
