# Supplementary material for: Epidemiology of gonorrhea in countries of the Middle East and North Africa: systematic review, meta analyses, and meta regressions
Source: BMC Glob Public Health. 2024 Aug 19;2:56. doi: 10.1186/s44263-024-00088-9 (PMC11622951; doi:10.1186/s44263-024-00088-9)
Supplement: Supplementary file 1 — Additional file 1: Contains supplementary data and analyses as follows: Table S1. Preferred Reporting Items for Systematic Reviews and Meta-analyses (PRISMA) checklist. Table S2. Data sources and search strategies for systematically reviewing Neisseria gonorrhoeae epidemiology in the Middle East and North Africa. Box S1. Countries included in the Middle East and North Africa definition and their subregional classification. Box S2. Variables extracted from relevant records meeting the inclusion criteria. Box S3. Factors (variables) selected a priori and included in the univariable and multivariable meta-regression analyses. Table S3. Studies reporting Neisseria gonorrhoeae prevalence in urogenital specimens in the Middle East and North Africa. Table S4. Studies reporting Neisseria gonorrhoeae prevalence in anorectal, oropharyngeal, unspecified, or mixed anatomical sites, or serological specimens in the Middle East and North Africa. Table S5. Results of meta-analyses on studies reporting urogenital Neisseria gonorrhoeae prevalence in general populations by MENA country, and study precision. Table S6. Summary of precision assessment and risk of bias assessment for studies reporting Neisseria gonorrhoeae prevalence in the Middle East and North Africa. Figure S1. Forest plots presenting outcomes of the pooled mean Neisseria gonorrhoeae prevalence in urogenital specimens among different populations in the Middle East and North Africa. Figure S2. Forest plots presenting outcomes of the pooled mean Neisseria gonorrhoeae prevalence in anorectal, oropharyngeal, unspecified or mixed anatomical sites, or serological specimens among different populations in the Middle East and North Africa. [file 44263_2024_88_MOESM1_ESM.docx]

**Additional file 1**

Table of Contents

[**Table S1.** Preferred Reporting Items for Systematic Reviews and Meta-analyses (PRISMA) checklist [1, 2]. 3](#_Toc172803170)

[**Table S2.** Data sources and search strategies for systematically reviewing *Neisseria gonorrhoeae* epidemiology in the Middle East and North Africa. 6](#_Toc172803171)

[**Box S1.** Countries included in the Middle East and North Africa definition and their subregional classification. 7](#_Toc172803172)

[**Box S2.** Variables extracted from relevant records meeting the inclusion criteria. 8](#_Toc172803173)

[**Box S3.** Factors (variables) selected *a priori* and included in the univariable and multivariable meta-regression analyses. 9](#_Toc172803174)

[**Table S3.** Studies reporting *Neisseria gonorrhoeae* prevalence in urogenital specimens in the Middle East and North Africa. 10](#_Toc172803175)

[**Table S4.** Studies reporting *Neisseria gonorrhoeae* prevalence in anorectal, oropharyngeal, unspecified, or mixed anatomical sites, or serological specimens in the Middle East and North Africa. 16](#_Toc172803176)

[**Table S5.** Results of meta-analyses on studies reporting urogenital *Neisseria gonorrhoeae* prevalence in general populations by MENA country, and study precision. 18](#_Toc172803177)

[**Table S6.** Summary of precision assessment and risk of bias assessment for studies reporting *Neisseria gonorrhoeae* prevalence in the Middle East and North Africa. 19](#_Toc172803178)

[**Fig S1.** Forest plots presenting outcomes of the pooled mean *Neisseria gonorrhoeae* prevalence in urogenital specimens among different populations in the Middle East and North Africa. 20](#_Toc172803179)

[**Fig S2.** Forest plots presenting outcomes of the pooled mean *Neisseria gonorrhoeae* prevalence in anorectal, oropharyngeal, unspecified or mixed anatomical sites, or serological specimens among different populations in the Middle East and North Africa. 31](#_Toc172803180)

[**Table S7.** Sensitivity analysis. Univariable and multivariable meta-regression analyses for *Neisseria gonorrhoeae* prevalence in urogenital specimens in the Middle East and North Africa using the year of publication as the time variable instead of the year of data collection. 34](#_Toc172803181)

[**Table S8.** Sensitivity analysis. Univariable and multivariable meta-regression analyses for *Neisseria gonorrhoeae* prevalence in urogenital specimens in the Middle East and North Africa (MENA) using national income instead of MENA subregion as the analysis variable. 36](#_Toc172803182)

[**References** 38](#_Toc172803183)

# **Table S1.** Preferred Reporting Items for Systematic Reviews and Meta-analyses (PRISMA) checklist [1, 2].

| **Section and topic** | **Item #** | **Checklist item** | **Location where item is reported** |
| --- | --- | --- | --- |
| **Title** | | |  |
| Title | 1 | Identify the report as a systematic review. | Title |
| **Abstract** | | |  |
| Abstract | 2 | See the PRISMA 2020 for Abstracts checklist (table 2). | Summary section |
| **Introduction** | | |  |
| Rationale | 3 | Describe the rationale for the review in the context of existing knowledge. | Introduction section |
| Objectives | 4 | Provide an explicit statement of the objective(s) or question(s) the review addresses. | Introduction section |
| **Methods** | | |  |
| Eligibility criteria | 5 | Specify the inclusion and exclusion criteria for the review and how studies were grouped for the syntheses. | Methods: Study selection and inclusion and exclusion criteria section |
| Information sources | 6 | Specify all databases, registers, websites, organisations, reference lists and other sources searched or consulted to identify studies. Specify the date when each source was last searched or consulted. | Methods: Data sources and search strategy section; Table S2 |
| Search strategy | 7 | Present the full search strategies for all databases, registers and websites, including any filters and limits used. | Table S2 |
| Selection process | 8 | Specify the methods used to decide whether a study met the inclusion criteria of the review, including how many reviewers screened each record and each report retrieved, whether they worked independently, and if applicable, details of automation tools used in the process. | Methods: Study selection process and inclusion and exclusion criteria section |
| Data collection process | 9 | Specify the methods used to collect data from reports, including how many reviewers collected data from each report, whether they worked independently, any processes for obtaining or confirming data from study investigators, and if applicable, details of automation tools used in the process. | Methods: Data extraction and data synthesis section |
| Data items | 10a | List and define all outcomes for which data were sought. Specify whether all results that were compatible with each outcome domain in each study were sought (e.g., for all measures, time points, analyses), and if not, the methods used to decide which results to collect. | Methods: Data extraction and data synthesis section; Boxes S2 and S3 |
|  | 10b | List and define all other variables for which data were sought (e.g., participant and intervention characteristics, funding sources). Describe any assumptions made about any missing or unclear information. | Boxes S2 and S3 |
| Study risk of bias assessment | 11 | Specify the methods used to assess risk of bias in the included studies, including details of the tool(s) used, how many reviewers assessed each study and whether they worked independently, and if applicable, details of automation tools used in the process. | Methods: Precision and risk of bias assessments section |
| Effect measures | 12 | Specify for each outcome the effect measure(s) (e.g. risk ratio, mean difference) used in the synthesis or presentation of results. | Methods: Data extraction and data synthesis section; Boxes S2 and S3 |
| Synthesis methods | 13a | Describe the processes used to decide which studies were eligible for each synthesis (e.g. tabulating the study intervention characteristics and comparing against the planned groups for each synthesis (item #5)). | Methods: Meta-analyses and Meta-regressions sections; Boxes S2 and S3 |
|  | 13b | Describe any methods required to prepare the data for presentation or synthesis, such as handling of missing summary statistics, or data conversions. | Methods: Meta-analyses and Meta-regressions sections; Boxes S2 and S3 |
|  | 13c | Describe any methods used to tabulate or visually display results of individual studies and syntheses. | Methods: Meta-analyses and Meta-regressions sections; Boxes S2 and S3 |
|  | 13d | Describe any methods used to synthesise results and provide a rationale for the choice(s). If meta-analysis was performed, describe the model(s), method(s) to identify the presence and extent of statistical heterogeneity, and software package(s) used. | Methods: Meta-analyses and Meta-regressions sections; Boxes S2 and S3 |
|  | 13e | Describe any methods used to explore possible causes of heterogeneity among study results (e.g. subgroup analysis, metaregression). | Methods: Meta-analyses and Meta-regressions sections; Boxes S2 and S3 |
|  | 13f | Describe any sensitivity analyses conducted to assess robustness of the synthesised results. | Methods: Meta-analyses and Meta-regressions sections; Boxes S2 and S3 |
| Reporting bias assessment | 14 | Describe any methods used to assess risk of bias due to missing results in a synthesis (arising from reporting biases). | N/A |
| Certainty assessment | 15 | Describe any methods used to assess certainty (or confidence) in the body of evidence for an outcome. | N/A |
| **Results** | | |  |
| Study selection | 16a | Describe the results of the search and selection process, from the number of records identified in the search to the number of studies included in the review, ideally using a flow diagram (see fig 1). | Results: Search results and scope of evidence section; Fig 1 |
|  | 16b | Cite studies that might appear to meet the inclusion criteria, but which were excluded, and explain why they were excluded. | Figure 1 |
| Study characteristics | 17 | Cite each included study and present its characteristics. | Results: Gonorrhea prevalence overview section; Tables S3 and S4 |
| Risk of bias in studies | 18 | Present assessments of risk of bias for each included study. | Results: Precision and risk of bias assessments; Table S6 |
| Results of individual studies | 19 | For all outcomes, present, for each study: (a) summary statistics for each group (where appropriate) and (b) an effect estimate and its precision (e.g. confidence/credible interval), ideally using structured tables or plots. | Tables 2-4; Tables S3, S4, and S5; Fig 2; Fig S1 and Fig S2 |
| Results of syntheses | 20a | For each synthesis, briefly summarise the characteristics and risk of bias among contributing studies. | Results: Precision and risk of bias assessments; Table S6 |
|  | 20b | Present results of all statistical syntheses conducted. If meta-analysis was done, present for each the summary estimate and its precision (e.g. confidence/credible interval) and measures of statistical heterogeneity. If comparing groups, describe the direction of the effect. | Results: Pooled mean estimates of gonorrhea prevalence section; Tables 2-4; Tables S3, S4, and S5; Fig 2; Fig S1 and Fig S2 |
|  | 20c | Present results of all investigations of possible causes of heterogeneity among study results. | Results: Predictors of prevalence and sources of between-study heterogeneity section; Table 4; Tables S7 and S8 |
|  | 20d | Present results of all sensitivity analyses conducted to assess the robustness of the synthesised results. | Results: Predictors of prevalence and sources of between-study heterogeneity section; Table 5; Tables S7 and S8 |
| Reporting biases | 21 | Present assessments of risk of bias due to missing results (arising from reporting biases) for each synthesis assessed. | N/A |
| Certainty of evidence | 22 | Present assessments of certainty (or confidence) in the body of evidence for each outcome assessed. | N/A |
| **Discussion** | | |  |
| Discussion | 23a | Provide a general interpretation of the results in the context of other evidence. | Discussion section |
|  | 23b | Discuss any limitations of the evidence included in the review. | Discussion section |
|  | 23c | Discuss any limitations of the review processes used. | Discussion section |
|  | 23d | Discuss implications of the results for practice, policy, and future research. | Discussion section |
| **Other information** | | |  |
| Registration and  protocol | 24a | Provide registration information for the review, including register name and registration number, or state that the review was not registered. | N/A |
|  | 24b | Indicate where the review protocol can be accessed, or state that a protocol was not prepared. | N/A |
|  | 24c | Describe and explain any amendments to information provided at registration or in the protocol. | N/A |
| Support | 25 | Describe sources of financial or non-financial support for the review, and the role of the funders or sponsors in the review. | Funding and Acknowledgements |
| Competing interests | 26 | Declare any competing interests of review authors. | Declaration of interests |
| Availability of data, code, and other materials | 27 | Report which of the following are publicly available and where they can be found: template data collection forms; data extracted from included studies; data used for all analyses; analytic code; any other materials used in the review. | Data sharing statement |

Abbreviations: NA = Not applicable, p = page.

# **Table S2.** Data sources and search strategies for systematically reviewing *Neisseria gonorrhoeae* epidemiology in the Middle East and North Africa.

| **PubMed (Last searched: February 28, 2023)** |
| --- |
| "Neisseria gonorrhoeae"[Mesh] OR "Gonorrhea"[Mesh] OR "Pelvic Inflammatory Disease"[Mesh] OR "Epididymitis"[Mesh] OR "Orchitis"[Mesh] OR "Seminal vesicles"[Mesh] OR "Neisseria gonorrhoeae"[Text] OR "Gonorrhoeae"[Text] OR "Gonorrhea"[Text] OR "Gonococcus"[Text] OR "Gonococci"[Text] OR "Gonococcal"[Text] OR "Gonococcal infection"[Text] OR "Pelvic inflammatory disease"[Text] OR "Gonococcal epididymitis"[Text] OR "Orchi-epididymitis"[Text] OR "Orchiepididymitis"[Text] OR "seminal vesicle disease"[Text] OR "Seminal vesiculitis"[Text] AND ("Middle East"[Mesh] OR "Islam"[Mesh] OR "Arabs"[Mesh] OR "Arab World"[Mesh] OR "Africa, Northern"[Mesh] OR "Sudan"[Mesh] OR "Somalia"[Mesh] OR "Djibouti"[Mesh] OR "Pakistan"[Mesh] OR "South Sudan"[Mesh] OR "Middle East*"[Text] OR "Middle-East"[Text] OR "North Africa*"[Text] OR "North-Africa"[Text] OR "EMRO"[Text] OR "Eastern Mediterranean"[Text] OR "Arab*"[Text] OR "Arab World"[Text] OR "Islam*"[Text] OR "Afghanistan"[Text] OR "Afghan*"[Text] OR "Algeria*"[Text] OR "Bahrain*"[Text] OR "Djibouti"[Text] OR "Egypt*"[Text] OR "Jordan*"[Text] OR "Kuwait*"[Text] OR "Lebanon"[Text] OR "Leban*"[Text] OR "Libya*"[Text] OR "Iran*"[Text] OR "Iraq*"[Text] OR "Morocco"[Text] OR "Moroccan*"[Text] OR "Oman*"[Text] OR "Pakistan*"[Text] OR "Qatar*"[Text] OR "Saudi*"[Text] OR "Somalia"[Text] OR "Somal*"[Text] OR "Sudan*"[Text] OR "Syria*"[Text] OR "Tunisia*"[Text] OR "United Arab Emirates"[Text] OR "Emirat*"[Text] OR "West Bank"[Text] OR "Ghaza*"[Text] OR "Gaza*"[Text] OR "Palestine"[Text] OR "Palestinian*"[Text] OR "Yemen*"[Text] OR "UAE"[Text] OR "KSA"[Text] OR "Dubai"[Text] OR "Abu Dhabi"[Text] OR "Abu-Dhabi"[Text]) |
| **Embase (Last searched: February 28, 2023)** |
| exp gonorrhea / or exp neisseria gonorrhoeae / or exp epididymitis / or exp orchitis / or exp pelvic inflammatory disease/ or gonorrhea.mp. or neisseria gonorrhoeae.mp. or gonorrhoeae.mp. or gonococcus.mp. or gonococci.mp. or gonococcal.mp. or gonococcal infection.mp. or pelvic inflammatory disease.mp. or gonococcal epididymitis.mp. or orchi-epididymitis.mp. or orchiepididymitis.mp. or seminal vesicle disease.mp. or seminal disease.mp. or seminal vasculitis.mp. AND exp Middle East/ or exp North Africa/ or exp Arab/ or exp Afghanistan/ or exp Djibouti/ or exp Pakistan/ or exp Somalia/ or exp Sudan/ or exp South Sudan/ or (Middle East or North Africa or EMRO or Eastern Mediterranean or Arab or Arabs or Arab World or Islam or Afghanistan or Afghan* or Algeria* or Bahrain* or Djibouti or Egypt* or Jordan* or Kuwait* or Leban* or Libya* or Iran* or Iraq* or Morocc* or Oman* or Pakistan* or Qatar* or Saudi* or Somal* or Sudan* or Syria* or Tunisia* or United Arab Emirates or Emirat* or West Bank or Ghaza* or Gaza* or Palestin* or Yemen* or UAE or KSA or Dubai or Abu Dhabi or Sharjah).mp. |
| **National and regional databases (Last searched: February 28, 2023)** |
| **National and regional databases**   1. **Index Medicus for the Eastern Mediterranean Region**   gonorrhea OR Neisseria gonorrhoeae   1. **Iraqi Academic Scientific Journals Database**   gonorrhea OR Neisseria gonorrhoeae   1. **Scientific Information Database of Iran**   gonorrhea OR Neisseria gonorrhoeae   1. **PakMediNet of Pakistan**   gonorrhea OR Neisseria gonorrhoeae |

Abbreviations: MENA = Middle East and North Africa

# **Box S1.** Countries included in the Middle East and North Africa definition and their subregional classification.

- **Fertile Crescent:** Egypt, Iraq, Jordan, Lebanon, Palestine, Syria.
- **Gulf:** Bahrain, Kuwait, Oman, Qatar, Saudi Arabia, United Arab Emirates.
- **Horn of Africa:** Djibouti, Somalia, Sudan, Yemen
- **Maghreb:** Algeria, Libya, Morocco, Tunisia
- Afghanistan
- Iran
- Pakistan

**Box S2.** Variables extracted from relevant records meeting the inclusion criteria.

- Author(s)
- Year of publication
- Full citation
- Country
- City
- Study design
- Sampling methodology
- Year(s) of data collection
- Study site
- Study population
- Population characteristics (e.g., sex and age)
- Response rate
- Sample size of tested population
- Number of participants positive for *Neisseria gonorrhoeae* infection
- Reported *Neisseria gonorrhoeae* prevalence
- Anatomical site: urogenital (urethral, vaginal, endocervical, urine, and semen), anorectal, oropharyngeal, serum, and unclear
- Type of assay used for infection ascertainment: nucleic acid amplification test/polymerase chain reaction, culture, gram staining, wet mount, blood tested for antibodies, and unclear

# **Box S3.** Factors (variables) selected *a priori* and included in the univariable and multivariable meta-regression analyses.

| 1. Population type as defined in Box S3 2. Sex 3. Age groups classified to best fit reported data as:  - <25 years old - 25-34 years old - 35-44 years old - ≥45 years old - Mixed age bands  1. MENA subregion as defined in Box S1 2. National income as classified by the World Bank [3] 3. Assay type:  - NAAT/PCR - Culture - Gram stain - Wet mount - Other  1. Sample size:  - <200 - ≥200  1. Sampling method:  - Probability-based sampling - Non-probability-based sampling  1. Response rate:  - ≥80% - <80% - Unclear  1. Year of publication category:  - <2005 - 2005-2014 - ≥2015  1. Year of publication as a linear term 2. Year of data collection category^a^  - <2000 - 2000-2009 - ≥2010  1. Year of data collection as a linear term |
| --- |

Abbreviations: MENA = Middle East and North Africa, NAAT = nucleic acid amplification test, PCR = Polymerase chain reaction

^a^ The categories were set based on the observed median time between the year of publication and year of data collection of 4 years, approximated to 5 years to have 5-year brackets.

# **Table S3.** Studies reporting *Neisseria gonorrhoeae* prevalence in urogenital specimens in the Middle East and North Africa.

| **Author, year** | **Year(s) of data collection** | **Country** | **Site type^a^** | **Study design** | **Sampling** | **Specimen** | **Assay** | **Population characteristics** | **Sample size** | **NG prev (%)** |
| --- | --- | --- | --- | --- | --- | --- | --- | --- | --- | --- |
| **General populations** | | | | | | | | | | |
| Abdollahiyan, 2005 [4] | 2002-03 | Iran | Hospital | CS | Conv | Endocervical | Culture | Women of reproductive age | 1,951 | 0.6 |
| Abusarah, 2013 [5] | 2011 | Jordan | Dermato-venerology clinic | CC | Conv | Urine | NAAT/PCR | Urine samples tested from fertile men | 61 | 0.0 |
| Ahmadi, 2022 [6] | 2014-16 | Iran | Community | CS | Conv | Endocervical | NAAT/PCR | Women with normal delivery | 109 | 0.0 |
| Ahmadi, 2022 [6] | 2014-16 | Iran | Community | CS | Conv | Endocervical | NAAT/PCR | Fertile women | 100 | 0.0 |
| Ahmadnia, 2016 [7] | 2012-13 | Iran | Outpatient clinic | CS | MSCRS | Endocervical | Gram stain | Married women living in urban and rural areas | 4,274 | 0.6 |
| Al-Haddad, 2005 [8] | 2002 | Yemen | Antenatal clinic | CS | Conv | Urine | Culture | Pregnant women | 137 | 1.5 |
| Al-joubori, 2003 [9] | 2001 | Iraq | Hospital | CS | Conv | Vaginal | Gram stain | Healthy women | 292 | 8.2 |
| Al-joubori, 2003 [9] | 2001 | Iraq | Hospital | CS | Conv | Vaginal | Gram stain | Vaginal swabs from women attending a hospital | 57 | 5.3 |
| Al-joubori, 2003 [9] | 2001 | Iraq | Hospital | CS | Conv | Endocervical | Gram stain | Endocervical swabs from women | 73 | 12.3 |
| Al-joubori, 2003 [9] | 2001 | Iraq | Hospital | CS | Conv | Urethral | Gram stain | Urethral swabs from men attending a hospital | 40 | 30.0 |
| Al-joubori, 2003 [9] | 2001 | Iraq | Outpatient clinic | CS | Conv | Vaginal | Gram stain | High vaginal swabs from women | 74 | 16.2 |
| Al-joubori, 2003 [9] | 2001 | Iraq | Outpatient clinic | CS | Conv | Vaginal | Gram stain | Vaginal swabs from women attending a clinic | 30 | 13.3 |
| Al-joubori, 2003 [9] | 2001 | Iraq | Outpatient clinic | CS | Conv | Endocervical | Gram stain | Endocervical swabs from women | 22 | 27.3 |
| Al-joubori, 2003 [9] | 2001 | Iraq | Outpatient clinic | CS | Conv | Urethral | Gram stain | Urethral swabs from men attending a clinic | 30 | 40.0 |
| Al-Mousawi, 2006 [10] | 2002-03 | Iraq | OBGYN | CS | Conv | Vaginal | Culture | Pregnant women | 335 | 2.6 |
| Al-Muharmi, 2022 [11] | 202 | Oman | Hospital | CS | Conv | Endocervical | NAAT/PCR | Healthy women | 106 | 0.0 |
| Al-Muharmi, 2022 [11] | 202 | Oman | Hospital | CS | Conv | Urine | NAAT/PCR | Men getting a checkup | 45 | 2.2 |
| Al-Omar, 2005 [12] | 1999-00 | Iraq | OBGYN | CC | Conv | Vaginal | Gram stain | Healthy women | 20 | 0.0 |
| Al-Sweih, 2011 [13] | 2004 | Kuwait | Dermato-venerology clinic | CS | Conv | Vaginal | NAAT/PCR | Non-Kuwaiti women | 2,601 | 1.6 |
| Al-Sweih, 2011 [13] | 2004 | Kuwait | Outpatient clinic | CS | Conv | Vaginal | NAAT/PCR | Kuwaiti women | 5,938 | 1.4 |
| Alzahrani, 2010 [14] | 2005-06 | Saudi Arabia | Outpatient clinic | CS | RS | Endocervical | Culture | ANC attendees | 95 | 0.0 |
| Anwar, 2000 [15] | − | Pakistan | Community | CS | MSRS | Endocervical | Culture | Women with no vaginal discharge | 60 | 13.3 |
| Anwer, 2001 [16] | − | Pakistan | Community | CS | Conv | Endocervical | Gram stain | Married women | 100 | 0.0 |
| As'ad, 2004 [17] | − | Jordan | Outpatient clinic | CS | Conv | Vaginal | NAAT/PCR | Asymptomatic women | 144 | 0.0 |
| Awad, 2013 [18] | − | Saudi Arabia | Outpatient clinic | CS | Conv | Urine | NAAT/PCR | Women undergoing Pregnancy tests | 144 | 2.1 |
| Azizmohammadi, 2016 [19] | 2015 | Iran | Outpatient clinic | CC | Conv | Vaginal | Culture | Fertile women with no history of infertility | 190 | 5.3 |
| Baghchesaraei, 2011 [20] | 2009 | Iran | Unclear | CS | Conv | Vaginal | Culture | Gynecology clinic attendees | 328 | 0.9 |
| Bellaji, 2017 [21] | 2013-14 | Morocco | Outpatient clinic | CS | Conv | Urogenital | NAAT/PCR | Women attending a family planning clinic | 537 | 0.4 |
| Chaudry, 2021 [22] | 2019 | Pakistan | Hospital | CS | Conv | Vaginal | NAAT/PCR | Pregnant women | 1,001 | 0.1 |
| Deeb, 2003 [23] | 1998 | Lebanon | STI clinic | CS | MSRS | Endocervical | Gram stain | Ever married women | 506 | 0.0 |
| Dezfulimanesh, 2005 [24] | − | Iran | OBGYN | CC | Conv | Endocervical | Culture | Non-pregnant women | 250 | 0.4 |
| Dezfulimanesh, 2005 [24] | − | Iran | Prison | CC | Conv | Endocervical | Culture | Pregnant women | 250 | 0.4 |
| El-Sayed, 2002 [25] | 1999-00 | Egypt | STI clinic | CS | Conv | Urine | NAAT/PCR | FPC attendees | 108 | 2.8 |
| El-Sayed, 2002 [25] | 1999-00 | Egypt | OBGYN | CS | Conv | Urine | NAAT/PCR | ANC attendees | 604 | 2.0 |
| El-SayedAbdou, 2018 [26] | 2014-15 | Egypt | OBGYN | CS | RS | Endocervical | NAAT/PCR | Women removing their IUDs | 40 | 30.0 |
| Esteghamati, 2020 [27] | 2017-18 | Iran | Hospital | CS | Conv | Endocervical | NAAT/PCR | Women with normal delivery | 125 | 1.6 |
| Farhan, 2022 [28] | 2021 | Iraq | Hospital | CS | RS | Vaginal | Gram stain | Healthy women | 160 | 0.6 |
| Hamzeh, 2016 [29] | 2010 | Lebanon | Hospital | CS | Conv | Urogenital | NAAT/PCR | Patients attending a hospital in 2010 | 51 | 0.0 |
| Hamzeh, 2016 [29] | 2011 | Lebanon | Hospital | CS | Conv | Urogenital | NAAT/PCR | Patients attending a hospital in 2011 | 97 | 1.0 |
| Hamzeh, 2016 [29] | 2012 | Lebanon | Hospital | CS | Conv | Urogenital | NAAT/PCR | Patients attending a hospital in 2012 | 92 | 1.0 |
| Hamzeh, 2016 [29] | 2013 | Lebanon | Hospital | CS | Conv | Urogenital | NAAT/PCR | Patients attending a hospital in 2013 | 55 | 2.5 |
| Hanna, 2020 [30] | 2016-17 | Lebanon | OBGYN | CS | Conv | Urogenital | NAAT/PCR | Women visiting OBGYN for regular checkup | 505 | 0.2 |
| Hassan, 2005 [31] | 2002 | Iraq | Hospital | CC | Conv | Vaginal | Culture | Pregnant women | 107 | 0.0 |
| Hassanzadeh, 2013 [32] | 2009-11 | Iran | Outpatient clinic | CS | Conv | Endocervical | NAAT/PCR | Pregnant women tested by NAAT/PCR | 1,100 | 1.2 |
| Hassanzadeh, 2013 [32] | 2009-11 | Iran | Outpatient clinic | CS | Conv | Endocervical | Culture | Pregnant women tested by culture | 1,100 | 0.0 |
| Ismail, 1990a [33] | 1987 | Somalia | STI clinic | CS | Conv | Urogenital | Culture | Healthy women | 194 | 0.0 |
| Ismail, 1990 [33] | 1987 | Somalia | STI clinic | CS | Conv | Urethral | Culture | Healthy men | 187 | 0.0 |
| Kafi, 2000 [34] | − | Sudan | Antenatal clinic | CS | MSRS | Vaginal | Gram stain | Healthy women tested by Gram stain | 338 | 1.2 |
| Kafi, 2000 [34] | − | Sudan | Hospital | CS | MSRS | Vaginal | Culture | Healthy women tested by culture | 338 | 0.9 |
| Karim, 2018 [35] | 2013-15 | Morocco | OBGYN | CS | Conv | Endocervical | NAAT/PCR | Women attending the OBGYN clinic | 1,053 | 14.1 |
| Karim, 2021 [36] | 2015-17 | Morocco | OBGYN | CS | Conv | Endocervical | NAAT/PCR | Women attending the OBGYN clinic | 809 | 14.2 |
| Khalil, 2012 [37] | 2010-11 | Iraq | Hospital | CS | Conv | Vaginal | Gram stain | Women attending a FPC tested with direct smear | 250 | 1.6 |
| Khalil, 2012 [37] | 2010-11 | Iraq | Hospital | CS | Conv | Vaginal | Culture | Women attending a FPC tested with culture | 250 | 4.4 |
| Khoder, 2019 [38] | 2014-16 | Lebanon | Hospital | CS | Conv | Semen | NAAT/PCR | Men with normal seminograms | 139 | 12.2 |
| Latif, 2020 [39] | 2018 | Iraq | Outpatient clinic | CS | Conv | Endocervical | NAAT/PCR | Women attending gynecological clinics | 25 | 1.0 |
| Lau, 2021 [40] | 2020 | Oman | Outpatient clinic | CS | Conv | Urine | NAAT/PCR | Pregnant women | 221 | 0.0 |
| Mahafzah, 2008 [41] | 2003-05 | Jordan | STI clinic | CS | Conv | Endocervical | NAAT/PCR | FPC attendees | 186 | 2.2 |
| Mir, 2009 [42] | 2007 | Pakistan | Community | CS | MSRS | Urine | NAAT/PCR | Men 16-45 in 6 major cities of Pakistan | 2,383 | 0.1 |
| MOH - Iran, 2008 [43] | 2003 | Iran | Unclear | CS | Conv | Endocervical | Culture | FPC attendees | 1,951 | 0.6 |
| MOH - Iran, 2008 [43] | 1994 | Iran | Prison | CS | Conv | Endocervical | Culture | Women attending gynecology clinics | 500 | 0.4 |
| MOH - Jordan, 2004 [44] | 2003 | Jordan | Outpatient clinic | CS | Conv | Endocervical | NAAT/PCR | Asymptomatic women | 213 | 0.5 |
| MOH - Morocco, 2001 [45] | 1999 | Morocco | Community | CS | Conv | Urine | NAAT/PCR | Pregnant women | 323 | 0.7 |
| MOH - Morocco, 2001 [45] | 1999 | Morocco | Community | CS | Conv | Urine | NAAT/PCR | FPC attendees | 518 | 1.4 |
| Mortazavi, 2021 [46] | 2019-20 | Iran | Outpatient clinic | CS | Conv | Endocervical | NAAT/PCR | Healthy women | 162 | 3.1 |
| Motamedifar, 2020 [47] | 2015 | Iran | Hospital | CC | Conv | Semen | NAAT/PCR | Healthy men | 150 | 0.7 |
| Nateghi Rostami, 2017[48] | 2013-14 | Iran | Hospital | CS | Conv | Urogenital | NAAT/PCR | Asymptomatic women tested with PCR | 143 | 5.6 |
| Nateghi Rostami, 2017 [48] | 2013-14 | Iran | Hospital | CS | Conv | Urogenital | Culture | Asymptomatic women tested with culture | 143 | 2.1 |
| Ortashi, 2004 [49] | 1999 | Sudan | Prison | CS | Conv | Urogenital | Culture | Pregnant women | 151 | 2.0 |
| Pourabbas, 2018 [50] | − | Iran | Hospital | CS | Conv | Endocervical | NAAT/PCR | Women with vaginal delivery | 239 | 1.3 |
| Rashidi, 2009 [51] | − | Iran | Prison | CC | Conv | Urine | NAAT/PCR | Pregnant women | 170 | 0.0 |
| Sameni, 2022 [52] | 2018-19 | Iran | OBGYN | CS | Conv | Vaginal | NAAT/PCR | Pregnant women | 54 | 1.8 |
| Shaaban, 1994 [53] | − | Egypt | Outpatient clinic | CC | Conv | Endocervical | Culture | Healthy women | 50 | 2.0 |
| WHO, 2005a [54] | − | Somalia | Outpatient clinic | CS | Conv | Urogenital | NAAT/PCR | ANC attendees from an outpatient clinic | 4,732 | 0.8 |
| WHO, 2005a [54] | − | Somalia | Hospital | CS | Conv | Urogenital | NAAT/PCR | ANC attendees from a hospital | 509 | 0.4 |
| **Intermediate risk populations** | | | | | | | | | | |
| Abdelrahim, 2017 [55] | − | Sudan | Outpatient clinic | CS | Conv | Endocervical | Culture | ANC attendees in displaced camps and women in prison | 350 | 0.0 |
| Altaf, 2009 [56] | 2002 | Pakistan | Outpatient clinic | CS | Conv | Urine | NAAT/PCR | Juvenile prison inmates | 321 | 1.0 |
| El-Sayed, 2002 [25] | 1999-00 | Egypt | OBGYN | CS | Conv | Urine | NAAT/PCR | PWID | 150 | 2.7 |
| Faisel, 2006 [57] | − | Pakistan | Community | CS | MSCRS | Urine | NAAT/PCR | Migrant men in Lahore | 195 | 0.5 |
| Ghanbarzadeh, 2006 [58] | − | Iran | Outpatient clinic | CS | Conv | Vaginal | Gram stain | Female prisoners | 199 | 4.5 |
| Mousaviani, 2004 [59] | − | Iran | Outpatient clinic | CS | Conv | Vaginal | Culture | Female prisoners | 150 | 0.0 |
| Platt, 2009 [60] | 2007 | Pakistan | Community | CS | RDS | Urine | NAAT/PCR | PWID in Rawalpindi | 301 | 1.3 |
| Platt, 2009 [60] | 2007 | Pakistan | Community | CS | RDS | Urine | NAAT/PCR | PWID in Abbottabad | 100 | 0.0 |
| Rehan, 2009 [61] | 2004 | Pakistan | Community | CS | TLS | Urethral | NAAT/PCR | PWID in Lahore | 397 | 1.0 |
| Rehan, 2009 [61] | 2004 | Pakistan | Community | CS | TLS | Urethral | NAAT/PCR | PWID in Karachi | 393 | 1.8 |
| Rehan, 2009 [61] | 2004 | Pakistan | Community | CS | TLS | Urethral | NAAT/PCR | Truck drivers in Lahore | 397 | 0.8 |
| Rehan, 2009 [61] | 2004 | Pakistan | Community | CS | TLS | Urethral | NAAT/PCR | Truck drivers in Karachi | 397 | 3.5 |
| Shahcheraghi, 2010 [62] | 2008 | Iran | Antenatal clinic | CS | Conv | Vaginal | NAAT/PCR | Female prisoners | 500 | 0.0 |
| Valadkhani, 2010 [63] | − | Iran | OBGYN | CS | Conv | Vaginal | Culture | Female prisoners | 377 | 0.0 |
| **Female sex workers** | | | | | | | | | | |
| Bellaji, 2017 [21] | 2013-14 | Morocco | Community | CS | Conv | Urogenital | NAAT/PCR | FSWs | 519 | 9.4 |
| El-Sayed, 2002 [25] | 1999-00 | Egypt | OBGYN | CS | Conv | Urine | NAAT/PCR | FSWs | 52 | 7.7 |
| Hawkes, 2009 [64] | 2007 | Pakistan | Community | CS | RDS | Endocervical | NAAT/PCR | FSWs in Rawalpindi | 426 | 2.0 |
| Hawkes, 2009 [64] | 2007 | Pakistan | Community | CS | RDS | Endocervical | NAAT/PCR | FSWs in Abbottabad | 107 | 1.9 |
| Kazerooni, 2013 [65] | 2010 | Iran | Outpatient clinic | CS | RDS | Vaginal | Culture | FSWs | 278 | 1.4 |
| Khan, 2011 [66] | 2007 | Pakistan | Community | CS | RDS | Endocervical | NAAT/PCR | FSWs in Lahore | 730 | 7.5 |
| Khezri, 2020 [67] | 2015 | Iran | Community | CS | Conv | Vaginal | NAAT/PCR | FSWs | 1,296 | 1.3 |
| Mirzazadeh, 2020 [68] | 2015 | Iran | Outpatient clinic | CS | Conv | Vaginal | NAAT/PCR | FSWs visiting harm reduction clinics | 1,337 | 1.3 |
| MOH - Morocco, 2008 [69] | 2007 | Morocco | Outpatient clinic | CS | Conv | Urogenital | NAAT/PCR | FSWs | 141 | 10.6 |
| MOH - Morocco, 2011 [70] | 2011 | Morocco | Outpatient clinic | CS | RDS | Endocervical | NAAT/PCR | FSWs in Agadir | 368 | 11.7 |
| Nasirian, 2017 [71] | 2013-14 | Iran | Outpatient clinic | CS | Conv | Endocervical | NAAT/PCR | FSWs | 99 | 9.1 |
| Rehan, 2009 [61] | 2004 | Pakistan | Community | CS | MSRS | Endocervical | NAAT/PCR | FSWs in Lahore | 383 | 12.3 |
| Rehan, 2009 [61] | 2004 | Pakistan | STI clinic | CS | Snowball | Endocervical | NAAT/PCR | FSWs in Karachi | 348 | 9.8 |
| Zirak-Zadah, 1977 [72] | 1972 | Iran | Outpatient clinic | CS | Conv | Vaginal | Gram stain | FSWs | 921 | 12.1 |
| Znazen, 2010 [73] | 2007 | Tunisia | Outpatient clinic | CS | Conv | Endocervical | NAAT/PCR | FSWs tested using NAAT/PCR | 188 | 11.2 |
| Znazen, 2010 [73] | 2007 | Tunisia | Outpatient clinic | CS | Conv | Endocervical | Culture | FSWs tested using culture | 188 | 3.7 |
| **Male sex workers and men who have sex with men** | | | | | | | | | | |
| El-Sayed, 2002 [25] | 1999-00 | Egypt | OBGYN | CS | Conv | Urine | NAAT/PCR | MSM | 80 | 8.8 |
| Hawkes, 2009 [64] | 2007 | Pakistan | Community | CS | RDS | Urine | NAAT/PCR | MSWs in Rawalpindi (bantha) | 195 | 0.0 |
| Hawkes, 2009 [64] | 2007 | Pakistan | Community | CS | RDS | Urine | NAAT/PCR | MSWs in Rawalpindi (khotki) | 364 | 0.0 |
| Hawkes, 2009 [64] | 2007 | Pakistan | Community | CS | RDS | Urine | NAAT/PCR | MSWs in Rawalpindi (khusra) | 253 | 0.0 |
| Hawkes, 2009 [64] | 2007 | Pakistan | STI clinic | CS | RDS | Urine | NAAT/PCR | MSWs in Abbottabad (bantha) | 83 | 0.0 |
| Hawkes, 2009 [64] | 2007 | Pakistan | Community | CS | RDS | Urine | NAAT/PCR | MSWs in Abbottabad (khotki & khusra) | 20 | 0.0 |
| MOH - Morocco, 2015 [74] | 2015 | Morocco | Outpatient clinic | CS | RDS | Urine | NAAT/PCR | MSM in Agadir | 247 | 1.3 |
| MOH - Morocco, 2015 [74] | 2015 | Morocco | Antenatal clinic | CS | RDS | Urine | NAAT/PCR | MSM in Marrakech | 252 | 3.8 |
| Rehan, 2009 [61] | 2004 | Pakistan | Community | CS | MSCRS | Urethral | NAAT/PCR | Hijra in Lahore | 198 | 4.0 |
| Rehan, 2009 [61] | 2004 | Pakistan | Community | CS | MSCRS | Urethral | NAAT/PCR | Hijra in Karachi | 197 | 3.0 |
| Rehan, 2009 [61] | 2004 | Pakistan | Community | CS | RDS | Urethral | NAAT/PCR | MSWs in Lahore | 395 | 3.3 |
| Rehan, 2009 [61] | 2004 | Pakistan | Hospital | CS | Snowball | Urethral | NAAT/PCR | MSWs in Karachi | 396 | 5.8 |
| **Symptomatic women** | | | | | | | | | | |
| Afrakhteh, 2013 [75] | 2005-08 | Iran | Outpatient clinic | CS | Conv | Urine | NAAT/PCR | ≥15 years old symptomatic women | 49 | 12.2 |
| Afrasiabi, 2014 [76] | 2012-13 | Iran | Outpatient clinic | CS | Conv | Endocervical | Culture | Gynecology clinic attendees | 294 | 2.4 |
| Akya, 2013 [77] | 2011 | Iran | OBGYN | CC | Conv | Endocervical | NAAT/PCR | Women with cervicitis | 255 | 2.4 |
| Al Habib, 2008 [78] | 1997-99 | Iraq | Hospital | CS | Conv | Vaginal | Gram stain | Women with vaginal discharge | 440 | 3.0 |
| Al Kaisi, 2006 [79] | 1992 | Iraq | Antenatal clinic | CS | Conv | Endocervical | Gram stain | Women with vaginal discharge | 480 | 1.7 |
| Al Quaiz, 2000 [80] | 1998 | Saudi Arabia | Outpatient clinic | CS | Conv | Urogenital | Gram stain | Women with vaginal discharge | 175 | 0.6 |
| Ali, 2018 [81] | 2017 | Iraq | Outpatient clinic | CS | Conv | Endocervical | Gram stain | Women with vaginal discharge tested by Gram stain | 100 | 20.0 |
| Ali, 2018 [81] | 2017 | Iraq | Outpatient clinic | CS | Conv | Endocervical | Culture | Women with vaginal discharge tested by culture | 100 | 25.0 |
| Ali, 2018 [81] | 2017 | Iraq | Outpatient clinic | CS | Conv | Endocervical | NAAT/PCR | Women with vaginal discharge tested using PCR | 100 | 30.0 |
| Al-Muharmi, 2022 [11] | 202 | Oman | Hospital | CS | Conv | Endocervical | NAAT/PCR | Symptomatic women | 137 | 1.5 |
| Al-Muqdadi, 2010 [82] | 1997-98 | Iraq | Hospital | CS | Conv | Vaginal | Wet mount | Women complaining of vaginal infections | 320 | 1.3 |
| Al-Mutairi, 2007 [83] | 2003 | Kuwait | Outpatient clinic | CS | Conv | Urethral | Culture | Symptomatic women | 28 | 0.0 |
| Al-Omar, 2005 [12] | 1999-00 | Iraq | OBGYN | CC | Conv | Vaginal | Gram stain | Women with vaginal discharge and vaginal discomfort | 80 | 2.5 |
| Alzahrani, 2010 [14] | 2005-06 | Saudi Arabia | Hospital | CS | RS | Endocervical | Culture | Women with lower genital tract infection | 102 | 7.8 |
| Anwar, 2000 [15] | − | Pakistan | Community | CS | MSRS | Endocervical | Culture | Women with vaginal discharge | 100 | 10.0 |
| Arif, 1989 [84] | − | Egypt | Outpatient clinic | CS | Conv | Endocervical | Culture | Women with urethritis tested by culture | 46 | 19.5 |
| Arif, 1989 [84] | − | Egypt | Outpatient clinic | CS | Conv | Endocervical | Gram stain | Women with urethritis tested by Gram stain | 46 | 10.8 |
| Arif, 1989 [84] | − | Egypt | Outpatient clinic | CS | Conv | Endocervical | EIA | Women with urethritis tested by GC-EIA | 46 | 26.0 |
| As'ad, 2004 [17] | − | Jordan | Outpatient clinic | CS | Conv | Vaginal | NAAT/PCR | Women with vaginal discharge | 641 | 1.2 |
| Azizi, 2017 [85] | 2012-14 | Afghanistan | Outpatient clinic | CS | Conv | Vaginal | Culture | Women with suspected genital tract infection | 1,297 | 0.6 |
| Bakhshi, 2019 [86] | − | Iran | OBGYN | CS | Conv | Vaginal | NAAT/PCR | Women with bacterial vaginosis | 16 | 6.3 |
| Bakhtiari, 2007 [87] | 2003-04 | Iran | Outpatient clinic | CS | Conv | Endocervical | Culture | Women with gynecological complaints | 550 | 0.2 |
| Douaa Hamza, 2013 [88] | 2012-13 | Iraq | Hospital | CS | Conv | Vaginal | Culture | Women with vaginitis | 90 | 2.2 |
| Ekhlas Mushref, 2010 [89] | 2005-06 | Iraq | OBGYN | CS | Conv | Vaginal | Gram stain | Women with vaginal discharge | 250 | 2.8 |
| Elkayal, 2015 [90] | − | Egypt | Prison | CS | Conv | Endocervical | Culture | Gynecology clinic attendees tested by culture | 50 | 2.0 |
| Elkayal, 2015 [90] | − | Egypt | Outpatient clinic | CS | Conv | Endocervical | NAAT/PCR | Gynecology clinic attendees tested by NAAT/PCR | 50 | 2.0 |
| Faroughi, 2021 [91] | 2014 | Iran | Hospital | CS | Conv | Vaginal | NAAT/PCR | Infertile women with vaginal symptoms | 60 | 6.6 |
| Farraj, 2010 [92] | − | Palestine | OBGYN | CS | RS | Endocervical | NAAT/PCR | Women with endocervical abnormalities | 213 | 1.4 |
| Ghobti, 2007 [93] | 2005-06 | Iran | Outpatient clinic | CS | Conv | Vaginal | Culture | Women with vaginal discharge | 1,252 | 1.0 |
| Gul, 2005 [94] | − | Pakistan | Community | CS | MSRS | Endocervical | Unclear | Symptomatic pregnant women | 400 | 0.0 |
| Haddadian, 2011 [95] | 2010-11 | Iran | OBGYN | CS | Conv | Endocervical | NAAT/PCR | Women with cervicitis | 238 | 0.0 |
| Jaafar, 2008 [96] | 2005-06 | Iraq | Hospital | CS | Conv | Endocervical | Culture | Women suffering from cervicitis | 450 | 6.2 |
| Kadir, 1989 [97] | 1986 | Iraq | Hospital | CS | Conv | Vaginal | Culture | Women with vaginal discharge | 646 | 0.3 |
| Kareem, 2020 [98] | 2009-20 | Iraq | Outpatient clinic | CS | Conv | Vaginal | Culture | Diabetic women with VD | 110 | 10.0 |
| Kazemian, 2022 [99] | 2019 | Iran | OBGYN | CS | Conv | Urogenital | NAAT/PCR | Women with vaginitis | 169 | 6.5 |
| Luni, 2005 [100] | − | Pakistan | OBGYN | CS | Conv | Vaginal | Culture | Women complaining of vaginal symptoms | 304 | 0.3 |
| Mahafzah, 2008 [41] | 2003-05 | Jordan | Outpatient clinic | CS | Conv | Endocervical | NAAT/PCR | Symptomatic women | 1,089 | 0.9 |
| Mahdi, 1998 [101] | 1994-95 | Iraq | Hospital | CC | Conv | Vaginal | Culture | Symptomatic pregnant women | 119 | 4.2 |
| Mehrabani, 2014 [102] | 2010 | UAE | Outpatient clinic | CS | Conv | Endocervical | NAAT/PCR | Symptomatic women | 201 | 5.5 |
| Moaiedmohseni, 2012 [103] | − | Iran | Antenatal clinic | CS | Conv | Vaginal | Gram stain | Symptomatic pregnant women | 110 | 0.0 |
| MOH - Jordan, 2004 [44] | 2003 | Jordan | Outpatient clinic | CS | Conv | Endocervical | NAAT/PCR | Women with vaginal discharge | 991 | 0.7 |
| MOH - Morocco, 2008 [69] | 2007 | Morocco | Outpatient clinic | CS | Conv | Urogenital | NAAT/PCR | Women with pelvic pain | 986 | 1.3 |
| Mohseni, 2013 [104] | − | Iran | STI clinic | CS | Conv | Vaginal | NAAT/PCR | Women referred for testing | 44 | 4.5 |
| Molaei, 2017 [105] | 2013-14 | Iran | Hospital | CS | Conv | Endocervical | Culture | Women with vaginal discharge | 100 | 4.0 |
| Mozher, 2011 [106] | 2008 | Iraq | Outpatient clinic | CS | Conv | Vaginal | Culture | Symptomatic women of reproductive age | 150 | 2.7 |
| Nateghi Rostami, 2017 [48] | 2013-14 | Iran | Hospital | CS | Conv | Urogenital | NAAT/PCR | Symptomatic women tested with culture | 277 | 3.2 |
| Nateghi Rostami, 2017 [48] | 2013-14 | Iran | Hospital | CS | Conv | Urogenital | Culture | Symptomatic women tested with PCR | 277 | 0.7 |
| Omer, 1980 [107] | 1976-78 | Sudan | Community | CS | Conv | Urogenital | Culture | Women with vaginal discharge | 132 | 3.8 |
| Omer, 1985 [108] | − | Sudan | Outpatient clinic | CS | Conv | Endocervical | Culture | Women with vaginal discharge | 822 | 1.5 |
| Rahimi, 2011 [109] | − | Iran | Hospital | CS | Conv | Endocervical | Gram stain | Symptomatic women tested by Gram stain | 126 | 38.1 |
| Rahimi, 2011 [109] | − | Iran | Hospital | CS | Conv | Endocervical | Culture | Symptomatic women tested by culture | 126 | 9.5 |
| Rjabpour, 2020 [110] | 2017-18 | Iran | Outpatient clinic | CS | Conv | Endocervical | NAAT/PCR | Women with genitourinary symptoms | 180 | 7.2 |
| Ryan, 1998 [111] | 1995-96 | Morocco | Antenatal clinic | CS | Conv | Urine | NAAT/PCR | Women with vaginal discharge attending an ANC | 704 | 5.4 |
| Ryan, 1998 [111] | 1995-96 | Morocco | Outpatient clinic | CS | Conv | Urine | NAAT/PCR | Women with vaginal discharge attending a clinic | 375 | 3.2 |
| Ryan, 1998 [111] | 1995-96 | Morocco | STI clinic | CS | Conv | Urine | NAAT/PCR | Women with vaginal discharge attending an STI clinic | 40 | 10.0 |
| Sadiq & Yousif, 2008 [112] | 2005-07 | Iraq | Hospital | CS | Conv | Endocervical | Culture | Women with bacterial vaginosis | 300 | 16.0 |
| Saleh, 2012 [113] | 2009-10 | Kuwait | Outpatient clinic | CS | Conv | Endocervical | NAAT/PCR | Women with vaginal discharge | 607 | 0.3 |
| Sallam, 1982 [114] | 1979 | Egypt | Outpatient clinic | CS | RS | Endocervical | Culture | Endocervical swabs of women with vaginal discharge | 200 | 7.0 |
| Sami, 2005 [115] | 2004 | Pakistan | Community | CS | Conv | Urogenital | Gram stain | Symptomatic women | 221 | 1.4 |
| Shakibaei, 2008 [116] | 2004-06 | Iran | Outpatient clinic | CS | Conv | Urogenital | Culture | Urethritis and cervicitis patients | 205 | 18.0 |
| Tabasi, 2002 [117] | 2001 | Iran | Outpatient clinic | CS | Conv | Endocervical | Culture | Women with cervicitis | 315 | 0.6 |
| Torabizadeh, 2016 [118] | 2013-14 | Iran | STI clinic | CS | Conv | Vaginal | NAAT/PCR | Women with UTI | 150 | 2.0 |
| Torabizadeh, 2016 [118] | 2013-14 | Iran | Outpatient clinic | CS | Conv | Vaginal | NAAT/PCR | Women with genital problems referred for curettage | 100 | 2.0 |
| Yaseen, 2020 [119] | 2008 | Iraq | Outpatient clinic | CS | Conv | Endocervical | Wet mount | Women with cervicitis | 67 | 7.5 |
| Znazen, 2013 [120] | − | Tunisia | OBGYN | CS | Conv | Urogenital | NAAT/PCR | Symptomatic women requesting abortion | 220 | 5.0 |
| Zolfaghari, 2022 [121] | 2018-20 | Iran | Hospital | CS | Conv | Endocervical | Culture | Symptomatic women | 468 | 7.7 |
| Zribi, 2008 [122] | 2003-04 | Tunisia | OBGYN | CS | Conv | Urogenital | NAAT/PCR | Women with vaginal discharge | 116 | 1.0 |
| **Symptomatic men** | | | | | | | | | | |
| Akhi, 2009 [123] | 2003-04 | Iran | Hospital | CS | Conv | Urethral | Unclear | Men with urethritis | 200 | 28.0 |
| Alami, 2002 [124] | 2001 | Morocco | Community | CS | MSRS | Urine | NAAT/PCR | Men with urethral discharge | 399 | 52.4 |
| Alavi, 2009 [125] | 2005-07 | Iran | OBGYN | CS | Conv | Urethral | Culture | Men with urethritis | 44 | 36.5 |
| Al-Hattawi, 1996 [126] | 1992-93 | UAE | Hospital | CS | Conv | Urogenital | Gram stain | Men with urethritis | 79 | 45.6 |
| Ali Miknas, 2008 [127] | 2002-04 | Iraq | STI clinic | CS | Conv | Urine | Unclear | Men with prostatic pain | 163 | 0.6 |
| Al-Jawamis, 1991 [128] | − | Jordan | Outpatient clinic | CS | Conv | Urethral | Culture | Men suffering from urethral discharge | 72 | 55.6 |
| Al-Muharmi, 2022 [11] | 202 | Oman | Hospital | CS | Conv | Urine | NAAT/PCR | Symptomatic men | 44 | 11.4 |
| Al-Mutairi, 2007 [83] | 2003-04 | Kuwait | Outpatient clinic | CS | Conv | Urethral | Culture | Symptomatic men | 1,068 | 36.2 |
| Al-Sweih, 2011 [129] | − | Kuwait | Community | CS | Conv | Urogenital | NAAT/PCR | Non-Kuwaiti men with urethritis | 205 | 33.2 |
| Al-Sweih, 2011 [129] | − | Kuwait | Community | CS | Conv | Urogenital | NAAT/PCR | Kuwaiti men with urethritis | 221 | 34.8 |
| Amin, 2007 [130] | − | Egypt | Outpatient clinic | CS | Conv | Urogenital | NAAT/PCR | Men suffering from urethritis | 30 | 40.0 |
| Arif, 1989 [84] | − | Egypt | Outpatient clinic | CS | Conv | Urethral | Culture | Men with urethritis tested by culture | 97 | 24.7 |
| Arif, 1989 [84] | − | Egypt | Outpatient clinic | CS | Conv | Urethral | Gram stain | Men with urethritis tested by Gram stain | 97 | 19.5 |
| Arif, 1989 [84] | − | Egypt | Outpatient clinic | CS | Conv | Urethral | EIA | Men with urethritis tested by EIA | 97 | 26.8 |
| Aziz, 1991 [131] | 1983-90 | Bahrain | Outpatient clinic | CS | Conv | Urethral | Gram stain | Men suffering from urethritis between 1983-1990 | 7,033 | 45.0 |
| Baaj, 1980 [132] | 1965-77 | Morocco | Outpatient clinic | CS | Conv | Urethral | Gram stain | Men with acute urethritis | 1,468 | 57.7 |
| Devrajani, 2010 [133] | 2007-08 | Pakistan | STI clinic | CS | Conv | Urogenital | Gram stain | Patients with history of discharge | 256 | 56.0 |
| El Gamal, 1988 [134] | − | Saudi Arabia | Outpatient clinic | CS | Conv | Urethral | Gram stain | Symptomatic men tested by Gram stain | 48 | 96.0 |
| El Gamal, 1988 [134] | − | Saudi Arabia | Outpatient clinic | CS | Conv | Urethral | Culture | Symptomatic men tested by culture | 48 | 85.0 |
| El-Ghazzawy, 1993 [135] | − | Egypt | Community | CS | Conv | Urine | Culture | Symptomatic men | 50 | 2.0 |
| Elghoul, 1990 [136] | 1988 | Libya | STI clinic | CS | Conv | Urethral | Culture | Men with urethral discharge | 64 | 65.6 |
| Ezz Eddin, 1992 [137] | − | Egypt | Outpatient clinic | CS | Conv | Urethral | Culture | Symptomatic men tested by culture | 59 | 20.3 |
| Ezz Eddin, 1992 [137] | − | Egypt | Antenatal clinic | CS | Conv | Urine | Gram stain | Symptomatic men tested by Gram stain microscopy | 59 | 20.3 |
| Farook, 2007 [138] | 2002-03 | Pakistan | Hospital | CS | Conv | Urethral | Culture | Male with urethral discharge tested using Gram stain | 60 | 36.7 |
| Farook, 2007 [138] | 2002-03 | Pakistan | Hospital | CS | Conv | Urethral | Culture | Male with urethral discharge tested using culture | 60 | 43.0 |
| Fath Elahzadeh, 2004 [139] | − | Iran | Outpatient clinic | CS | Conv | Urethral | Culture | Symptomatic men | 67 | 37.3 |
| Haberberger, 1989 [140] | 1989 | Djibouti | Antenatal clinic | CS | Conv | Urethral | Culture | Men with urethral discharge tested using culture | 24 | 8.3 |
| Haberberger, 1989 [140] | 1989 | Djibouti | Outpatient clinic | CS | Conv | Urethral | Gram stain | Men with urethral discharge tested using Gram stain | 24 | 12.5 |
| Haberberger, 1989 [140] | 1988 | Djibouti | Outpatient clinic | CS | Conv | Urethral | Culture | Men with urethral discharge in 1988 | 105 | 32.4 |
| Hancali, 2013 [141] | 2009 | Morocco | Outpatient clinic | CS | Conv | Urethral | Culture | Men with urethritis tested using culture | 171 | 48.0 |
| Hancali, 2013 [141] | 2009 | Morocco | Outpatient clinic | CS | Conv | Urethral | NAAT/PCR | Men with urethritis tested using PCR | 171 | 63.0 |
| Heidari, 2012 [142] | 2004-09 | Iran | Hospital | CS | Conv | Urine | Culture | Men with epididymo-orchitis | 83 | 4.8 |
| Isam yousif, 2014 [143] | 2010-11 | Iraq | Outpatient clinic | CS | Conv | Urethral | Gram stain | Men with urethritis | 312 | 9.0 |
| Ismail, 1990 [144] | 1986 | Somalia | STI clinic | CC | Conv | Urethral | Culture | Men with urethral discharge | 47 | 48.9 |
| Mahgoub, 2005 [145] | 2000-01 | Sudan | STI clinic | CS | Conv | Urethral | Gram stain | Men with urethral discharge | 40 | 10.0 |
| Massenet, 1999 [146] | 1996-97 | Djibouti | Outpatient clinic | CS | Conv | Urethral | Gram stain | Men with urethral discharge tested with Gram stain | 200 | 96.0 |
| Massenet, 1999 [146] | 1996-97 | Djibouti | Outpatient clinic | CS | Conv | Urethral | Culture | Men with urethral discharge tested with culture | 200 | 94.0 |
| Mohammed, 2007 [147] | 2001-02 | Iraq | Outpatient clinic | CS | Conv | Urethral | Culture | Men with urethral discharge | 52 | 44.2 |
| Naderinasab, 2009 [148] | 2003-04 | Iran | STI clinic | CS | Conv | Urethral | Gram stain | Men with urethritis tested by Gram stain microscopy | 1,384 | 5.8 |
| Naderinasab, 2009 [148] | 2003-04 | Iran | Outpatient clinic | CS | Conv | Urethral | Culture | Men with urethritis tested by culture | 1,384 | 5.3 |
| Pareek, 1981 [149] | 1978-79 | Saudi Arabia | Hospital | CS | Conv | Urethral | Culture | Men with urethritis | 716 | 18.4 |
| Sabri, 2007 [150] | − | Iraq | Outpatient clinic | CS | Conv | Urethral | Culture | Men with urethritis | 29 | 62.1 |
| Saleem, 2009 [151] | 2004-07 | Pakistan | Community | CS | Conv | Urethral | Culture | Men with urethritis | 100 | 70.0 |
| Sellami, 2003 [152] | 1993-99 | Tunisia | Hospital | CS | Conv | Urethral | Culture | Men with urethritis | 71 | 78.9 |
| Soleimani Rahbar, 2011 [153] | − | Iran | Outpatient clinic | CS | Conv | Urethral | Culture | Men with urethritis | 106 | 60.4 |
| Waseem, 2021 [154] | 2018 | Pakistan | Outpatient clinic | CS | Conv | Urine | NAAT/PCR | Men with urethral discharge | 60 | 40.0 |
| Zabaneh, 1980 [155] | − | Lebanon | Hospital | CS | Conv | Urethral | Culture | Men with urethral discharge | 250 | 40.0 |
| Znazen, 2003 [156] | 1996-00 | Tunisia | Hospital | CS | Conv | Urethral | Culture | Men with urethritis | 133 | 54.2 |
| Zolfaghari, 2022 [121] | 2018-20 | Iran | Hospital | CS | Conv | Urethral | Culture | Symptomatic men | 32 | 3.0 |
| **Symptomatic mixed sexes** | | | | | | | | | | |
| Ali, 1996 [157] | 1993-95 | Egypt | STI clinic | CS | Conv | Urogenital | Culture | STI clinic attendees suffering from symptoms | 95 | 26.3 |
| Tsai, 2013 [158] | 2010-13 | Djibouti | Hospital | CS | Conv | Urogenital | NAAT/PCR | Symptomatic patients | 168 | 23.0 |
| **Infertility clinic attendees** | | | | | | | | | | |
| Abu Kahnjar, 2022 [159] | 2020-21 | Iraq | Outpatient clinic | CS | Conv | Semen | NAAT/PCR | Infertile men | 100 | 7.0 |
| Abusarah, 2013 [5] | 2011 | Jordan | Hospital | CC | Conv | Urine | NAAT/PCR | Urine samples from infertility clinic attendees | 81 | 0.0 |
| Ahmadi, 2022 [6] | 2014-16 | Iran | Community | CS | Conv | Endocervical | NAAT/PCR | Infertile women | 99 | 0.0 |
| Al-Douri, 2008 [160] | 2005 | Iraq | Hospital | CS | Conv | Endocervical | Culture | Infertile women | 96 | 8.3 |
| Al-Hadrawi, 2015 [161] | 2013 | Iraq | Outpatient clinic | CS | Conv | Semen | Culture | Men suffering from infertility | 328 | 0.6 |
| Al-Janabi, 2014 [162] | − | Iraq | Outpatient clinic | CS | Conv | Semen | Culture | Infertile men | 583 | 1.0 |
| Al-Muharmi, 2022 [11] | 202 | Oman | Hospital | CS | Conv | Endocervical | NAAT/PCR | Women having an infertility assessment | 165 | 0.0 |
| Azizmohammadi, 2016 [19] | 2015 | Iran | STI clinic | CC | Conv | Vaginal | Culture | Infertile women | 130 | 16.9 |
| Bakir, 1989 [163] | − | Saudi Arabia | Hospital | CS | Conv | Endocervical | Culture | Infertile women | 37 | 0.0 |
| Elkayal, 2015 [90] | − | Egypt | Hospital | CS | Conv | Endocervical | Culture | Infertility clinic attendees tested by culture | 100 | 1.0 |
| Elkayal, 2015 [90] | − | Egypt | Laboratory | CS | Conv | Endocervical | NAAT/PCR | Infertility clinic attendees tested by NAAT/PCR | 100 | 2.0 |
| Farooq Faisal, 2023 [164] | 2020-2021 | Iraq | Hospital | CS | Conv | Vaginal | Culture | Women with infertility problems | 46 | 2.2 |
| Gdoura, 2008 [165] | − | Tunisia | Outpatient clinic | CS | Conv | Semen | NAAT/PCT | Infertile men tested by PCR | 116 | 0.0 |
| Gdoura, 2008 [165] | − | Tunisia | Outpatient clinic | CS | Conv | Semen | Culture | Infertile men tested by culture | 116 | 0.0 |
| Golshani, 2006 [166] | − | Iran | Outpatient clinic | CS | Conv | Semen | Culture | Infertile men | 88 | 2.3 |
| Khoder, 2019 [38] | 2014-16 | Lebanon | Hospital | CS | Conv | Semen | NAAT/PCR | Men suspected of having infertility | 173 | 21.4 |
| Motamedifar, 2020 [47] | 2015 | Iran | Outpatient clinic | CC | Conv | Semen | NAAT/PCR | Men with infertility problems | 200 | 11.0 |
| Ramezani, 2019 [167] | − | Iran | Laboratory | CC | Conv | Semen | NAAT/PCR | Men with infertility problems | 309 | 0.3 |
| Rashidi, 2009 [51] | − | Iran | Hospital | CC | Conv | Urine | NAAT/PCR | Infertile women | 209 | 0.0 |
| Rehab, 2012 [168] | 2010 | Iraq | Outpatient clinic | CS | Conv | Semen | Culture | Infertile men | 42 | 14.3 |
| Sameni, 2022 [52] | 2018-19 | Iran | OBGYN | CS | Conv | Vaginal | NAAT/PCR | Infertile women | 65 | 6.2 |
| Sellami, 2014 [169] | − | Tunisia | Outpatient clinic | CS | Conv | Semen | NAAT/PCR | >=23 years old infertile men | 85 | 5.8 |
| Shaaban, 1994 [53] | − | Egypt | Hospital | CC | Conv | Endocervical | Culture | Women with tubal infertility | 150 | 0.7 |
| Sinan, 2008 [170] | 2007 | Iraq | Community | CS | Conv | Urogenital | NAAT/PCR | Infertile patients | 38 | 68.4 |
| Sinan, 2010 [171] | 2008 | Iraq | Community | CS | Conv | Endocervical | Culture | Infertile women | 52 | 59.6 |
| **Women with miscarriage or ectopic pregnancy** | | | | | | | | | | |
| Ahmadi, 2022 [6] | 2014-16 | Iran | Community | CS | Conv | Endocervical | NAAT/PCR | Women with spontaneous abortion | 109 | 0.0 |
| Mahdi, 1998 [101] | 1994-95 | Iraq | Hospital | CC | Conv | Vaginal | Culture | Women with miscarriages | 81 | 3.7 |
| Rajabpour, 2020 [110] | 2017-18 | Iran | Outpatient clinic | CS | Conv | Endocervical | NAAT/PCR | Women with pregnancy-related and infertility issues | 180 | 6.7 |
| Torabizadeh, 2016 [118] | 2013-14 | Iran | Outpatient clinic | CS | Conv | Vaginal | NAAT/PCR | Women with spontaneous abortion | 50 | 4.0 |
| **STI clinic attendees** | | | | | | | | | | |
| Al-Owaish, 2000 [172] | 1996-97 | Kuwait | Hospital | CS | MSRS | Urethral | Culture | Non-Kuwaiti STI clinic attendees | 1,367 | 44.1 |
| Al-Owaish, 2000 [172] | 1996-97 | Kuwait | Outpatient clinic | CS | MSRS | Urethral | Culture | Kuwaiti STI clinic attendees | 617 | 35.0 |
| Al Yazachi, 1994 [173] | 1987-88 | Iraq | Outpatient clinic | CS | Conv | Urethral | Gram stain | STI clinic attendees | 292 | 21.2 |
| Bhutto, 2011 [174] | 2000-09 | Pakistan | OBGYN | CS | Conv | Urine | Culture | STI clinic attendees | 4,288 | 14.9 |
| El Beayni, 2021 [175] | 2017-19 | Lebanon | Laboratory | CS | Conv | Urogenital | NAAT/PCR | STI clinic attendees | 597 | 2.2 |
| Heikel, 1999 [176] | 1992-96 | Morocco | STI clinic | CS | Conv | Endocervical | Culture | Female STI clinic attendees | 919 | 1.7 |
| Heikel, 1999 [176] | 1992-96 | Morocco | OBGYN | CS | Conv | Urethral | Culture | Male STI clinic attendees | 721 | 7.1 |
| WHO, 2005b [54] | − | Somalia | STI clinic | CS | Conv | Urogenital | NAAT/PCR | STI clinic attendees | 1,202 | 0.5 |
| WHO, 2005b [177] | − | Somalia | STI clinic | CS | Conv | Urogenital | NAAT/PCR | STI clinic attendees | 514 | 0.2 |
| **Individuals living with HIV and individuals in HIV-discordant couples** | | | | | | | | | | |
| Behzadi, 2018 [178] | 2015-16 | Iran | Outpatient clinic | CS | Conv | Endocervical | NAAT/PCR | Women living with HIV | 71 | 5.6 |
| Hashemi-Shahri, 2016 [179] | 2000-15 | Iran | Outpatient clinic | CS | Conv | Urine | Culture | Women living with HIV | 11 | 0.0 |
| Hashemi-Shahri, 2016 [179] | 2000-15 | Iran | Outpatient clinic | CS | Conv | Urine | Culture | Men living with HIV | 30 | 23.3 |
| **Patients with confirmed or suspected sexually transmitted infections and related infections** | | | | | | | | | | |
| Anid, 2014 [180] | 2011-12 | Iraq | Outpatient clinic | CS | Conv | Urogenital | Gram stain | Patients suspected of NG tested with Gram stain | 80 | 55.0 |
| Anid, 2014 [180] | 2011-12 | Iraq | Outpatient clinic | CS | Conv | Urogenital | Culture | Patients suspected of NG tested with culture | 80 | 55.0 |
| Anid, 2014 [180] | 2011-12 | Iraq | Outpatient clinic | CS | Conv | Urogenital | NAAT/PCR | Patients suspected of NG tested with PCR | 80 | 96.3 |
| Fageeh, 2013 [181] | 2003-11 | Saudi Arabia | Outpatient clinic | CS | Conv | Vaginal | Culture | Women with HSV | 343 | 4.7 |
| Ghafoor, 2020 [182] | 2018-19 | Iraq | Hospital | CS | Conv | Urine | Culture | Patients with gram positive and negative diplococci | 43 | 11.6 |
| Ghalib, 2013 [183] | 2007-08 | Iraq | Outpatient clinic | CS | Conv | Urogenital | Culture | Chlamydia positive women | 83 | 21.7 |
| Gharsallah, 2012 [184] | 2000-11 | Tunisia | OBGYN | CS | Conv | Endocervical | NAAT/PCR | Chlamydia positive women | 53 | 3.8 |
| Gharsallah, 2012 [184] | 2000-11 | Tunisia | OBGYN | CS | Conv | Urethral | NAAT/PCR | Chlamydia positive men | 84 | 19.0 |
| Khanani, 1994 [185] | 1989-92 | Pakistan | STI clinic | CS | Conv | Urethral | Culture | Patients with gram negative diplococci | 255 | 52.5 |
| Mortazavi, 2021 [46] | 2019-20 | Iran | Outpatient clinic | CS | Conv | Endocervical | NAAT/PCR | Women with HPV | 118 | 5.9 |
| Ranjah, 2001 [186] | − | Pakistan | Outpatient clinic | CS | Conv | Urethral | Gram stain | Urethral swabs tested using Gram staining | 80 | 93.8 |
| Ranjah, 2001 [186] | − | Pakistan | Outpatient clinic | CS | Conv | Urethral | Culture | Urethral swabs tested using culture | 80 | 61.3 |
| Taha, 1979 [187] | 1976-78 | Sudan | STI clinic | CS | Conv | Urethral | Culture | Patients with STI diagnoses | 290 | 8.6 |
| **Other populations** | | | | | | | | | |  |
| Abdelaziz, 2014 [188] | 2008 | Sudan | Outpatient clinic | CS | Conv | Urogenital | Gram stain | Symptomatic and asymptomatic pregnant women | 200 | 1.8 |
| Ahmed, 1992 [189] | 1987 | Somalia | Community | CS | Conv | Vaginal | Culture | 6-14 years old orphaned girls | 95 | 55.8 |
| Al-Khafajii, 2011 [190] | 2009-10 | Iraq | Hospital | CS | Conv | Urethral | Culture | Girls suspected of sexual abuse | 70 | 8.6 |
| Filemban, 2015 [191] | 2013-14 | Saudi Arabia | Hospital | CS | MSCRS | Urogenital | Unclear | Mix of clinical populations | 3,994 | 2.7 |
| Hassan, 2005 [31] | 2002 | Iraq | Hospital | CC | Conv | Vaginal | Culture | Women with preterm labor | 72 | 1.4 |
| Kaushik, 1999 [192] | 1994-98 | Kuwait | Hospital | CS | Conv | Urogenital | Unclear | Urogenital samples of rheumatoid arthritis patients | 36 | 2.8 |

Abbreviations: ANC = Antenatal clinic, CC = Case-control, Conv = Convenience, CS = Cross-sectional, EIA = Enzyme immunoassay, FPC = Family planning clinic, FSWs = Female sex workers, GC = Gonozyme, HIV = Human immunodeficiency virus, HPV =  Human papillomavirus, HSV = Herpes simplex virus, IUD = intrauterine device, MOH = Ministry of Health, MSCRS = Multi-stage cluster random sampling, MSM= Men who have sex with men, MSRS = Multiple stage random sampling, MSWs = Male sex workers, NAAT = Nucleic acid amplification test, NG = *Neisseria gonorrhea*, OBGYN = Obstetrics/gynecology, PCR = Polymerase chain reaction, Prev = Prevalence, PWID = People who inject drugs, RDS = Respondent driven sampling, RS = Random sampling, STI = Sexually transmitted infection, TLS = Time location sampling, UTI = Urinary tract infection, VD = Venereal disease, WHO = World Health Organization.

^a^ The "site type" refers to the location where participants were recruited for the study and may not represent the characteristics of the actual population, which are described under the "Population characteristics" column header.

# **Table S4.** Studies reporting *Neisseria gonorrhoeae* prevalence in anorectal, oropharyngeal, unspecified, or mixed anatomical sites, or serological specimens in the Middle East and North Africa.

| **Author, year** | **Year(s) of data collection** | **Country** | **Site type^a^** | **Study design** | **Sampling** | **Specimen** | **Assay** | **Population characteristics** | **Sample size** | **NG prev (%)** |
| --- | --- | --- | --- | --- | --- | --- | --- | --- | --- | --- |
| **Anorectal specimens** | | | | | | | | | | |
| **Male sex workers and men who have sex with men** | | | |  |  |  |  |  |  |  |
| Hawkes, 2009 [64] | 2007 | Pakistan | Community | CS | RDS | Anorectal | NAAT/PCR | MSWs in Rawalpindi (bantha) | 195 | 12.6 |
| Hawkes, 2009 [64] | 2007 | Pakistan | Community | CS | RDS | Anorectal | NAAT/PCR | MSWs in Rawalpindi (khotki) | 364 | 4.7 |
| Hawkes, 2009 [64] | 2007 | Pakistan | Community | CS | RDS | Anorectal | NAAT/PCR | MSWs in Rawalpindi (khusra) | 253 | 20.2 |
| Hawkes, 2009 [64] | 2007 | Pakistan | Community | CS | RDS | Anorectal | NAAT/PCR | MSWs in Abbottabad (bantha) | 83 | 11.1 |
| Hawkes, 2009 [64] | 2007 | Pakistan | Community | CS | RDS | Anorectal | NAAT/PCR | MSWs in Abbottabad | 20 | 5.0 |
| Rehan, 2009 [193] | 2004 | Pakistan | Community | CS | MSCRS | Anorectal | NAAT/PCR | Hijra in Karachi | 197 | 29.4 |
| Rehan, 2009 [193] | 2004 | Pakistan | Prison | CS | RDS | Anorectal | NAAT/PCR | MSWs in Lahore | 400 | 0.0 |
| Rehan, 2009 [193] | 2004 | Pakistan | STI clinic | CS | Snowball | Anorectal | NAAT/PCR | MSWs in Karachi | 395 | 17.5 |
| Hancali, 2019 [194] | 2017 | Morocco | Community | CS | RDS | Anorectal | NAAT/PCR | MSM | 238 | 8.4 |
| **Symptomatic women** |  |  |  |  |  |  |  |  |  |  |
| Sallam, 1982 [114] | 1979 | Egypt | Outpatient clinic | CS | RS | Anorectal | Culture | Rectal swabs of women with vaginal discharge | 200 | 3.0 |
| **Oropharyngeal specimens** | | | | | | | | | | |
| **General populations** |  |  |  |  |  |  |  |  |  |  |
| Soleimani Rahbar, 2008 [153]^b^ | − | Iran | Outpatient clinic | CS | Conv | Oropharyngeal | Culture | Healthy persons | 230 | 99.1 |
| **Mixed/Unclear specimens** | | | | | | | | | | |
| **General populations** |  |  |  |  |  |  |  |  |  |  |
| Gouya, 2007 [195] | − | Iran | OBGYN | CS | Conv | Unclear | Culture | FPC attendees | 150 | 2.0 |
| Hancali, 2015 [196] | 2011 | Morocco | Outpatient clinic | CS | Conv | Unclear | NAAT/PCR | FPC clinic attendees - 2011 | 256 | 0.9 |
| Hancali, 2015 [196] | 1999 | Morocco | Antenatal clinic | CS | Conv | Unclear | NAAT/PCR | FPC clinic attendees - 1999 | 760 | 0.7 |
| Hasanabad, 2013 [197] | − | Iran | Antenatal clinic | CS | Conv | Unclear | NAAT/PCR | Pregnant adolescent women | 399 | 1.3 |
| Mahmood, 2011 [198] | − | Pakistan | Community | CS | Conv | Unclear | Unclear | Married women | 385 | 2.6 |
| MOH - Algeria, 2002 [199] | 2002 | Algeria | Unclear | CS | Conv | Unclear | Unclear | Ever-married women | 14,481 | 0.4 |
| MOH - Iran, 2008 [43] | 1996 | Iran | Unclear | CS | Conv | Unclear | Unclear | Gynecology clinic attendees | 165 | 0.6 |
| **Intermediate risk populations** | |  |  |  |  |  |  |  |  |  |
| MOH - Iran, 2008 [43] | 2007 | Iran | ANC | CS | Conv | Unclear | NAAT/PCR | Male prisoners | 400 | 0.5 |
| **Female sex workers** |  |  |  |  |  |  |  |  |  |  |
| Burans, 1990 [200] | 1987 | Somalia | ANC | CS | Conv | Unclear | Culture | FSWs | 89 | 11.2 |
| **Male sex workers and men who have sex with men** | | | |  |  |  |  |  |  |  |
| Osama, 2017 [201] | − | Pakistan | Outpatient clinic | CS | Conv | Unclear | Unclear | MSM | 2,531 | 36.1 |
| **Symptomatic women** |  |  |  |  |  |  |  |  |  |  |
| Al Jaufy, 2007 [202] | 2004-05 | Yemen | OBGYN | CS | Conv | Unclear | Unclear | Symptomatic women | 100 | 4.0 |
| Alwazer, 2004 [203] | − | Yemen | OBGYN | CS | Conv | Unclear | Unclear | Symptomatic women | 200 | 5.0 |
| Bokaeian, 2010 [204] | 2005-08 | Iran | ANC | CS | Conv | Unclear | Culture | Women suffering from urethritis and purulent cervicitis | 400 | 19.2 |
| Ramia, 2012 [205] | 2009 | Lebanon | Community | RCT | Conv | Unclear | NAAT/PCR | Women with vaginal discharge | 441 | 1.0 |
| Rushwan, 1980 [206] | 1976 | Sudan | ANC | CS | Conv | Unclear | Unclear | Women with vaginal discharge | 147 | 1.4 |
| **Symptomatic men** |  |  |  |  |  |  |  |  |  |  |
| Hancali, 2015 [196] | 2001 | Morocco | Hospital | CS | Conv | Unclear | NAAT/PCR | Men with urethral discharge - 2001 | 422 | 41.6 |
| Zargooshi, 2002 [207] | 1997-00 | Iran | Outpatient clinic | Cohort | Conv | Unclear | Gram stain | Men with urethritis | 162 | 67.3 |
| **Infertility clinic attendees** | |  |  |  |  |  |  |  |  |  |
| Jaballah, 1987 [208] | − | Tunisia | Outpatient clinic | CS | Conv | Unclear | Unclear | Infertile men | 373 | 14.2 |
| **STI clinic attendees** |  |  |  |  |  |  |  |  |  |  |
| Ashraf, 2020 [209] | 2016-17 | Pakistan | Outpatient clinic | CS | Conv | Unclear | Unclear | STI clinic attendees | 285 | 2.1 |
| Burans, 1990 [200] | 1987 | Somalia | ANC | CS | Conv | Unclear | Culture | Male STI clinic attendees | 45 | 6.7 |
| Ghanaat, 2003 [210] | 1998-00 | Iran | Prison | CS | Conv | Unclear | Culture | STI clinic attendees | 1,500 | 6.0 |
| Maan, 2011 [211] | 2006-09 | Pakistan | Outpatient clinic | CS | Conv | Unclear | Culture | STI clinic attendees | 1,532 | 13.0 |
| Maatouk, 2020 [212] | 2014-18 | Lebanon | STI clinic | CS | Conv | Unclear | Unclear | MSM visiting a STI clinic | 1,364 | 22.9 |
| Razvi, 2014 [213] | 2010-14 | Pakistan | Laboratory | CS | Conv | Unclear | Unclear | STI clinic attendees | 512 | 45.1 |
| Rehan 2003 [61] | 1999 | Pakistan | Community | CS | Conv | Unclear | Unclear | STI clinic attendees | 465 | 27.5 |
| **Individuals living with HIV and individuals in HIV-discordant couples** | | | |  |  |  |  |  |  |  |
| Ghassabi, 2018 [214] | 2004-13 | Iran | HIV clinic | CS | Conv | Unclear | Culture | Patients living with HIV | 806 | 2.6 |
| **Other populations** |  |  |  |  |  |  |  |  |  |  |
| Al-mahroos, 2011 [215] | 2000-09 | Bahrain | Hospital | CS | Conv | Unclear | Unclear | Children victims of sexual abuse | 440 | 2.0 |
| Singh, 2018 [216] | 2011-17 | Pakistan | Hospital | CS | Conv | Unclear | Culture | Children with suspected STI | 57 | 13.4 |
| **Serological specimens** | | | | | | | | | | |
| **General populations** |  |  |  |  |  |  |  |  |  |  |
| Almroth, 2005 [217] | 2003-04 | Sudan | Outpatient clinic | CC | Conv | Sera / Blood | IgG | ANC clinic attendees | 137 | 2.0 |
| Dhamraa, 2008 [218] | − | Iraq | Outpatient clinic | CC | Conv | Sera / Blood | Unclear | Women with full term delivery | 30 | 0.0 |
| Diab, 1993 [219] | − | Egypt | Community | CC | Conv | Sera / Blood | IgG | Women with full-term deliveries | 30 | 0.0 |
| **Infertility clinic attendees** | |  |  |  |  |  |  |  |  |  |
| Almroth, 2005 [217] | 2003-04 | Sudan | Hospital | CC | Conv | Sera / Blood | IgG | Women with primary infertility | 79 | 3.0 |
| **Women with miscarriage or ectopic pregnancy** | | | |  |  |  |  |  |  |  |
| Dhamraa, 2008 [218] | − | Iraq | Outpatient clinic | CC | Conv | Sera / Blood | Unclear | Women with miscarriages | 60 | 0.0 |
| Diab, 1993 [219] | − | Egypt | Community | CC | Conv | Sera / Blood | IgG | Women with ectopic Pregnancy | 30 | 13.3 |
| **Other populations** |  |  |  |  |  |  |  |  |  |  |
| Elmusharaf, 2006 [220] | 2003-04 | Sudan | OBGYN | CC | Conv | Sera / Blood | Indirect hemagglutination | Pregnant and infertile women | 222 | 2.3 |
| Kaushik, 1999 [192] | 1994-98 | Kuwait | Hospital | CS | Conv | Sera / Blood | Unclear | Blood of rheumatoid arthritis patients | 36 | 11.1 |

Abbreviations: ANC = Antenatal clinic, CC = Case-control, Conv = Convenience, CS = Cross-sectional, FPC = Family planning clinic, FSWs = Female sex workers, HIV = Human immunodeficiency virus, IgG = Immunoglobulin G, MOH = Ministry of Health, MSCRS = Multi-stage cluster random sampling, MSM= Men who have sex with men, MSWs = Male sex workers, NAAT = Nucleic acid amplification test, NG = *Neisseria gonorrhea*, OBGYN = Obstetrics/gynecology, PCR = Polymerase chain reaction, Prev = Prevalence, RCT = Randomized controlled trial, RDS = Respondent driven sampling, RS = Random sampling, STI = Sexually transmitted infection.

^a^ The "site type" refers to the location where participants were recruited for the study and may not represent the characteristics of the actual population, which are described under the "Population characteristics" column header.

^b^ The publication reporting this measure has insufficient clarity in its methods, making it difficult to determine the accuracy of the reported prevalence.

# **Table S5.** Results of meta-analyses on studies reporting urogenital *Neisseria gonorrhoeae* prevalence in general populations by MENA country, and study precision.

| **General populations** | **Stratified prevalence measures** | **Sample** | **NG prevalence (%)** | | **Pooled mean NG prevalence** | **Heterogeneity measures** | | |
| --- | --- | --- | --- | --- | --- | --- | --- | --- |
|  |  | **size** |  |  |  |  |  |  |
|  | **Total** | **Total** | **Range** | **Median** | **Mean (%)** | **Q^a^** | **I²^b^ (%)** | **Prediction interval**^c^ **(%)** |
|  | **n** | **N** |  |  | **(95% CI)** | **(p-value)** | **(95% CI)** |  |
| **By MENA country** | |  |  |  |  |  |  |  |
| Egypt | 4 | 802 | 2.0-30.0 | 2.4 | 6.2 (0.0-19.3) | 29.5 (p<0.001) | 89.9 (76.9-95.5) | 0.0-87.5 |
| Iran | 26 | 13,289 | 0.0-20.0 | 0.7 | 0.8 (0.3-1.4) | 94.8 (p<0.001) | 73.6 (61.2-82.1) | 0.0-4.5 |
| Iraq | 16 | 1,765 | 0.0-40.0 | 4.8 | 7.0 (2.8-12.7) | 120.3 (p<0.001) | 87.5 (81.3-91.7) | 0.0-38.2 |
| Jordan | 4 | 604 | 0.0-2.2 | 0.3 | 0.5 (0.0-1.7) | 4.8 (p=0.191) | 36.8 (0.0-78.2) | 0.0-7.2 |
| Kuwait | 2 | 8,539 | 1.4-1.6 | 1.5 | 1.4 (1.2-1.7)^d^ | - | - | - |
| Lebanon | 7 | 1,445 | 0.0-12.2 | 1.0 | 1.2 (0.0-4.2) | 55.1 (p<0.001) | 89.1 (80.0-94.1) | 0.0-17.1 |
| Morocco | 5 | 3,240 | 0.4-14.2 | 1.4 | 4.3 (0.4-11.9) | 277.7 (p<0.001) | 98.6 (97.9-99.0) | 0.0-48.9 |
| Oman | 3 | 372 | 0.0-2.2 | 0.0 | 0.0 (0.0-0.9) | 3.2 (p=0.200) | 38.0 (0.0-80.5) | 0.0-29.2 |
| Pakistan | 4 | 3,544 | 0.0-13.3 | 0.1 | 1.1 (0.0-7.1) | 29.0 (p<0.00) | 89.7 (76.4-95.5) | 0.0-58.9 |
| Saudi Arabia | 2 | 239 | 0.0-2.1 | 1.1 | 1.3 (0.3-3.6)^d^ | - | - | - |
| Somalia | 4 | 5,622 | 0.0-0.8 | 0.2 | 0.4 (0.0-0.8) | 4.7 (p=0.200) | 35.5 (0.0-77.5) | 0.0-2.7 |
| Sudan | 3 | 827 | 0.9-2.0 | 1.2 | 1.2 (0.5-2.1) | 1.1 (p=0.587) | 0.0 (0.0-89.6) | 0.0-10.8 |
| Yemen | 1 | 137 | - | - | 1.5 (0.2-5.2) | - | - | - |
| **By study precision** |  |  |  |  |  |  |  |  |
| Sample size <200 | 46 | 4,226 | 0.0-40.0 | 1.8 | 3.1 (1.5-5.0) | 289.7 (p<0.001) | 84.5 (80.1-87.9) | 0.0-22.1 |
| Sample size ≥200 | 35 | 36,199 | 0.0-14.2 | 0.7 | 1.1 (0.5-1.9) | 831.6 (p<0.001) | 95.9 (95.0-96.6) | 0.0-8.0 |
| **Overall** | **81** | **40,425** | **0.0-40.0** | **1.0** | **1.9 (1.1-2.8)** | **1,161.5 (p<0.001)** | **93.1 (92.0-94.1)** | **0.0-14.5** |

Abbreviation: CI = Confidence interval, MENA = Middle East and North Africa, NAAT = Nucleic acid amplification test, NG = *Neisseria gonorrhoeae*, PCR = Polymerase chain reaction.

A minimum of three studies were required to conduct a meta-analysis.

^a^ Q: The Cochran's Q statistic is a measure assessing the existence of heterogeneity in pooled outcome measures, here NG prevalence.

^b^ I^2^: A measure that assesses the magnitude of between-study variation that is due to actual differences in NG prevalence across studies rather than chance.

^c^ Prediction interval: A measure that estimates the distribution (95% interval) of true NG prevalence around the estimated mean.

^d^ Two prevalence measures are not sufficient to conduct a random-effects meta-analysis. The pooled measure was calculated as the arithmetic mean of the two measures and their 95% confidence intervals.

# **Table S6.** Summary of precision assessment and risk of bias assessment for studies reporting *Neisseria gonorrhoeae* prevalence in the Middle East and North Africa.

| **Quality assessment** | ***Neisseria gonorrhea* prevalence measures** | |
| --- | --- | --- |
|  | **Number of studies** | **%** |
| **Precision of prevalence measures^a^** | | |
| Low precision | 189 | 55.4 |
| High precision | 152 | 44.6 |
| **Risk of bias quality domain^b^** | | |
| **Sampling method** | | |
| Low risk of bias | 50 | 14.7 |
| High risk of bias | 291 | 85.3 |
| **Response rate** | | |
| Low risk of bias | 168 | 49.3 |
| High risk of bias | 15 | 4.4 |
| Unclear risk of bias | 158 | 46.3 |
| **Summary of the risk of bias assessment** | | |
| **Low risk of bias** |  |  |
| In at least one quality domain | 195 | 57.2 |
| In both quality domains | 23 | 6.7 |
| **High risk of bias** |  |  |
| In at least one quality domain | 306 | 89.7 |
| In both quality domains | 0 | 0.0 |
| **Prevalence studies where risk of bias assessment was possible** | **341** | **100** |

^a^ Precision was assessed based on the overall sample size (not each stratum subsample size) of the study as reported in the record/publication.

^b^ Risk of bias was assessed based on the overall sample size (not each stratum subsample size) of the study as reported in the record/publication.

# **Fig S1.** Forest plots presenting outcomes of the pooled mean *Neisseria gonorrhoeae* prevalence in urogenital specimens among different populations in the Middle East and North Africa.

1.
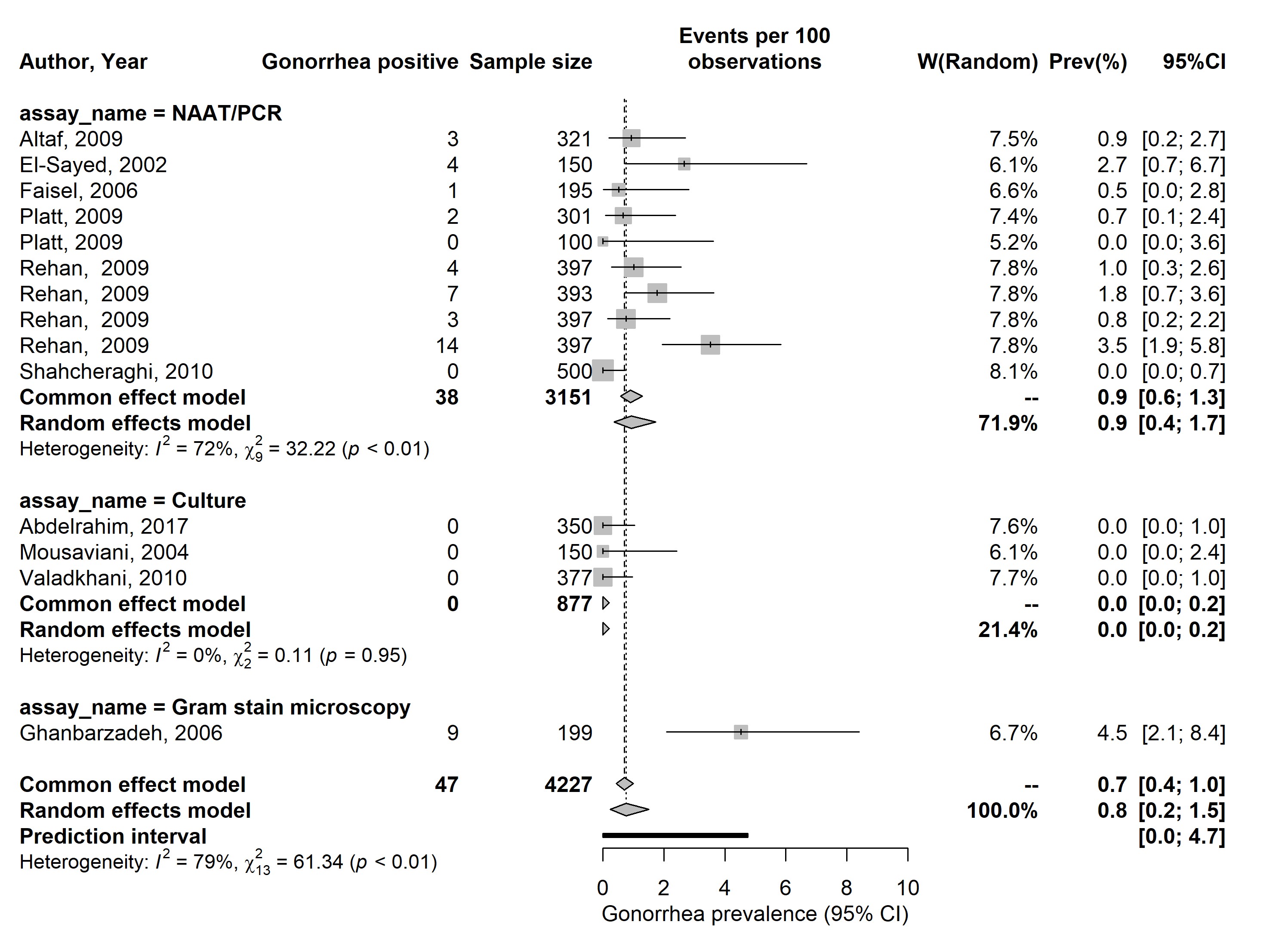
Intermediate risk populations

Abbreviations: NAAT = Nucleic acid amplification test, PCR = Polymerase chain reaction.

1.
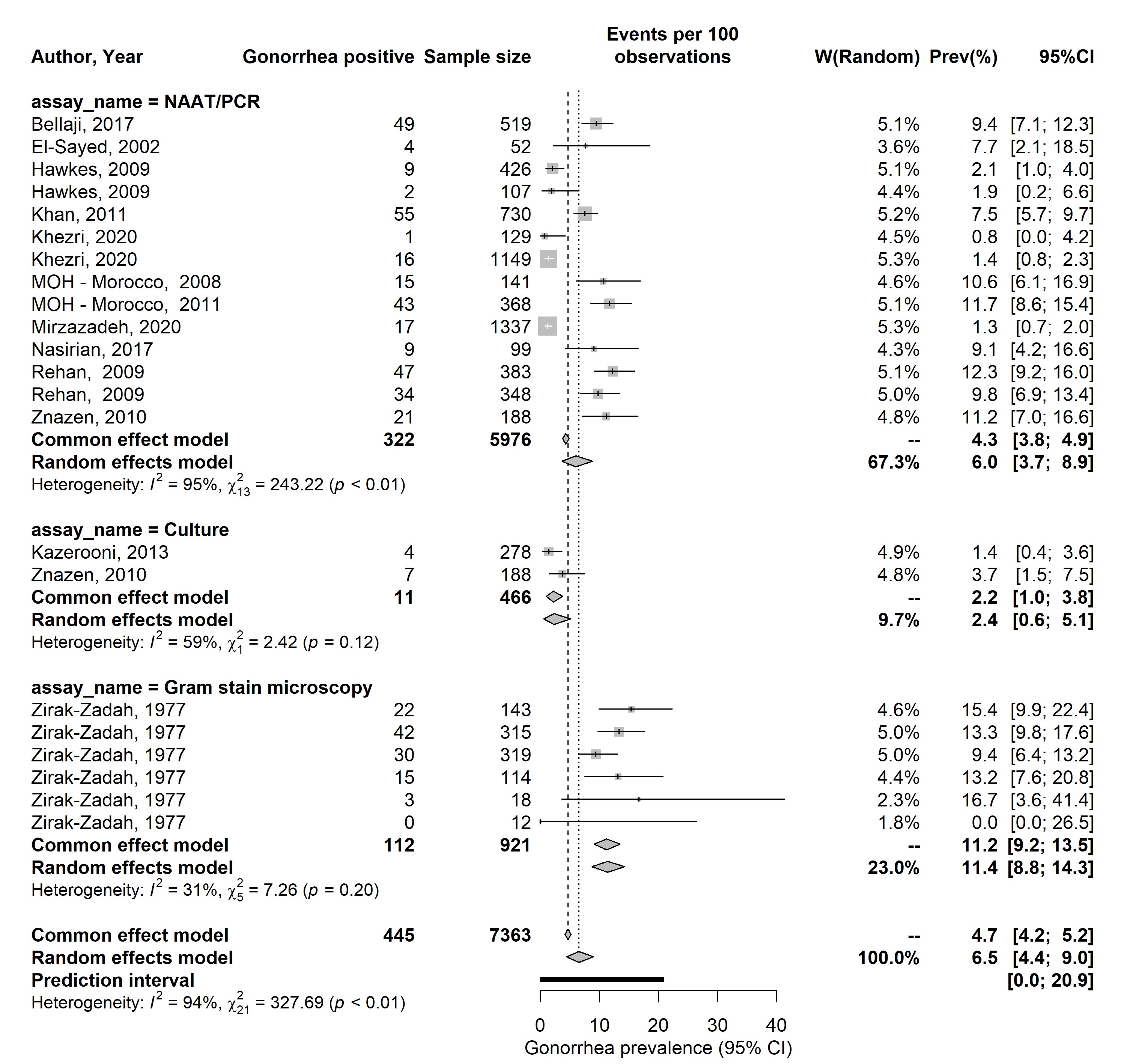
Female sex workers

Abbreviations: NAAT = Nucleic acid amplification test, PCR = Polymerase chain reaction.

1. ^
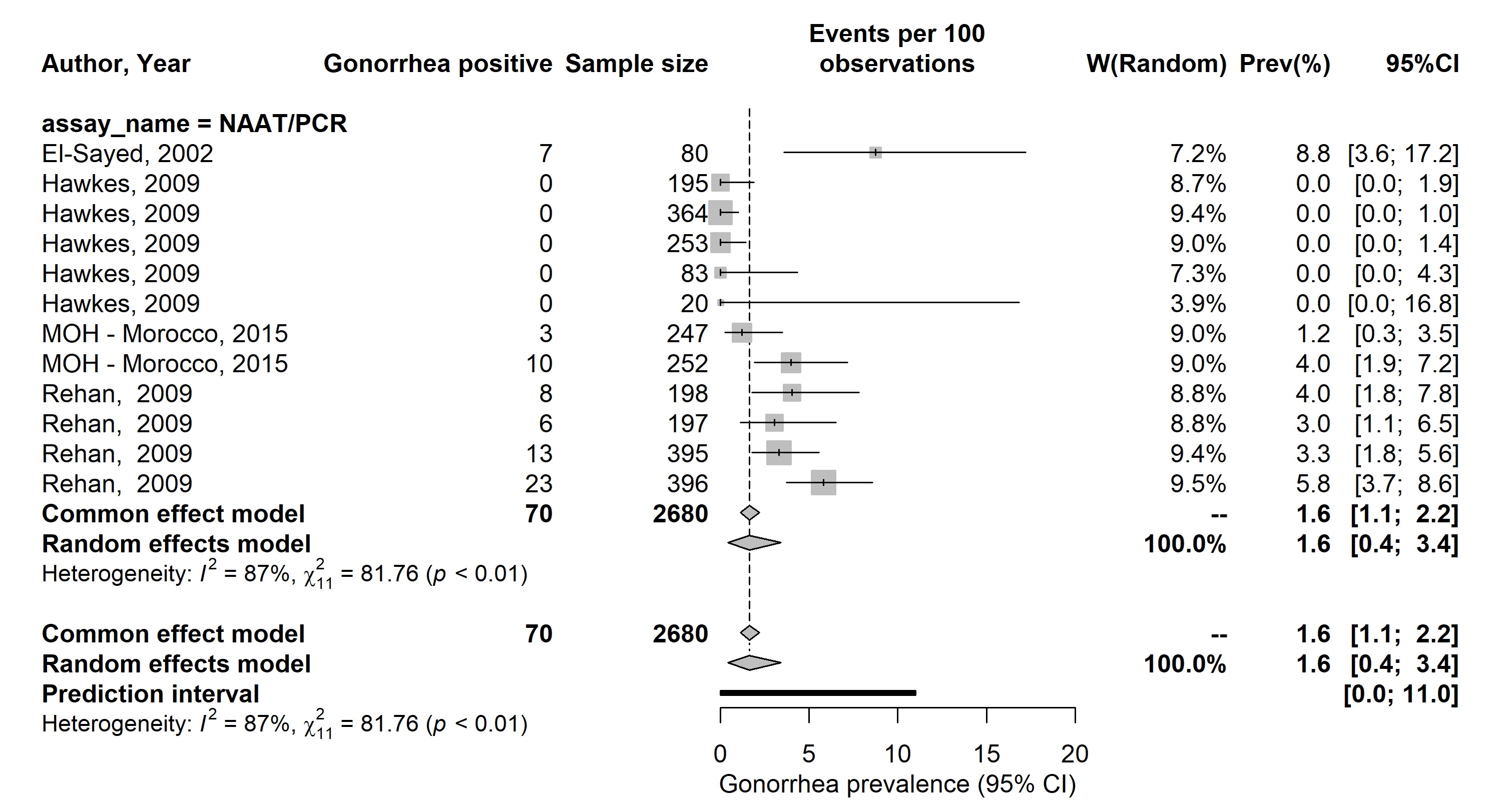
^Male sex workers and men who have sex with men^a^

Abbreviations: NAAT = Nucleic acid amplification test, PCR = Polymerase chain reaction.

^a^The majority of studies were on male sex workers, primarily from Pakistan, while a smaller proportion of studies were on men who have sex with men.

1.
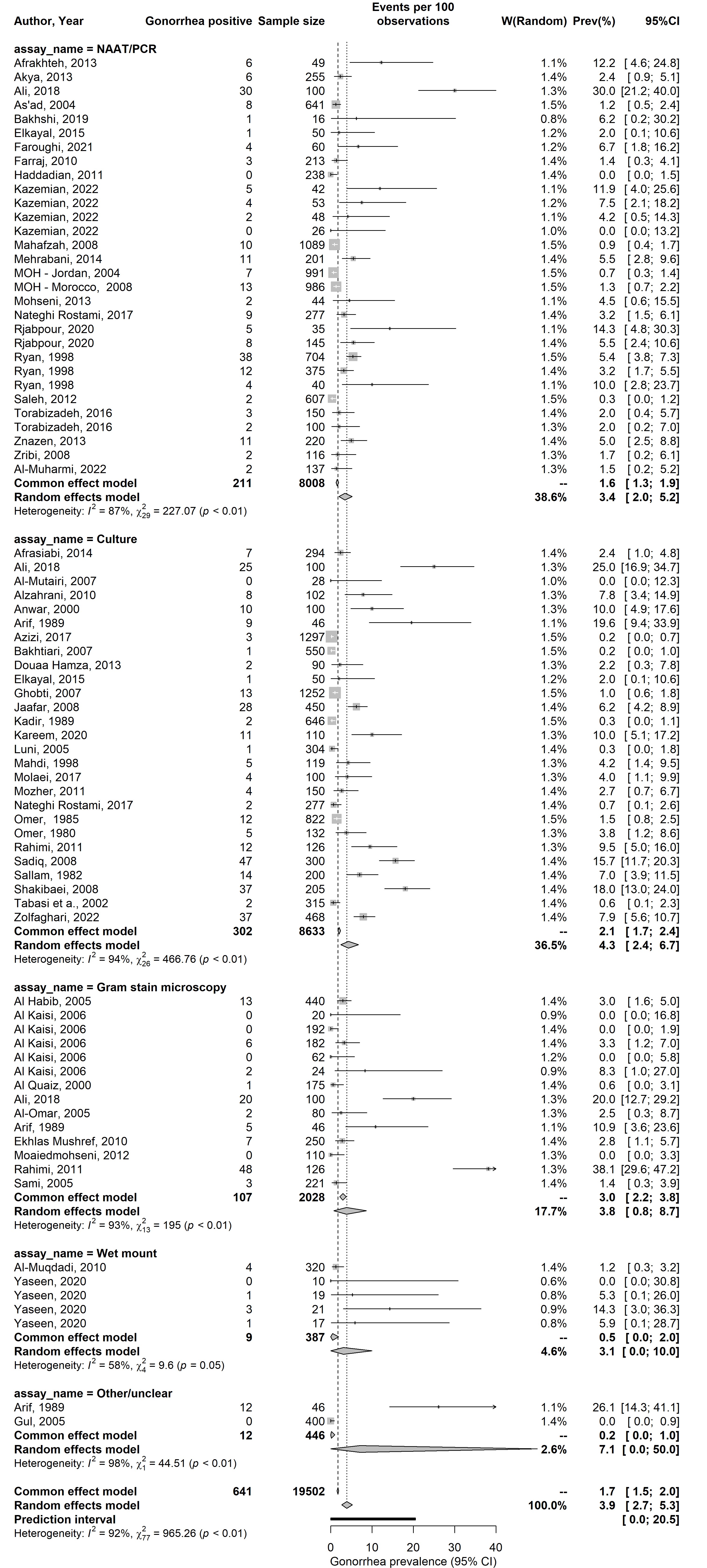
Symptomatic women

Abbreviations: NAAT = Nucleic acid amplification test, PCR = Polymerase chain reaction.

1.
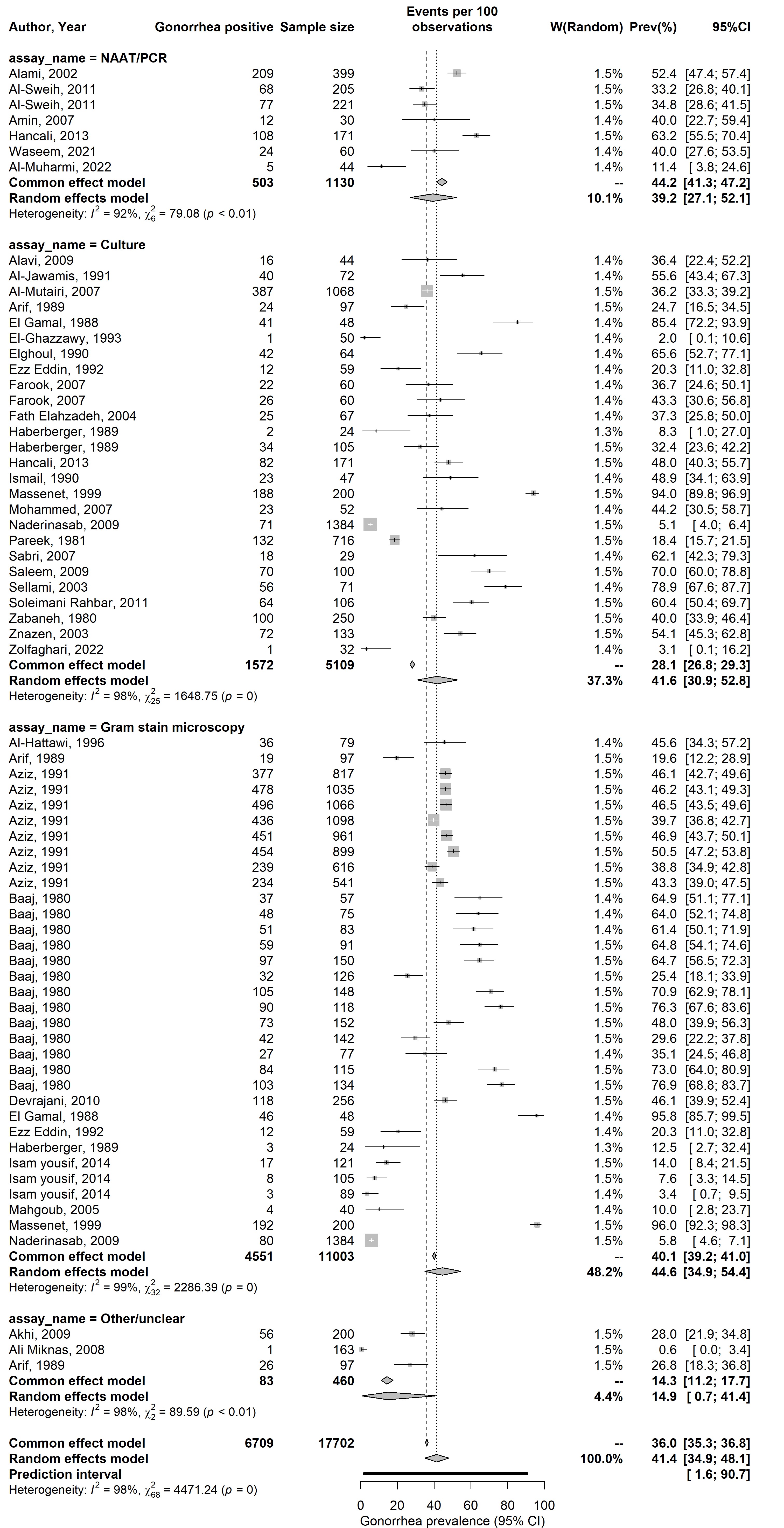
Symptomatic men

Abbreviations: NAAT = Nucleic acid amplification test, PCR = Polymerase chain reaction.

1.
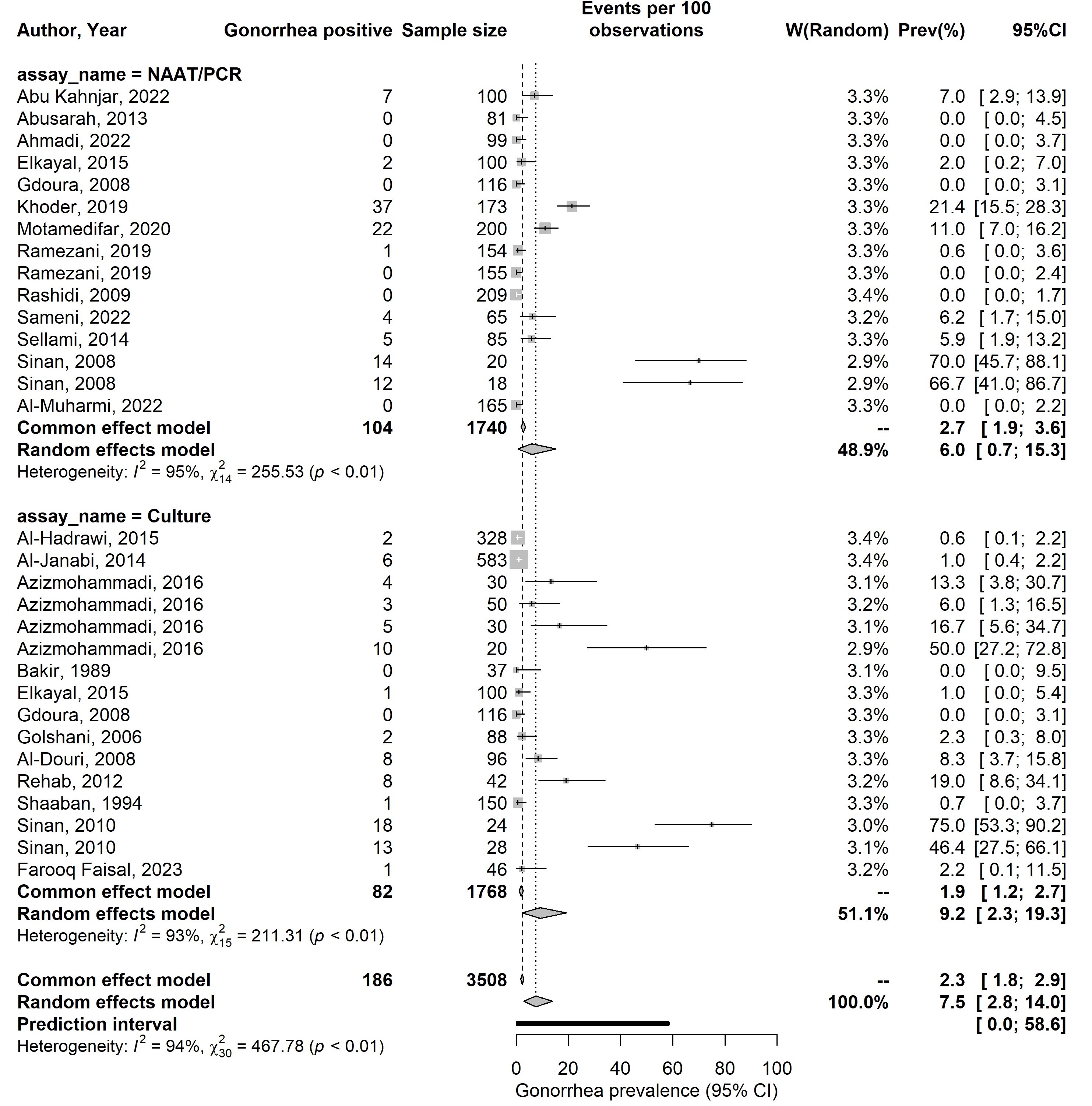
Infertility clinic attendees

Abbreviations: NAAT = Nucleic acid amplification test, PCR = Polymerase chain reaction.

1.
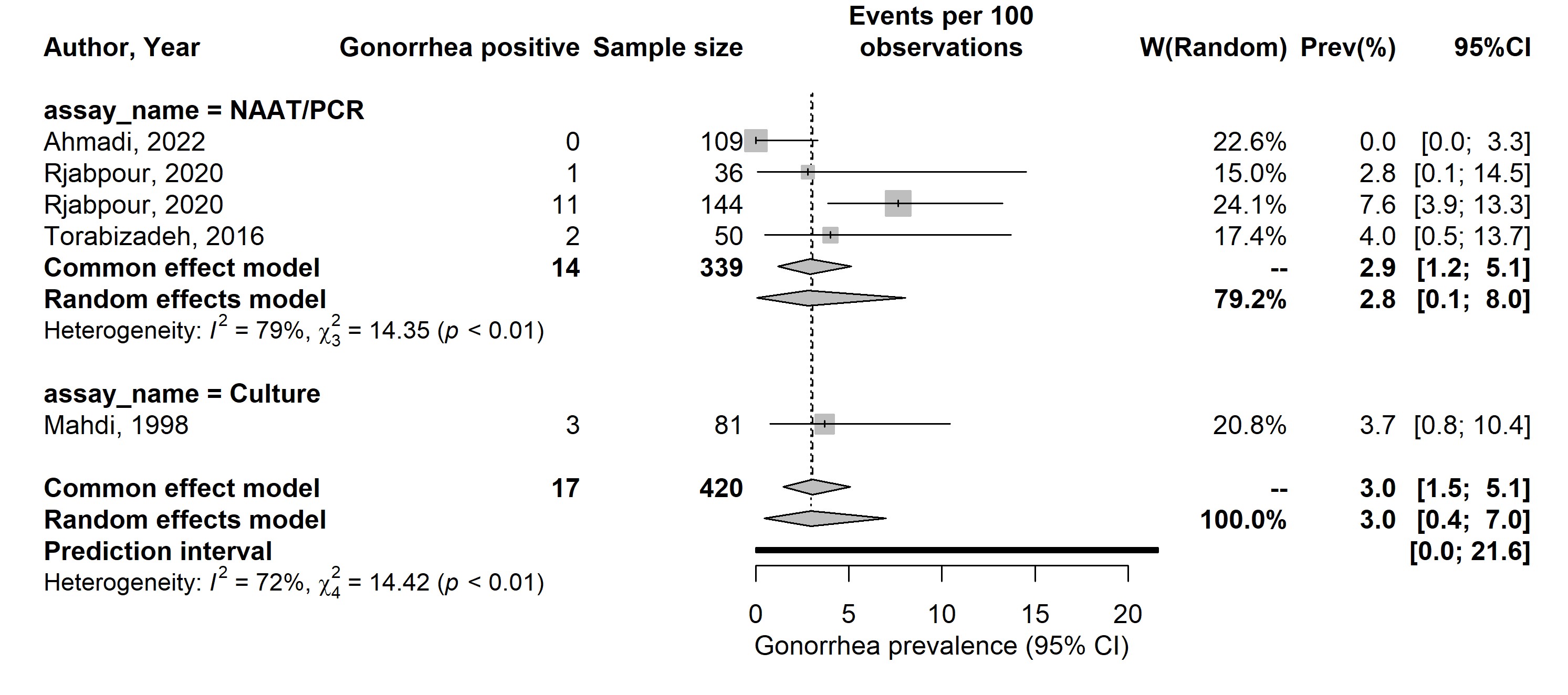
Women with miscarriage or ectopic pregnancy

Abbreviations: NAAT = Nucleic acid amplification test, PCR = Polymerase chain reaction.

1.
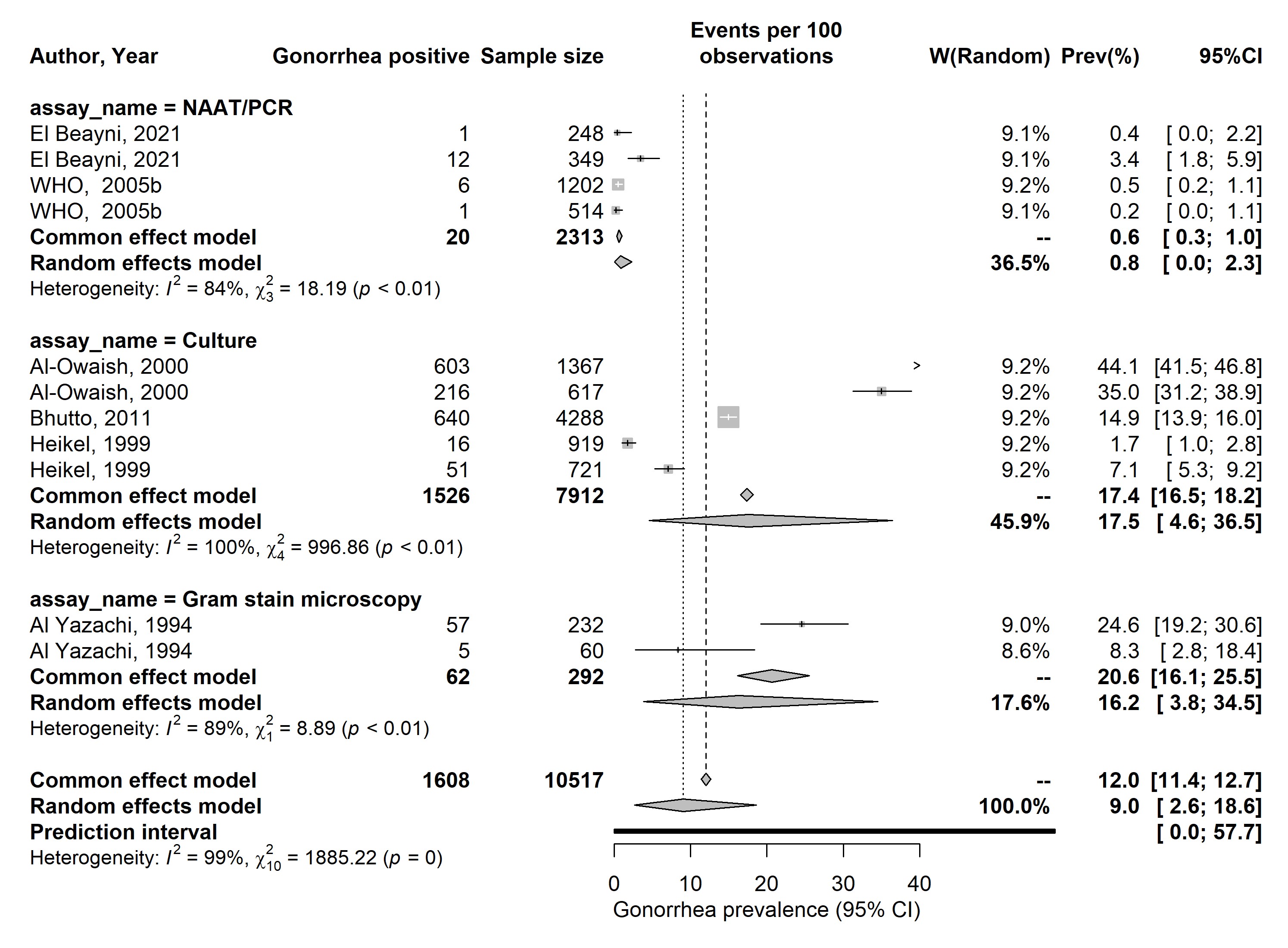
STI clinic attendees

Abbreviations: NAAT = Nucleic acid amplification test, PCR = Polymerase chain reaction, STI = Sexually transmitted infections.

1.
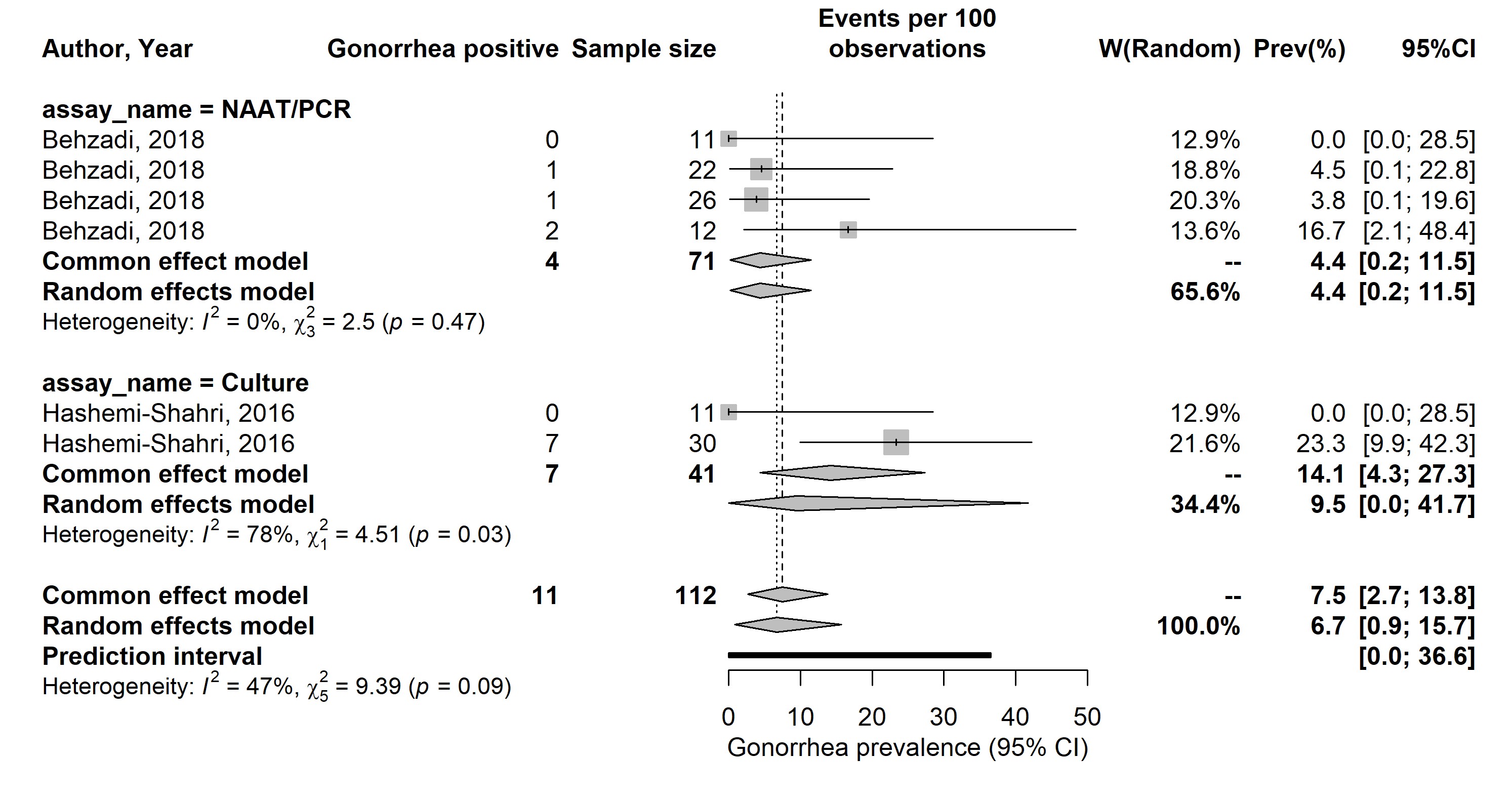
 Individuals living with HIV and individuals in HIV-discordant couples

Abbreviations: HIV = Human immunodeficiency virus, NAAT = Nucleic acid amplification test, PCR = Polymerase chain reaction.

1.
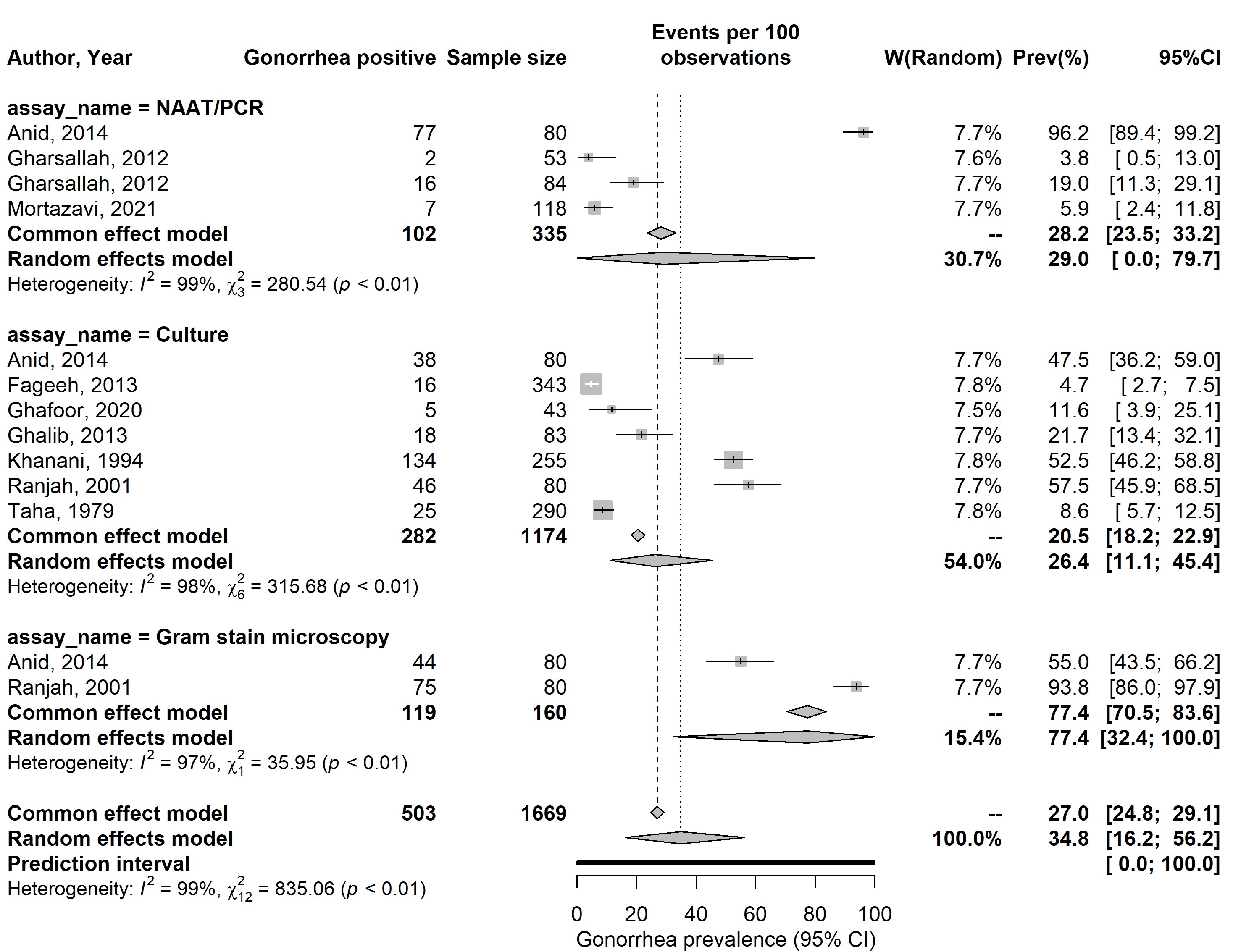
Patients with confirmed or suspected sexually transmitted infections and related infections

Abbreviations: NAAT = Nucleic acid amplification test, PCR = Polymerase chain reaction.

1.
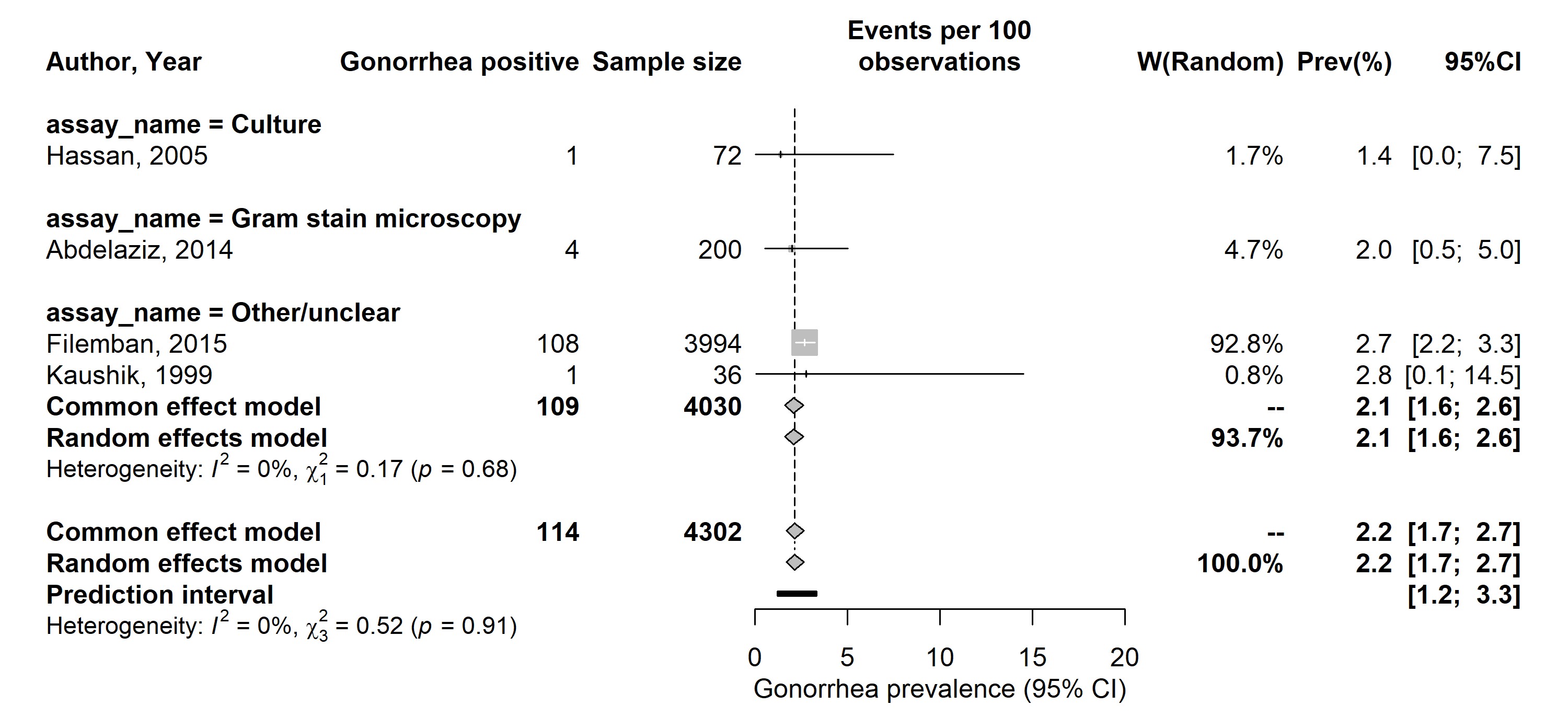
Other populations

# **Fig S2.** Forest plots presenting outcomes of the pooled mean *Neisseria gonorrhoeae* prevalence in anorectal, oropharyngeal, unspecified or mixed anatomical sites, or serological specimens among different populations in the Middle East and North Africa.

1.
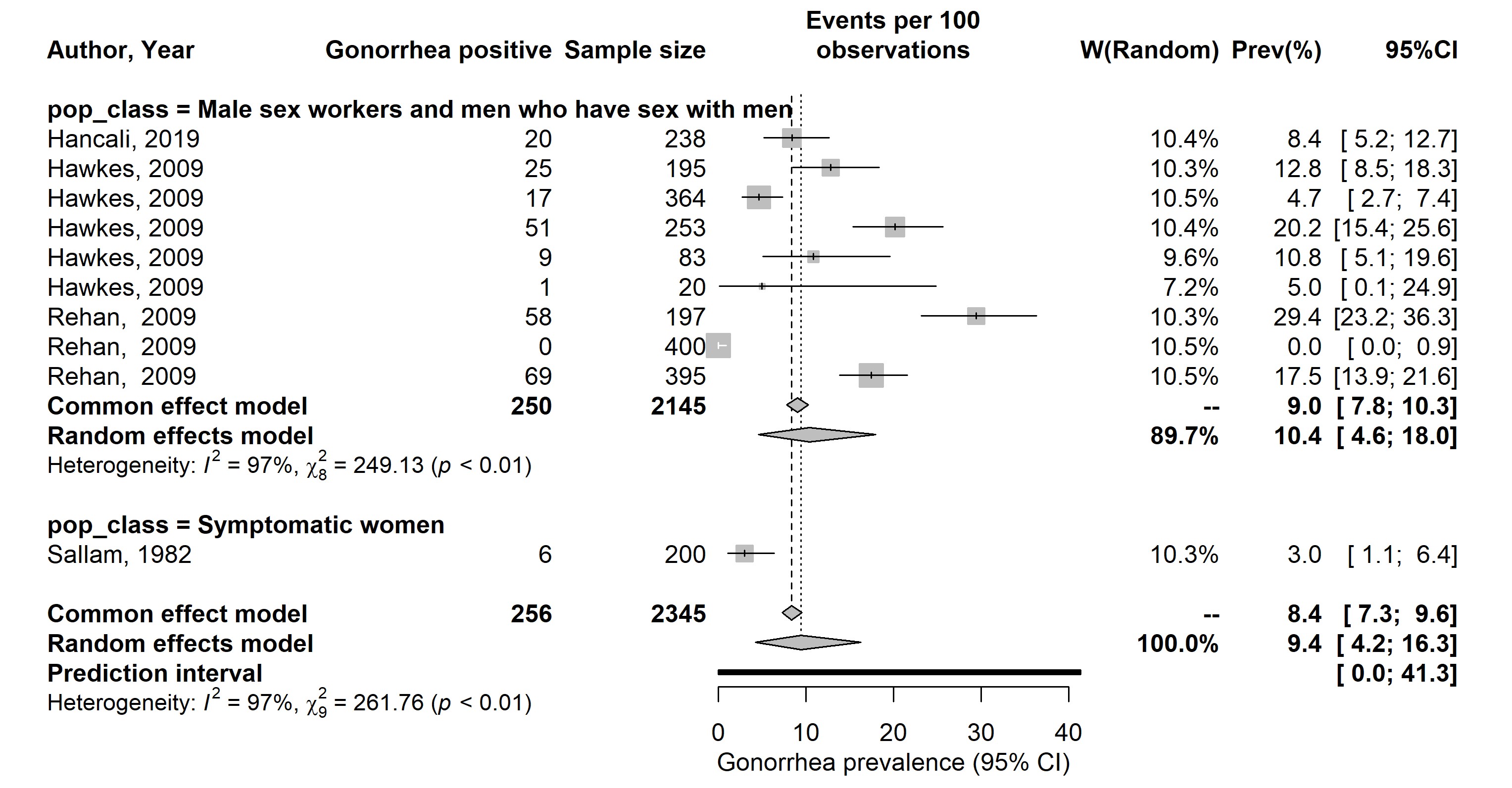
Anorectal specimen^a^

^a^ The majority of studies were on male sex workers, primarily from Pakistan, while a smaller proportion of studies were on men who have sex with men.

1. Unspecified or mixed anatomical sites


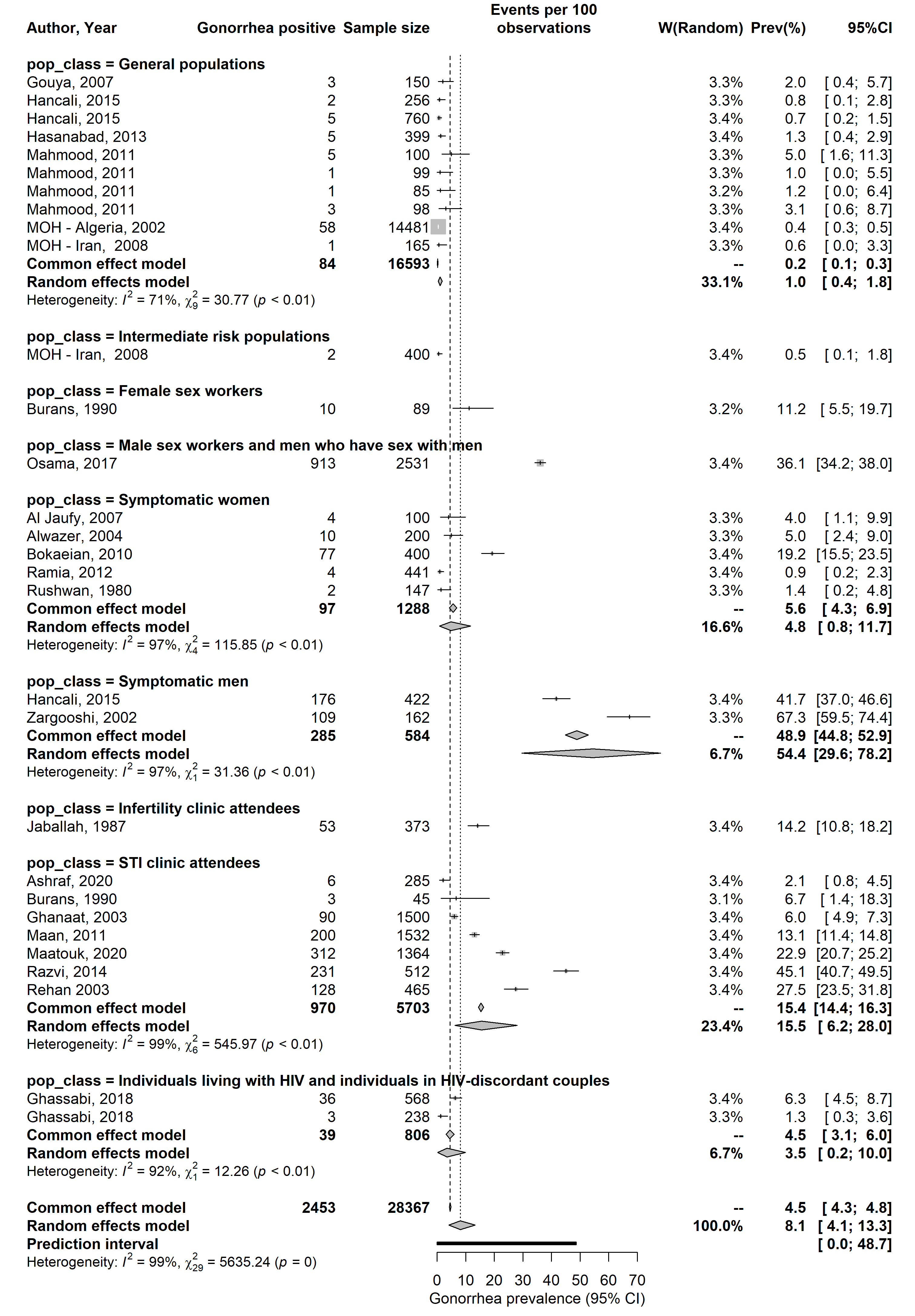


Abbreviations: HIV = Human immunodeficiency virus, STI = Sexually transmitted infections.

1.
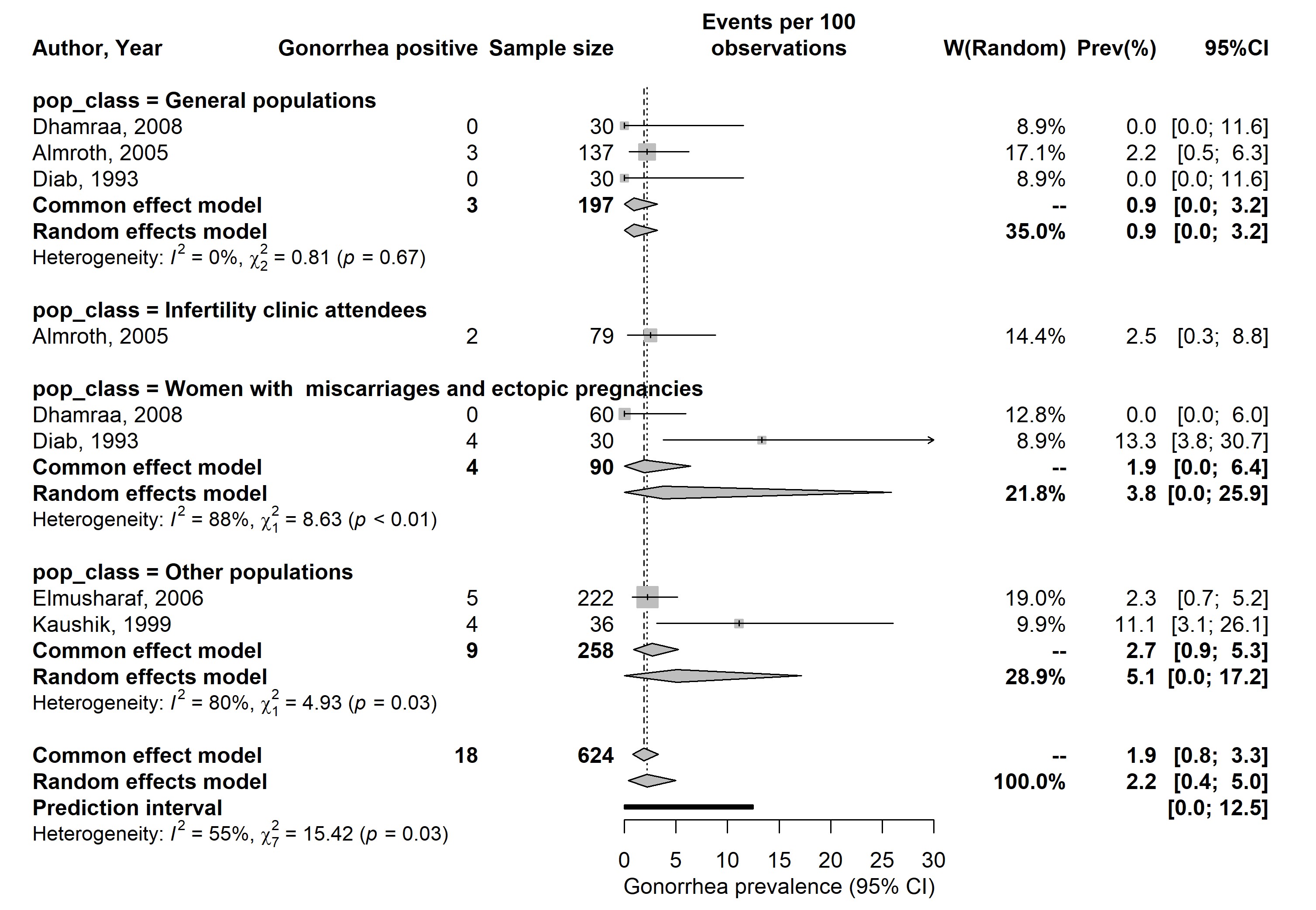
Serological specimens

# **Table S7.** Sensitivity analysis. Univariable and multivariable meta-regression analyses for *Neisseria gonorrhoeae* prevalence in urogenital specimens in the Middle East and North Africa using the year of publication as the time variable instead of the year of data collection.

| **Urogenital specimens** | | | **Stratified prevalence measures** | **Sample size** | **Univariable analysis** | | | | **Multivariable analyses** | | | |
| --- | --- | --- | --- | --- | --- | --- | --- | --- | --- | --- | --- | --- |
|  |  |  | **Total n** | **Total N** | **RR (95% CI)** | **p-value** | **LT test p-value** | **Adjusted R^2^** | **Model 1** | | **Model 2** | |
|  |  |  |  |  |  |  |  |  | **ARR (95% CI)** | **p-value** | **ARR (95% CI)** | **p-value** |
| **Population characteristics** | **Population type^a^** | General populations | 81 | 40,425 | 1.00 | - | <0.001 | 21.3 | 1.00 | - | 1.00 | - |
|  |  | Intermediate-risk populations | 14 | 4,227 | 0.61 (0.27-1.41) | 0.250 |  |  | 0.51 (0.21-1.24) | 0.136 | 0.46 (0.19-1.11) | 0.082 |
|  |  | FSWs | 22 | 7,363 | 2.61 (1.48-4.61) | 0.001 |  |  | 3.56 (2.10-6.05) | <0.001 | 3.25 (1.90-5.58) | <0.001 |
|  |  | MSWs and MSM^b^ | 12 | 2,680 | 1.53 (0.63-3.71) | 0.346 |  |  | 0.92 (0.36-2.35) | 0.853 | 0.83 (0.32-2.12) | 0.690 |
|  |  | Symptomatic women | 78 | 19,502 | 1.65 (1.09-2.49) | 0.017 |  |  | 1.86 (1.26-2.74) | 0.002 | 1.80 (1.22-2.65) | 0.003 |
|  |  | Symptomatic men | 69 | 17,702 | 14.50 (9.80-21.70) | <0.001 |  |  | 7.02 (3.75-13.10) | <0.001 | 6.52 (3.48-12.20) | <0.001 |
|  |  | Symptomatic patients (mixed sexes) | 2 | 263 | 10.30 (2.23-47.50) | 0.003 |  |  | 7.31 (1.77-30.10) | 0.006 | 7.23 (1.77-29.40) | 0.006 |
|  |  | Infertility clinic attendees | 31 | 3,508 | 3.70 (2.09-6.53) | <0.001 |  |  | 3.05 (1.75-5.31) | <0.001 | 2.85 (1.65-4.94) | <0.001 |
|  |  | Women with miscarriage or ectopic pregnancy | 5 | 420 | 1.86 (0.53-6.52) | 0.331 |  |  | 1.69 (0.55-5.25) | 0.360 | 1.68 (0.54-5.18) | 0.368 |
|  |  | STI clinic attendees | 11 | 10,517 | 2.37 (1.13-4.97) | 0.022 |  |  | 2.61 (1.26-5.40) | 0.010 | 2.73 (1.32-5.63) | 0.007 |
|  |  | Individuals living with HIV and individuals in HIV discordant couples | 6 | 112 | 4.38 (1.20-16.00) | 0.026 |  |  | 3.89 (1.17-12.90) | 0.027 | 3.70 (1.12-12.20) | 0.032 |
|  |  | Patients with confirmed or suspected STIs and related infections | 13 | 1,669 | 10.00 (5.12-19.50) | <0.001 |  |  | 6.59 (3.30-13.10) | <0.001 | 6.51 (3.28-12.90) | <0.001 |
|  |  | Other populations^c^ | 4 | 4,302 | 0.93 (0.26-3.33) | 0.916 |  |  | 0.82 (0.23-2.95) | 0.763 | 0.80 (0.22-2.85) | 0.729 |
|  | **Age group** | <25 years | 11 | 807 | 1.00 | - | 0.529 | 0.0 | - | - | - | - |
|  |  | 25-34 years | 6 | 505 | 4.02 (0.72-22.30) | 0.112 |  |  | - | - | - | - |
|  |  | 35-44 years | 4 | 400 | 2.09 (0.30-14.50) | 0.456 |  |  | - | - | - | - |
|  |  | ≥45 years | 5 | 193 | 1.74 (0.20-15.30) | 0.615 |  |  | - | - | - | - |
|  |  | Mixed ages | 322 | 110,785 | 1.41 (0.44-4.45) | 0.560 |  |  | - | - | - | - |
|  | **Sex** | Women | 211 | 70,869 | 1.00 | - | <0.001 | 29.5 | 1.00 | - | 1.00 | - |
|  |  | Men | 125 | 39,772 | 5.14 (3.75-7.03) | <0.001 |  |  | 1.69 (1.04-2.73) | 0.033 | 1.73 (1.07-2.79) | 0.025 |
|  |  | Mixed sexes | 12 | 2,049 | 1.20 (0.49-2.95) | 0.684 |  |  | 1.09 (0.48-2.47) | 0.838 | 1.06 (0.47-2.35) | 0.894 |
|  | **MENA Subregions** | Fertile crescent | 109 | 16,834 | 1.00 | - | <0.001 | 7.5 | 1.00 | - | 1.00 | - |
|  |  | Horn of Africa | 23 | 10,904 | 0.64 (0.31-1.34) | 0.240 |  |  | 0.59 (0.35-0.99) | 0.045 | 0.57 (0.34-0.96) | 0.036 |
|  |  | Gulf | 33 | 26,421 | 1.87 (1.01-3.45) | 0.047 |  |  | 1.27 (0.81-2.00) | 0.298 | 1.29 (0.82-2.02) | 0.270 |
|  |  | Maghreb | 44 | 12,155 | 2.01 (1.17-3.46) | 0.011 |  |  | 1.49 (0.99-2.24) | 0.056 | 1.46 (0.97-2.20) | 0.070 |
|  |  | Iran | 96 | 28,675 | 0.59 (0.37-0.94) | 0.025 |  |  | 0.96 (0.67-1.37) | 0.822 | 0.94 (0.67-1.32) | 0.731 |
|  |  | Pakistan and Afghanistan | 40 | 17,701 | 0.68 (0.37-1.25) | 0.219 |  |  | 1.25 (0.76-2.04) | 0.382 | 1.23 (0.75-2.01) | 0.412 |
|  | **National income** | LIC | 9 | 8,819 | 1.00 | - | <0.001^d^ | 6.7 | - | - | - | - |
|  |  | LMIC | 122 | 34,546 | 8.69 (2.65-28.50) | <0.001 |  |  | - | - | - | - |
|  |  | UMIC | 181 | 42,904 | 5.52 (1.69-17.90) | 0.005 |  |  | - | - | - | - |
|  |  | HIC | 36 | 26,421 | 13.30 (3.74-47.60) | <0.001 |  |  | - | - | - | - |
| **Study methodology characteristics** | **Assay type** | NAAT/PCR | 144 | 51,503 | 1.00 | - | <0.001 | 14.4 | 1.00 | - | 1.00 | - |
|  |  | Culture | 117 | 34,795 | 2.08 (1.41-3.07) | <0.001 |  |  | 1.18 (0.86-1.62) | 0.310 | 1.13 (0.82-1.57) | 0.445 |
|  |  | Gram stain | 75 | 21,069 | 4.62 (2.99-7.14) | <0.001 |  |  | 1.96 (1.34-2.86) | 0.001 | 1.87 (1.25-2.79) | 0.002 |
|  |  | Wet mount | 5 | 387 | 1.25 (0.26-6.01) | 0.779 |  |  | 1.20 (0.36-3.96) | 0.763 | 1.17 (0.36-3.84) | 0.798 |
|  |  | Other/unclear | 7 | 4,936 | 2.14 (0.63-7.22) | 0.221 |  |  | 0.89 (0.34-2.33) | 0.817 | 0.83 (0.32-2.19) | 0.708 |
|  | **Sample size** | <200 | 187 | 15,748 | 1.00 | - | <0.001 | 6.1 | 1.00 | - | 1.00 | - |
|  |  | ≥200 | 161 | 96,942 | 0.48 (0.34-0.68) | <0.001 |  |  | 0.41 (0.31-0.54) | <0.001 | 0.39 (0.30-0.51) | <0.001 |
|  | **Sampling method** | Probability based | 42 | 22,262 | 1.00 | - | 0.004 | 2.8 | 1.00 | - | 1.00 | - |
|  |  | Non-probability based | 306 | 90,428 | 2.30 (1.30-4.06) | 0.004 |  |  | 0.65 (0.41-1.04) | 0.075 | 0.63 (0.39-1.01) | 0.056 |
|  | **Response rate** | ≥80% | 170 | 61,786 | 1.00 | - | <0.001 | 6.1 | 1.00 | - | 1.00 | - |
|  |  | <80% | 10 | 4,304 | 0.08 (0.02-0.32) | <0.001 |  |  | 0.14 (0.05-0.42) | 0.001 | 0.14 (0.05-0.41) | <0.001 |
|  |  | Unclear | 168 | 46,600 | 0.55 (0.39-0.78) | 0.001 |  |  | 1.28 (0.95-1.71) | 0.101 | 1.31 (0.98-1.75) | 0.072 |
| **Temporal trend** | **Year of publication category** | <2005 | 110 | 27,849 | 1.00 | - | <0.001 | 13.4 | 1.00 | - | - | - |
|  |  | 2005-2014 | 141 | 59,291 | 0.33 (0.22-0.49) | <0.001 |  |  | 0.71 (0.52-0.96) | 0.027 | - | - |
|  |  | ≥2015 | 97 | 25,550 | 0.28 (0.18-0.43) | <0.001 |  |  | 0.67 (0.45-1.00) | 0.051 | - | - |
|  | **Year of publication** | | 348 | 112,690 | 0.95 (0.94-0.97) | <0.001 | <0.001 | 14.2 | - | - | 0.98 (0.97-1.00) | 0.023 |

Abbreviations: ARR = Adjusted risk ratio, CI = Confidence interval, FSWs = Female sex workers, HIC = High-income country, HIV = Human immunodeficiency virus, MENA = Middle East and North Africa, MSM = Men who have sex with men, MSW = Male sex workers, NAAT = Nucleic acid amplification test, LMIC = Low-middle income country, LT test= Likelihood ratio test, PCR = Polymerase chain reaction, RR = Risk ratio, STI = Sexually transmitted infection, UMIC = Upper-middle income country.

Adjusted R^2^ in the final multivariable model 1 = 64.42%.

Adjusted R^2^ in the final multivariable model 2 = 64.50%.

^a^ Population type classification can be found in Table 1.

^b^ The majority of studies were on male sex workers, primarily from Pakistan, while a smaller proportion of studies were on men who have sex with men.

^c^ Other populations include populations with an undetermined risk of acquiring *Neisseria gonorrhoeae* infection such as victims of sexual assault and mixed populations, among others.

^d^ National income was not included in the multivariable model due to collinearity with MENA subregion variable.

# **Table S8.** Sensitivity analysis. Univariable and multivariable meta-regression analyses for *Neisseria gonorrhoeae* prevalence in urogenital specimens in the Middle East and North Africa (MENA) using national income instead of MENA subregion as the analysis variable.

| **Urogenital specimens** | | | **Stratified prevalence measures** | **Sample size** | **Univariable analysis** | | | | **Multivariable analyses** | | | |
| --- | --- | --- | --- | --- | --- | --- | --- | --- | --- | --- | --- | --- |
|  |  |  | **Total n** | **Total N** | **RR (95% CI)** | **p-value** | **LT test p-value** | **Adjusted R^2^** | **Model 1** | | **Model 2** | |
|  |  |  |  |  |  |  |  |  | **ARR (95% CI)** | **p-value** | **ARR (95% CI)** | **p-value** |
| **Population characteristics** | **Population type^a^** | General populations | 81 | 40,425 | 1.00 | - | <0.001 | 21.3 | 1.00 | - | 1.00 | - |
|  |  | Intermediate-risk populations | 14 | 4,227 | 0.61 (0.27-1.41) | 0.250 |  |  | 0.44 (0.18-1.04) | 0.061 | 0.46 (0.20-1.07) | 0.071 |
|  |  | FSWs | 22 | 7,363 | 2.61 (1.48-4.61) | 0.001 |  |  | 3.57 (2.11-6.04) | <0.001 | 3.26 (1.93-5.53) | <0.001 |
|  |  | MSWs and MSM^b^ | 12 | 2,680 | 1.53 (0.63-3.71) | 0.346 |  |  | 0.84 (0.33-2.17) | 0.722 | 0.86 (0.34-2.20) | 0.759 |
|  |  | Symptomatic women | 78 | 19,502 | 1.65 (1.09-2.49) | 0.017 |  |  | 1.77 (1.20-2.62) | 0.004 | 1.74 (1.18-2.57) | 0.005 |
|  |  | Symptomatic men | 69 | 17,702 | 14.50 (9.80-21.70) | <0.001 |  |  | 6.96 (3.71-13.00) | <0.001 | 6.76 (3.62-12.60) | <0.001 |
|  |  | Symptomatic patients (mixed sexes) | 2 | 263 | 10.30 (2.23-47.50) | 0.003 |  |  | 5.82 (1.41-24.00) | 0.015 | 5.80 (1.42-23.70) | 0.015 |
|  |  | Infertility clinic attendees | 31 | 3,508 | 3.70 (2.09-6.53) | <0.001 |  |  | 2.83 (1.62-4.93) | <0.001 | 2.79 (1.61-4.84) | <0.001 |
|  |  | Women with miscarriage or ectopic pregnancy | 5 | 420 | 1.86 (0.53-6.52) | 0.331 |  |  | 1.59 (0.51-5.01) | 0.425 | 1.48 (0.48-4.59) | 0.494 |
|  |  | STI clinic attendees | 11 | 10,517 | 2.37 (1.13-4.97) | 0.022 |  |  | 3.00 (1.43-6.32) | 0.004 | 3.16 (1.52-6.56) | 0.002 |
|  |  | Individuals living with HIV and individuals in HIV discordant couples | 6 | 112 | 4.38 (1.20-16.00) | 0.026 |  |  | 3.22 (0.98-10.60) | 0.055 | 3.15 (0.96-10.30) | 0.058 |
|  |  | Patients with confirmed or suspected STIs and related infections | 13 | 1,669 | 10.00 (5.12-19.50) | <0.001 |  |  | 6.68 (3.35-13.30) | <0.001 | 6.86 (3.46-13.60) | <0.001 |
|  |  | Other populations^c^ | 4 | 4,302 | 0.93 (0.26-3.33) | 0.916 |  |  | 0.65 (0.18-2.30) | 0.502 | 0.71 (0.20-2.49) | 0.590 |
|  | **Age group** | <25 years | 11 | 807 | 1.00 | - | 0.529 | 0.0 | - | - | - | - |
|  |  | 25-34 years | 6 | 505 | 4.02 (0.72-22.30) | 0.112 |  |  | - | - | - | - |
|  |  | 35-44 years | 4 | 400 | 2.09 (0.30-14.50) | 0.456 |  |  | - | - | - | - |
|  |  | ≥45 years | 5 | 193 | 1.74 (0.20-15.30) | 0.615 |  |  | - | - | - | - |
|  |  | Mixed ages | 322 | 110,785 | 1.41 (0.44-4.45) | 0.560 |  |  | - | - | - | - |
|  | **Sex** | Women | 211 | 70,869 | 1.00 | - | <0.001 | 29.5 | 1.00 | - | 1.00 | - |
|  |  | Men | 125 | 39,772 | 5.14 (3.75-7.03) | <0.001 |  |  | 1.81 (1.12-2.91) | 0.015 | 1.74 (1.08-2.79) | 0.022 |
|  |  | Mixed sexes | 12 | 2,049 | 1.20 (0.49-2.95) | 0.684 |  |  | 0.93 (0.41-2.09) | 0.858 | 0.85 (0.38-1.88) | 0.681 |
|  | **MENA Subregions** | Fertile crescent | 109 | 16,834 | 1.00 | - | <0.001 | 7.5 | - | - | - | - |
|  |  | Horn of Africa | 23 | 10,904 | 0.64 (0.31-1.34) | 0.240 |  |  | - | - | - | - |
|  |  | Gulf | 33 | 26,421 | 1.87 (1.01-3.45) | 0.047 |  |  | - | - | - | - |
|  |  | Maghreb | 44 | 12,155 | 2.01 (1.17-3.46) | 0.011 |  |  | - | - | - | - |
|  |  | Iran | 96 | 28,675 | 0.59 (0.37-0.94) | 0.025 |  |  | - | - | - | - |
|  |  | Pakistan and Afghanistan | 40 | 17,701 | 0.68 (0.37-1.25) | 0.219 |  |  | - | - | - | - |
|  | **National income** | LIC | 9 | 8,819 | 1.00 | - | <0.001 | 6.7 | 1.00 | - | 1.00 | - |
|  |  | LMIC | 122 | 34,546 | 8.69 (2.65-28.50) | <0.001 |  |  | 3.62 (1.55-8.45) | 0.003 | 3.70 (1.61-8.51) | 0.002 |
|  |  | UMIC | 181 | 42,904 | 5.52 (1.69-17.90) | 0.005 |  |  | 3.45 (1.49-8.00) | 0.004 | 3.70 (1.60-8.54) | 0.002 |
|  |  | HIC | 36 | 26,421 | 13.30 (3.74-47.60) | <0.001 |  |  | 3.96 (1.62-9.67) | 0.003 | 4.27 (1.76-10.30) | 0.001 |
| **Study methodology characteristics** | **Assay type** | NAAT/PCR | 144 | 51,503 | 1.00 | - | <0.001 | 14.4 | 1.00 | - | 1.00 | - |
|  |  | Culture | 117 | 34,795 | 2.08 (1.41-3.07) | <0.001 |  |  | 1.06 (0.76-1.47) | 0.745 | 1.00 (0.72-1.38) | 0.996 |
|  |  | Gram stain | 75 | 21,069 | 4.62 (2.99-7.14) | <0.001 |  |  | 1.87 (1.26-2.78) | 0.002 | 1.59 (1.04-2.41) | 0.031 |
|  |  | Wet mount | 5 | 387 | 1.25 (0.26-6.01) | 0.779 |  |  | 0.93 (0.28-3.12) | 0.904 | 0.93 (0.28-3.07) | 0.907 |
|  |  | Other/unclear | 7 | 4,936 | 2.14 (0.63-7.22) | 0.221 |  |  | 0.83 (0.32-2.16) | 0.704 | 0.74 (0.29-1.93) | 0.541 |
|  | **Sample size** | <200 | 187 | 15,748 | 1.00 | - | <0.001 | 6.1 | 1.00 | - | 1.00 | - |
|  |  | ≥200 | 161 | 96,942 | 0.48 (0.34-0.68) | <0.001 |  |  | 0.43 (0.33-0.55) | <0.001 | 0.40 (0.31-0.52) | <0.001 |
|  | **Sampling method** | Probability based | 42 | 22,262 | 1.00 | - | 0.004 | 2.8 | 1.00 | - | 1.00 | - |
|  |  | Non-probability based | 306 | 90,428 | 2.30 (1.30-4.06) | 0.004 |  |  | 0.65 (0.4-1.04) | 0.074 | 0.62 (0.39-1.00) | 0.049 |
|  | **Response rate** | ≥80% | 170 | 61,786 | 1.00 | - | <0.001 | 6.1 | 1.00 | - | 1.00 | - |
|  |  | <80% | 10 | 4,304 | 0.08 (0.02-0.32) | <0.001 |  |  | 0.13 (0.04-0.38) | <0.001 | 0.14 (0.05-0.40) | <0.001 |
|  |  | Unclear | 168 | 46,600 | 0.55 (0.39-0.78) | 0.001 |  |  | 1.23 (0.91-1.66) | 0.170 | 1.25 (0.93-1.68) | 0.131 |
| **Temporal trend** | **Year of data collection category** | <2000 | 102 | 26,032 | 1.00 | - | <0.001 | 11.4 | 1.00 | - | - | - |
|  |  | 2000-2009 | 127 | 54,690 | 0.37 (0.24-0.56) | <0.001 |  |  | 0.90 (0.64-1.26) | 0.537 | - | - |
|  |  | ≥2010 | 119 | 31,968 | 0.30 (0.20-0.46) | <0.001 |  |  | 0.72 (0.48-1.09) | 0.118 | - | - |
|  | **Year of data collection** | | 348 | 112,690 | 0.96 (0.94-0.97) | <0.001 | <0.001 | 14.4 | - | - | 0.98 (0.97-1.00) | 0.013 |

Abbreviations: ARR = Adjusted risk ratio, CI = Confidence interval, FSWs = Female sex workers, HIC = High-income country, HIV = Human immunodeficiency virus, MENA = Middle East and North Africa, MSM = Men who have sex with men, MSW = Male sex workers, NAAT = Nucleic acid amplification test, LMIC = Low-middle income country, LT test= Likelihood ratio test, PCR = Polymerase chain reaction, RR = Risk ratio, STI = Sexually transmitted infection, UMIC = Upper-middle income country.

Adjusted R^2^ in the final multivariable model 1 =63.76%.

Adjusted R^2^ in the final multivariable model 2 =64.38%.

^a^ Population type classification can be found in Table 1.

^b^ The majority of studies were on male sex workers, primarily from Pakistan, while a smaller proportion of studies were on men who have sex with men.

^c^ Other populations include populations with an undetermined risk of acquiring *Neisseria gonorrhoeae* infection such as victims of sexual assault and mixed populations, among others.

# **References**

1. Moher D, Liberati A, Tetzlaff J, Altman DG, Group P: **Preferred reporting items for systematic reviews and meta-analyses: the PRISMA statement**. *J Clin Epidemiol* 2009, **62**(10):1006-1012.

2. Page MJ, McKenzie JE, Bossuyt PM, Boutron I, Hoffmann TC, Mulrow CD, Shamseer L, Tetzlaff JM, Akl EA, Brennan SE *et al*: **The PRISMA 2020 statement: an updated guideline for reporting systematic reviews**. *BMJ* 2021, **372**:n71.

3. **World Bank Country and Lending Groups (Available at:** [**https://datahelpdesk.worldbank.org/knowledgebase/articles/906519-world-bank-country-and-lending-groups**](https://datahelpdesk.worldbank.org/knowledgebase/articles/906519-world-bank-country-and-lending-groups)**. Accessed in June 2017)**

4. Abdollahiyan P, Shodjai Tehrani H, Asghari SH, Oudi M: **Relative frequency of gonococcal endocervisitis and some associated factors in reproductive age women**. *Journal of guilan university of medical sciences* 2005, **14**(55):63-69.

5. Abusarah EA, Awwad ZM, Charvalos E, Shehabi AA: **Molecular detection of potential sexually transmitted pathogens in semen and urine specimens of infertile and fertile males**. *Diagnostic Microbiology and Infectious Disease* 2013, **77(4)**:283-286.

6. Ahmadi A, Mousavi A, Salimizand H, Hedayati MA, Ramazanzadeh R, Farhadifar F, Khodabandehloo M, Roshani D, Taherpour A: **Prevalence of Neisseria gonorrhoeae in Western Iran**. *Jpn J Infect Dis* 2022, **75**(1):1-4.

7. Ahmadnia E, Kharaghani R, Maleki A, Avazeh A, Mazloomzadeh S, Sedaghatpisheh T, Jalilvand A, Molae B: **Prevalence and associated factors of genital and sexually transmitted infections in married women of Iran**. *Oman Medical Journal* 2016, **31(6)**:439-445.

8. Al-Haddad AM: **Urinary tract infection among pregnant women in Al-Mukalla district, Yemen**. *Eastern Mediterranean Health Journal* 2005, **11(3)**:505-510.

9. Al Joubori S, F.: **Lower Genito Urinary Infection in Gynecological Practice.in Baghdad Area**. *Iraqi J Comm Med* 2003, **18**(2):165-172.

10. Al-Mousawi JKN, Tarish HR, Al-Saadi MMK: **Microbiological Study of Bacterial Vaginosis among Pregnant Women in Al-Diwaniya City**. *The Medical Journal of Basrah University* 2006, **24**(1):45-49.

11. Al-Muharrmi Z, Lau R, Al-Balushi A, Al-Saadi A, Al-Habsi Z, Elgalib A, Shah S, Al-Fouri M, Al-Rawahi B, Al-Abri S: **Genitourinary Symptoms Associated with Chlamydia trachomatis and Neisseria gonorrhoeae Infections in a Tertiary Care Hospital in Oman**. *Sultan Qaboos Univ Med J* 2022, **22**(3):382-386.

12. Al-Omar SL, Al-Kaissi NE, Abdulla FS: **Occurrence of HSV with other microorganisms in female genital infection**. *Al-Mustansiriyah Journal for Pharmaceutical Sciences* 2005, **2**(1):1-6.

13. Al-Sweih NA, Khan S, Rotimi VO: **Prevalence of Chlamydia trachomatis and Neisseria gonorrhoeae among asymptomatic women attending the Capital Health region clinics in Kuwait**. *Sexually transmitted diseases* 2011, **38**(9):793-797.

14. Alzahrani AJ, Obeid OE, Hassan MI, Almulhim AA: **Screening of pregnant women attending the antenatal care clinic of a tertiary hospital in eastern Saudi Arabia for Chlamydia trachomatis and Neisseria gonorrhoeae infections**. *Indian Journal of Sexually Transmitted Diseases* 2010, **31(2)**:81-86.

15. Anwar T, Cuevas LE, Shears P: **Neisseria gonorrhoeae infection among pregnant women in Peshawar, Pakistan: Prevalence and risk factors**. *Tropical Doctor* 2000, **30(2)**:81-84.

16. Anwer A, Sultana N: **Prevalance of Sexually Transmitted Diseases among a selected group of Pakistani Women**. *Med Channel* 2001, **7**(4):19-21.

17. As'ad A: **National AIDS Program, Final report sexually transmitted infections (STI) prevalence study, Jordan/2004.** In*.*; 2004.

18. Awad NS, Said MM, Mohamed AA, El-Tarras AE: **Detection of some sexually transmitted bacterial infection using molecular genetic technique**. *World Journal of Medical Sciences* 2013, **9(3)**:142-146.

19. Azizmohammadi S: **Antimicrobial susceptibility pattern of neisseria gonorrhoeae isolated from fertile and infertile Women**. *Tropical Journal of Pharmaceutical Research* 2016, **15(12)**:2653-2657.

20. Baghchesaraei H., Amini B., Hossaini M.: **Prevalence of infection with Neisseria gonorrhoeae and Chlamydia trachomatis in women visitors of gynecology andf obstetrics clinics in Zanjan Province of Iran**. *African Journal of Microbiology* 2011, **5**:2447-2450.

21. Bellaji B, Hancali A, Jennane S, Oukouchoud H, Ouanaim C, Bennani A, Latifi A, Charof R: **Prevalence of chlamydia trachomatis, neisseria gonorrhoeae and trichomonas vaginalis in female sex workers in morocco**. *Sexually Transmitted Infections* 2017, **93(Supplement 2)**:A100.

22. Chaudry AE, Chaudhri R, Kayani A, Hayes LW, Bristow CC, Javaid K, Khan N, Akhlaque S, Yasmeen B, Klausner JD: **Acceptability and feasibility of screening pregnant women for sexually transmitted infections in Rawalpindi, Pakistan**. *Int J STD AIDS* 2021, **32**(10):940-945.

23. Deeb ME, Awwad J, Yeretzian JS, Kaspar HG: **Prevalence of reproductive tract infections, genital prolapse, and obesity in a rural community in Lebanon**. *Bulletin of the World Health Organization* 2003, **81**(9):639-645.

24. Dezfulimanesh M, Tehranian N: **Endocervical gonorrhea in pregnant and non pregnant women and follow up of the infected cases in Kermanshah, Iran 2004**. *Pakistan Journal of Medical Sciences* 2005, **21(3)**:313-317.

25. El-Sayed N, Abdallah M, Abdel Mobdy A, Abdel Sattar A, Aoun E, Beths F, Dallabetta G, Rakha M, Soliman C, Wasef N: **Evaluation of Selected Reproductive Health Infections in Various Egyptian Population Groups in Greater Cairo, MOHP, IMPACT/FHI/USAID. Cairo, Egypt.** 2002.

26. El-sayedAbdou A, Mohamad EEA, Tawfiek AM, Belbasy RE: **Bacterial Infections and Biofilm Formation Associated with Intra Uterine Contraceptive Device among Females Attending Al-Glaa Teaching Hospital in Cairo**. *The Egyptian Journal of Hospital Medicine* 2018, **70**(5):882-890.

27. Esteghamati A, Mazouri A, Sayyahfar S, Khanaliha K, Haghighi F, Faramarzi M, Haghighi Hasanabad M: **Transmission Rates of Chlamydia trachomatis and Neisseria gonorrhoeae Infections from Pregnant Women to Newborns, Tehran, Iran**. *Jundishapur Journal of Microbiology* 2020, **13**(3).

28. Farhan RK: **Common causes of vaginal infections and antibiotic sensitivity of aerobic bacterial isolates in reproductive age women attending Tikrit teaching hospital, Salah al-Din Governorate, Iraq**. *NeuroQuantology* 2022, **20(6)**:7568-7583.

29. Hamze M, Osman M, Achkar M, Mallat H, Dabboussi F: **Alarming increase in prevalence of Neisseria gonorrhoeae infections associated with a high level of antibiotic resistance in Tripoli, Lebanon**. *Int J Antimicrob Agents* 2016, **48**(5):576-577.

30. Hanna J, Yassine R, El-Bikai R, Curran MD, Azar M, Yeretzian J, Skaf R, Afif C, Saber T, Itani S *et al*: **Molecular epidemiology and socio-demographic risk factors of sexually transmitted infections among women in Lebanon**. *BMC Infect Dis* 2020, **20**(1):375.

31. Hassan MK, Al-Shaheen H, Al-Mukh JM: **Bacterial Vaginosis and Preterm Labour**. *The Medical Journal of Basrah University* 2005, **23**(1):42-46.

32. Hassanzadeh P, Mardaneh J, Motamedifar M: **Conventional agar-based culture method and nucleic acid amplification test (NAAT) of the cppB gene for detection of neisseria gonorrhea in pregnant women endocervical swab specimens**. *Iranian Red Crescent Medical Journal* 2013, **15**(3).

33. Ismail SO, Ahmed HJ, Jama MA, Omer K, Omer FM, Brundin M, Olofsson MB, Grillner L, Bygdeman S: **Syphilis, gonorrhoea and genital chlamydial infection in a Somali village**. *Genitourinary Medicine* 1990a, **66(2)**:70-75.

34. Kafi S, Mohamed A, Musa H: **Prevalence of Sexually Transmitted Diseases (STD) Among Women in a Suburban Sudanese Community**. *Upsala journal of medical sciences* 2000, **105**:249-253.

35. Karim S, Bouchikhi C, Banani A, El Fatemi H, Souho T, Erraghay S, Bennani B: **Molecular Antimicrobial Resistance of Neisseria gonorrhoeae in a Moroccan Area**. *Infect Dis Obstet Gynecol* 2018, **2018**:7263849.

36. Karim S, Bouchikhi C, Banani A, Fatemi HE, Souho T, Erraghay S, Bennani B: **Bacterial sexually transmitted infections and syndromic approach: a study conducted on women at Moroccan University Hospital**. *Germs* 2021, **11**(4):544-553.

37. Khalil HI, Al-Kuraishi AH, Al-Naimi UAM, Al-Naimi SA: **Trichomoniasis Vaginalis in Women Attending Family Planning Unit in AL-Liqa'a Hospital داء المشعرات المهبلية لدى النساء المراجعات لوحدة تنظيم الاسرة في مستشفى اللقاء**. *Iraqi Journal of Science* 2012, **53**(4):746-653.

38. Khoder M, Osman M, Diene SM, Okdah L, Lalaoui R, Al Achkar M, Mallat H, Hamze M, Rolain JM: **Evaluation of different testing tools for the identification of non-gonococcal Neisseria spp. isolated from Lebanese male semen: a strong and significant association with infertility**. *J Med Microbiol* 2019, **68**(7):1012-1020.

39. Latif AS, Magtooph MG, Raheem IA: **Relationship between lead contaminations with the cervical inflammatory in iraqi women of baghdad**. *Biochemical and Cellular Archives* 2020, **20(Supplement2)**:4287-4293.

40. Lau R, Eskander R, Al-Yaqoobi M, Al-Habsi Z, Elgalib A, Shah S, Al-Fouri M, Al-Rawahi B, Al-Abri S: **Acceptability and feasibility of testing for sexually transmitted infections among pregnant women in Muscat, Sultanate of Oman**. *Int J STD AIDS* 2021, **32**(9):816-820.

41. Mahafzah AM, Al-Ramahi MQ, Asagd AM, El-Khateeb MS: **Prevalence of sexually transmitted infections among sexually active Jordanian females**. *Sexually Transmitted Diseases* 2008, **35(6)**:607-610.

42. Mir AM, Wajid A, Reichenbach L, Khan M: **STI prevalence and associated factors among urban men in Pakistan**. *Sexually Transmitted Infections* 2009, **85(3)**:199-200.

43. Ministry of Health and Medical Education and HIV/STI Office: **Situation Analysis of Sexually Transmitted Infections in the Islamic Republic of Iran**. In*.* Tehran, Iran; 2008.

44. Jordan Ministry of Health: **Prevalence of reproductive tract infections in women attending selected urban OB/GYN clincis in Jordan**. In*.*; 2004.

45. Ministry of Health - Morocco: **Etude de prévalence IST chez les femmes consultantes en SMI/PF à la Wilaya de Rabat, Rapport final, Programme National de lutte contre les IST/SIDA.** In*.* Rabat, Morocco; 2001.

46. Mortazavi SM, Tarinjoo A, Dastani S, Niyazpour M, Dahaghin S, Mirnejad R: **Molecular Detection of Sexually Transmitted Infections in Women with and without Human Papillomaviruses Infection Who Referred to Tehran West Hospitals in Iran**. *Reports of Biochemistry & Molecular Biology* 2021, **10**(3):387.

47. Motamedifar M, Malekzadegan Y, Namdari P, Dehghani B, Jahromi BN, Sarvari J: **The Prevalence of Bacteriospermia in Infertile Men and Association with Semen Quality in Southwestern Iran**. *Infect Disord Drug Targets* 2020, **20**(2):198-202.

48. Nateghi Rostami M, Hossein Rashidi B, Habibi A, Nazari R, Dolati M: **Genital infections and reproductive complications associated with Trichomonas vaginalis, Neisseria gonorrhoeae, and Streptococcus agalactiae in women of Qom, central Iran**. *Int J Reprod Biomed* 2017, **15**(6):357-366.

49. Ortashi OM, El Khidir I, Herieka E: **Prevalence of HIV, syphilis, Chlamydia trachomatis, Neisseria gonorrhoea, Trichomonas vaginalis and candidiasis among pregnant women attending an antenatal clinic in Khartoum, Sudan**. *Journal of Obstetrics and Gynaecology* 2004, **24(5)**:513-515.

50. Pourabbas B, Rezaei Z, Mardaneh J, Shahian M, Alborzi A: **Prevalence of Chlamydia trachomatis and Neisseria gonorrhoeae infections among pregnant women and eye colonization of their neonates at birth time, Shiraz, Southern Iran**. *BMC Infect Dis* 2018, **18**(1):477.

51. Rashidi B, Chamani Tabriz L, Hagh Elahi F, Jedi Tehrani M, Ramazanzadeh F, Rahimi Foroushani A, Shariat M, Akhoundi MM, Daneshjou F: **Prevalewnce of Neisseria gonorrhea in fertile and infertile women in Tehran**. *Journal of Reproduction and Infertility* 2009, **9**(4 (37)):379-383.

52. Sameni F, Zadehmodarres S, Dabiri H, Khaledi M, Nezamzadeh F: **Evaluation of Ureaplasma urealyticum, Chlamydia trachomatis, Mycoplasma genitalium and Neisseria gonorrhoeae in infertile women compared to pregnant women**. *J Obstet Gynaecol* 2022:1-5.

53. Shaaban HA: **Prevalence of chlamydial and gonococcal infections in infertile Egyptian women with tubal factor**. *Medical Journal of Cairo University [The]* 1994, **62**(2):375-385.

54. WHO: **Central South: The 2004 First National Second Generation HIV/AIDS/STI Sentinel Surveillance Survey**. 2005a.

55. Abdelrahim NA, Ahmed HI, Fadl-Elmula IM, Bayoumi MA, Homeida MM: **Sexually transmitted infections other than HIV/AIDS among women of low socio-economic class attending antenatal clinics in Khartoum, Sudan**. *International Journal of STD and AIDS* 2017, **28(8)**:781-787.

56. Altaf A, Janjua NZ, Kristensen S, Zaidi NA, Memon A, Hook IEW, Vermund SH, Shah SA: **High-risk behaviours among juvenile prison inmates in Pakistan**. *Public Health* 2009, **123(7)**:470-475.

57. Faisel A, Cleland J: **Migrant men: A priority for HIV control in Pakistan?** *Sexually Transmitted Infections* 2006, **82(4)**:307-310.

58. Ghanbarzadeh N, Nadjafi-Semnani M: **A Study of HIV and other sexually transmitted infections among female prisoners in Birjand**. *Journal of Birjand University of Medical Sciences* 2006, **13**(3):9-15.

59. Mousaviani ZAS, Esmaeili I, Behbahani SMR: **Diagnosing contamination and determining effective factors on contraction of trichomonas vaginalis and gonorrhea in female prisoners at Evin jail Tehran, 2004**. *Researcher Bulletin of Medical Sciences* 2005, **9**(5 (41)):301-303.

60. Platt L, Vickerman P, Collumbien M, Hasan S, Lalji N, Mayhew S, Muzaffar R, Andreasen A, Hawkes S: **Prevalence of HIV, HCV and sexually transmitted infections among injecting drug users in Rawalpindi and Abbottabad, Pakistan: evidence for an emerging injection-related HIV epidemic**. *Sex Transm Infect* 2009, **85 Suppl 2**:ii17-22.

61. Rehan N: **Profile of men suffering from sexually transmitted infections in Pakistan**. *Journal of Ayub Medical College, Abbottabad : JAMC* 2003, **15(2)**:15-19.

62. Shahcheraghi F, Shafiei M, Valadkhani Z: **Detection of Neisseria gonorrhoeae from vaginal swabs of Ewin, Rajaii shahr, Karaj and Varamin female prisoners by PCR and culture methods**. *Pakistan Journal of Biological Sciences* 2010, **13(4)**:198-200.

63. Valadkhani Z, Shahcheraghi F, Shafiei M, Hassan N, Aghighi Z, Kazemi F: **Detection of Trichomonas vaginalis and Neisseria gonorrhoeae from vaginal discharge of women attended in gynecology clinics**. *International Journal of Infectious Diseases* 2010, **14**:S83.

64. Hawkes S, Collumbien M, Platt L, Lalji N, Rizvi N, Andreasen A, Chow J, Muzaffar R, Ur-Rehman H, Siddiqui N *et al*: **HIV and other sexually transmitted infections among men, transgenders and women selling sex in two cities in Pakistan: A cross-sectional prevalence survey**. *Sexually Transmitted Infections* 2009, **85(SUPPL. 2)**:ii8-ii16.

65. Kazerooni PA, Motazedian N, Motamedifar M, Sayadi M, Sabet M, Lari MA, Kamali K: **The prevalence of human immunodeficiency virus and sexually transmitted infections among female sex workers in Shiraz, South of Iran: By respondent-driven sampling**. *International Journal of STD and AIDS* 2014, **25(2)**:155-161.

66. Khan MS, Unemo M, Zaman S, Lundborg CS: **HIV, STI prevalence and risk behaviours among women selling sex in Lahore, Pakistan**. *BMC Infectious Diseases* 2011, **11 (no pagination)**.

67. Khezri M, Shokoohi M, Mirzazadeh A, Karamouzian M, Sharifi H, Haghdoost A, Baral SD: **Early sex work initiation and its association with condomless sex and sexually transmitted infections among female sex workers in Iran**. *Int J STD AIDS* 2020, **31**(7):671-679.

68. Mirzazadeh A, Shokoohi M, Karamouzian M, Ashki H, Khajehkazemi R, Salari A, Abedinzadeh N, Nadji SA, Sharifi H, Kazerooni PA *et al*: **Declining trends in HIV and other sexually transmitted infections among female sex workers in Iran could be attributable to reduced drug injection: A cross-sectional study**. *Sexually Transmitted Infections* 2020, **96(1)**:68-75.

69. Ministry of Health - Morocco: **Etude de prévalence des IST chez les femmes qui consultent pour pertes vaginales et/ou douleurs du bas ventre**. In*.* Rabat, Morocco; 2008.

70. Ministry of Health - Morocco: **HIV integrated behavioral and biological surveillance surveys - Morocco 2011**. In*.*; 2011.

71. Nasirian M, Kianersi S, Hoseini SG, Kassaian N, Yaran M, Shoaei P, Ataei B, Fadaei R, Meshkati M, Naeini AE *et al*: **Prevalence of Sexually Transmitted Infections and Their Risk Factors among Female Sex Workers in Isfahan, Iran: A Cross-Sectional Study**. *Journal of the International Association of Providers of AIDS Care* 2017, **16(6)**:608-614.

72. Zirak-Zadah T, Delavarian H, Bahavar MA, Majidi V, Yaminifar R, Masoumi P: **Penicillin-resistant strains of Neisseria gonorrhoeae in Shahre-Now**. *Trop Doct* 1977, **7**(2):57-58.

73. Znazen A, Frikha-Gargouri O, Berrajah L, Bellalouna S, Hakim H, Gueddana N, Hammami A: **Sexually transmitted infections among female sex workers in Tunisia: High prevalence of Chlamydia trachomatis**. *Sexually Transmitted Infections* 2010, **86(7)**:500-505.

74. Ministry of Health - Morocco: **Integrated behavioral and biological surveillance survey among men who have sex with men (MSM) in Agadir, Casablanca, Marrakech, and Tangier, Morocco 2015**. In*.*; 2015.

75. Afrakhteh M, Mahdavi A, Beyhaghi H, Moradi A, Gity S, Zafargandi S, Zonoubi Z: **The prevalence of Chlamydia trachomatis in patients who remained symptomatic after completion of sexually transmitted infection treatment**. *Iranian journal of reproductive medicine* 2013, **11**(4):285.

76. Afrasiabi S, Moniri R, Samimi M, Mousavi SG: **The frequency of Neisseria gonorrhoeae endocervical infection among female carrier and changing trends of antimicrobial susceptibility patterns in Kashan, Iran**. *Iranian Journal of Microbiology* 2014, **6(3)**:194-197.

77. Akya A, Hosseini M, Olfati M, Mirnejad R, Altaha SM, Rezaee M: **The frequency of Chlamydia trachomatis and Neisseria gonorrhoeae infections among women in Kermanshah, Iran**. *Asian Biomedicine* 2013, **7(5)**:681-685.

78. Al Habib HM, Al Dabbagh NY, Al Daheen GA: **The prevalence of trichomonans vaginalis in association with other micro-organisms among women with vaginal discharge in Mosul**. *Ann Coll Med Mosul* 2005, **31**(1):37-44.

79. Al Kaisi AAR, Al Janabi B, Al Tikriti R: **A study on the common microorganisms causing vaginitis**. *Iraqi Postgrad Med J* 2006, **5**(4):426-430.

80. Al Quaiz JM: **Patients with vaginal discharge: A survey in a university primary care clinic in Riyadh city**. *Annals of Saudi Medicine* 2000, **20(3-4)**:302-306.

81. Ali MK, Shia JS: **Detection of Chlamydia trachomatis and Neisseria gonorrhoeae in genitourinary specimens in Iraq women by real time PCR assay**. *Research Journal of Pharmaceutical, Biological and Chemical Sciences* 2018, **9(6)**:799-808.

82. Al-Muqdadi SF, Mhaisen FT, Al-Tae AA: **Distribution of the Infection with Trichomonas vaginalis and Associated Microorganisms in Women Attending Two Hospitals in Al-Sader City, Baghdad**. *Ibn Al-Haitham Journal For Pure and Applied Sciences* 2010, **23**(1):19-25.

83. Al-Mutairi N, Joshi A, Nour-Eldin O, Sharma AK, El-Adawy I, Rijhwani M: **Clinical patterns of sexually transmitted diseases, associated sociodemographic characteristics, and sexual practices in the Farwaniya region of Kuwait**. *International Journal of Dermatology* 2007, **46(6)**:594-599.

84. Arif S, Hanna KM, El Kholy m: **Efficacy of an enzyme immunoassay for detection of gonorrhea in males and females**. *Medical Journal of Cairo University* 1989, **57**:31-38.

85. Azizi S: **Pathogens associated with female genital tract infections in Kabul**. *Virchows Archiv* 2017, **471(1 Supplement 1)**:S99.

86. Bakhshi A, Safayi Delouyi Z, Taheri S, Alivandi A, Mohammadzadeh N, Dabiri H: **Comparative study of lactobacilli and bifidobacteria in vaginal tract of individual with bacterial vaginosis and healthy control by quantitative PCR**. *Reviews in Medical Microbiology* 2019, **30**(3):148-154.

87. Bakhtiari A, Rahmani Firouzjaei A: **The prevalence of gonococcal infection in non pregnant women**. *Iranian Journal of Public Health* 2007, **36**(2):64-67.

88. Douaa Hamza K-A, Mohammad SA-R, Asmaa KG: **Detection of Phospholipase Enzyme in Bacterial Associated with Vaginitis**. *Medical Journal of Babylon* 2013, **10**(4):950-955.

89. Ekhlas Mushref د. اخلاص مشرف ع, Naksheen MAمنمم, Zahra’a Abdul-Raheem Ahmed م.م. زهراء عبد الرحيم ا: **Trichomonas vaginalis used as a marker for other sexually transmitted infections in women إمكانية استخدام طفيلي المشعرات المهبليه كدليل لأصابات جنسية اخرى عند النساء**. *Iraqi Journal of Community Medicine* 2010, **23**(4):292-294.

90. Elkayal NM, Mahmoud NF, Abdalla S: **Detection of Chlamydia trachomatis and Neisseria gonorrhoeae in Egyptian women suffering from infertility**. *Advances in Microbiology* 2015, **5**(12):769-779.

91. Faroughi E, Amini K: **Molecular identification of Neisseria gonorrhoeae and Toxoplasma gondii isolated from infertile women with vaginal swab samples by Multiplex-PCR**. *Alborz University Medical Journal* 2021, **10**(3 #HD00299):297-304.

92. Farraj MA, Abusada GM, Saleem AAM, Joaidi AY, Radad RM, Atrash HN, Sabri IN, Essawi TA: **Detection of Neisseria gonorrhoeae in Palestinian women using polymerase chain reaction**. *Asian Biomedicine* 2010, **4(4)**:637-640.

93. Ghotbi S, Beheshti M, Amirizade S: **Causes of Leukorrhea in Fasa, Southern Iran**. 2007, **8**(2):58-63.

94. Gul F, Faiz NR, Raziq F, Malik L, Sherin A, Kazi BM, Bukhari A: **Frequency of vaginal discharge and its association with various sexually transmitted diseases in women attending antenatal clinic**. *Journal of Postgraduate Medical Institute* 2005, **19(1)**:86-91.

95. Haddadian M, Agholi M, Kroup M, Motazedian MH, Ebrahimii E: **Prevalence of specific cervicitis among women with inflammatory cervix in Shiraz, south of Iran**. *Tropical Medicine and International Health* 2011, **1)**:153-154.

96. Jaafar NK, Kadhum TJ, Ismael I: **Study Of Causative Agents Of Cervicitis In Women Attending Gynecologic Outpatient Department In Najaf City دراسة مسببات التهاب عنق الرحم لدى النساء الوافدات إلى قسم النسائية في مستشفيات محافظة النجف**. *Kufa Medical Journal* 2008, **11**(1):166-174.

97. Kadir M, Aziz LJ: **A study on trichomonas vaginals infection in Kirkur-Iraq**. *Bulletin of Endemic Disease-Baghdad* 1989, **30**:1-8.

98. Kareem HK, Hamad MM, Hasan MA, Abd alsammed MA: **Study on Trichomonas vaginalis infection in women with type-2 diabetes mellitus and vaginal discharge in Thi-Qar Government**. *European Journal of Molecular and Clinical Medicine* 2020, **7**(8):4471-4478.

99. Kazemian H, Zarandi MK, Zargoush Z, Ghafourian S, Sadeghifard N, Jalilian A, Shafieian M, Pakzad I: **The prevalence of gonococcal and non-gonococcal infections in women referred to obstetrics and gynecology clinics**. *Infez Med* 2022, **30**(2):247-253.

100. Luni Y, Munim S, Qureshi R, Tareen AL: **Frequency and diagnosis of bacterial vaginosis**. *J Coll Physicians Surg Pak* 2005, **15**(5):270-272.

101. Mahdi NK, Al Hamdani MM: **Sexually transmitted diseases among women with habitual abortion**. In*.*; 1998.

102. Mehrabani D, Behzadi MA, Azizi S, Payombarnia H, Vahdani A, Namayandeh M, Ziyaeyan M: **Cervical infection with herpes simplex virus, Chlamydia trachomatis, and Neisseria gonorrhoeae among symptomatic women, Dubai, UAE: A molecular approach**. *Interdisciplinary Perspectives on Infectious Diseases* 2014, **2014 (no pagination)**.

103. Moaiedmohseni S, Bashardoost L, Abbasi M: **Cervicovaginal infections during third trimester of pregnancy**. *Journal Of Family and Reproductive Health* 2012:11-15.

104. Mohseni R, Sadeghi F, Miri Nargesi M, Eghbali M, Dezhkame S, Ghane M: **A study on the frequency of vaginal species of mycoplasma genitalium, gardnerella vaginalis and neisseria gonorrhoeae among pregnant women by PCR technique**. *International Journal of Molecular and Clinical Microbiology* 2013, **3**(1):231-236.

105. Molaei B, Mohmmadian F, Eftekhar M, Hatami R, Tirkan A, Kiani M: **The frequency of gonorrheal and chlamydial infections in zanjanian women in 2013-2014**. *International Journal of Reproductive BioMedicine* 2017, **15(2)**:75-82.

106. Mozher HM: **عزل وتشخيص بعض انواع الاحياء المجهرية المسببة لالتهابات المهبل المرافقة لاصابات القناة التناسلية الانثوية ودراسة تأثير بعض العوامل على انتشارها**. *Journal of Education for Pure Science* 2011, **1**(4):147-157.

107. Omer EE, Ali MH, Erwa HH: **Study of sexually transmitted disease in Sudanese women**. *Tropical doctor* 1980, **10(3)**:99-102.

108. Omer EFEO, El-Naeem HAR, Ali MH: **Micro-organisms associated with vaginal trichomoniasis among Sudanese women**. *Saudi Medical Journal* 1985, **6(2)**:129-134.

109. Rahimi MK, Zakerbostanabadi S, Mirfakhraei M, ADIMI NP, Bossak M, Masoomi M, Taiebi Z, Behroznasab K, Omidian M: **Evaluation of antibiotic resistance of gonococcal infections in women referred to Azad University hospitals**. *Medical Science Journal of Islamic Azad Univesity-Tehran Medical Branch* 2011, **21**(1):50-54.

110. Rajabpour M, Emamie AD, Pourmand MR, Goodarzi NN, Asbagh FA, Whiley DM: **Chlamydia trachomatis, Neisseria gonorrhoeae, and Trichomonas vaginalis among women with genitourinary infection and pregnancy-related complications in Tehran: A cross-sectional study**. *Int J STD AIDS* 2020, **31**(8):773-780.

111. Ryan CA, Zidouh A, Manhart LE, Selka R, Xia M, Moloney-Kitts M, Mahjour J, Krone M, Courtois BN, Dallabetta G *et al*: **Reproductive tract infections in primary healthcare, family planning, and dermatovenereology clinics: Evaluation of syndromic management in Morocco**. *Sexually Transmitted Infections* 1998, **74(SUPPL. 1)**:S95-S105.

112. Sadiq AM, Yousif MG: **Vaginal leucocyte counts as indicator for cervical infections in women with bacterial vaginosis in Najaf, Iraq**. *Kufa Medical Journal* 2008, **11**(1):110-120.

113. Saleh AAM, Altooky MH, Elkady AA, Azab HS, Elaaser EM: **The microbiology of vaginal discharge and the prevalence of bacterial vaginosis in a cohort of non-pregnant women in Kuwait**. *Kuwait Medical Journal* 2012, **44(1)**:20-25.

114. Sallam S, Ali OT, Hassan MN, Fares E: **Epidemiology of gonorrhea among married females presenting with leucorrhoea**. *Bulletin of High Institute of Public Health* 1982, **12**(3):65-80.

115. Sami S, Baloch SN: **Vaginitis and sexually transmitted infections in a hospital based study**. *J Pak Med Assoc* 2005, **55**(6):242-244.

116. Shakibaei MR, Ardebili A, Aali SH, Ketabchi AA, Shahabinezhad N: **Antibiotic resistance, β-lactamase production and plasmid profile of Neisseria gonorrhoeae strains isolated from urethritis and cervicitis patients in Kerman, Iran**. *Medical Journal Of Tabriz University Of Medical Sciences* 2008, **30**(3):61-66.

117. Tabasi Z, Khourshidi A, Ali Naghipour M, Sadat Z, Akbari H: **Prevalence of Neisseria gonorrhoeae in cervisitis and evaluation of drug resistance of N.gonorrhoeae in Kashan**. *Feyz* 2002, **6**(22):70-74.

118. Torabizadeh R, Eslami G, Dehghantarzejani MH, Zahirnia Z, Habibi M: **The prevalence of neisseria gonoroheae among iranian women by phenotyping and genotyping methods**. *Journal of Pure and Applied Microbiology* 2016, **10(4)**:2669-2672.

119. Yaseen SAS: **Study About the Causative Agents of Cervical Infections and Cytopathological Changes in Iraqi Women**. *Iraqi Journal of Science* 2020, **61**(2):246-253.

120. Znazen A, Zribi N, Maazoun L, Khrouf S, Hammami A: **Epidemiological features of sexually transmitted infections among women in Tunisia: High prevalence of chlamydia trachomatis among women requesting abortion**. *Sexually Transmitted Infections* 2013, **89(1)**:56.

121. Zolfaghari P, Emamie AD, Rajabpour M, Zarei A, Whiley DM, Pourmand MR, Pourmand G: **Antimicrobial susceptibility testing and molecular characterization of Neisseria gonorrhoeae in Tehran, Iran**. *Int J STD AIDS* 2022, **33**(7):660-665.

122. Zribi M, Ben Mansour K, Abid F, Masmoudi A, Fendri C: **Syndromic approach to sexually transmitted infections in Tunisian women: Bacteriological validation**. *International Journal of STD and AIDS* 2008, **19(2)**:112-114.

123. Akhi MT, E E, Amjadi M: **The role of ureaplasma urealyticum in male non-gonococcal urethritis in Tabriz**. *Rawal Med J* 2009, **34**(1):65-67.

124. Alami K, Mbarek Ait N, Akrim M, Bellaji B, Hansali A, Khattabi H, Sekkat A, El Aouad R, Mahjour J: **Urethral discharge in Morroco: Prevalence of microorganisms and susceptibility of gonococcos**. *Eastern Mediterranean Health Journal* 2002, **8(6)**:794-804.

125. Alavi SM, Soltani MH: **Study on urethritis and determination of its risk factors in male patients attending infectious disease clinic in Ahvaz, 2005-2007**. *Jundishapur Scientific Medical Journal* 2009, **8**(3 (62)):265-573.

126. Al-Hattawi K, Ison CA: **Characteristics of gonococci isolated from men with urethritis in Dubai**. *Epidemiology and Infection* 1996, **116(1)**:15-20.

127. Ali Miknas A: **Miscellaneous bacterial prostatic infections**. *Journal of Techniques* 2008, **21**(3):105-109.

128. Al-Jawamis F, Haddadin J: **Penicillinase-producing Neisseria gonorrhoeae in Zarka City of Jordan**. *Jordan Medical Journal* 1991, **25(1)**:71-77+128.

129. Al-Sweih N, Khan S, Rotimi V: **The prevalence of Chlamydia trachomatis and Neisseria gonorrhoeae infections among men with urethritis in Kuwait**. *Journal of Infection and Public Health* 2011, **4**(4):175-179.

130. Amin M, Emara A, Bader H, Elghandour T: **Detection of Neisseria Gonorrhea, Chlamydia trachomatis and Mycoplasma Genitalium in Acute Male Urethritis Using Multiplex PCR**. *Egypt J Med Lab Sci, Sept* 2007, **16**:65-76.

131. Aziz A, Mark R, Bindayna K: **Male urethritis in Bahrain: the increasing incidence of resistant gonorrhea**. 1991.

132. Baaj AJ, Nejmi S, Khaldi M, Sekkat A: **Acute uretritis in military collectivity**. 1980.

133. Devrajani BR, Bajaj DR, Shah SZA, Ghori RA: **Frequency and pattern of gonorrhoea at Liaquat University Hospital, Hyderabad (a hospital based descriptive study)**. *Journal of the Pakistan Medical Association* 2010, **60(1)**:37-40.

134. El Gamal S, Awad RA: **Comparison of microscopy and culture for diagnosis of gonorrhea in males**. *Journal of the Egyptian Medical Association* 1988, **71**(9-12):633-641.

135. El Ghazzawy I: **Use of urinary leukocyte esterase screening test for asymptomatic chlamydial and gonococcal infections in young men**. *El Ghazzawy, I* 1993, **29**(5):1101-1104.

136. Elghoul MT, Joshi RM: **Antimicrobial susceptibility of non-penicillinase and penicillinase-producing Neisseria gonorrhoeae strains isolated in Tripoli, Libya**. *Int J STD AIDS* 1990, **1**(5):343-345.

137. Ezz Eddin SM, Sorour MA: **Sexually transmitted urethritis in male patients: a clinical bacteriological study**. *Journal of the Egyptian Medical Association [The]* 1992, **75**(1-6):1-10.

138. Farooq M, U BA, Sheikh ZI, farooq M: **Urethritis in men: evaluation of risk factors and aetiological pathogens among our population**. *J Pak Assoc Derma* 2007, **17**(4):219-224.

139. Fath Elahzadeh B, Mirsalehian A, Kazemi B, Arshadi H, Pourakbari B: **Detection of chlamydia trachomatis and Neisseria gonorrhoae by PCR and multiplex PCR from non-invasive genito-urinary specimen of patients with urethritis**. *Tehran University Medical Journal* 2004, **62**(6):449-456.

140. Haberberger RL, Jr., Mikhail IA, Fox E, Bailly C, Salah-Abdillahi W, Polycarpe D, Abbatte EA: **Predominance of vancomycin-sensitive strains of Neisseria gonorrhoeae in Djibouti**. *Lancet* 1989, **2**(8664):683.

141. Hancali A, Ndowa F, Bellaji B, Bennani A, Kettani A, Charof R, El Aouad R: **Antimicrobial resistance monitoring in Neisseria gonorrhoeae and strategic use of funds from the Global Fund to set up a systematic Moroccan gonococcal antimicrobial surveillance programme**. *Sex Transm Infect* 2013, **89 Suppl 4**:iv24-27.

142. Heidari M, Kheirollahi A, Nazer M, Birjandi M, Zareie H: **Frequency of epididymo-orchitis in hospitalized patients with acute scrotum at Shohadaye Ashayer Hospital, Khorramabad, Iran**. *Journal of the Pakistan Medical Association* 2012, **62**(1).

143. Isam Yousif M: **Prevalence of gonorrhea among adult male with urethritis in Erbil City انتشار السيلان بين الذكور البالغين المصابين بالتهاب الإحليل في مدينة أربيل**. *Zanco Journal of Medical Sciences* 2014, **18**(2):692-696.

144. Ismail SO, Ahmed HJ, Grillner L, Hederstedt Issa BA, Bygdeman S: **Sexually transmitted diseases in men in Mogadishu, Somalia**. *International Journal of STD and AIDS* 1990, **1(2)**:102-106.

145. Mahgoub F, Khidir I, Kordofani Y, Shamad M: **Proportion of sexually transmitted infections in patients attending the out-patient clinic in Omdurman military hospital**. *Sudanese Journal of Dermatology* 2005, **3**(1).

146. Massenet D, Aboubakar AB: **Disappearance of vancomycin-sensitive strains of Neisseria gonorrhoeae in Djibouti**. *Acta Tropica* 1999, **72(3)**:317-318.

147. Mohammed M, Y., Al-Mashhadani S, Al-Waiz M, M.: **The frequency of chlamydial urethritis among a group of Iraqi male patients**. *Iraqi Journal of Community Medicine* 2007, **20**(2):354-359.

148. Naderinasab M, Aghaie MA, Ataei R, Tajzadeh P, Norozi HR: **A report on patients who were suspected gonorrhea and refereed to the clinical laboratories of Mashhad**. *Koomesh* 2009, **10(3)**:191-195+128.

149. Pareek SS, Chowdhury MNH: **Sexually transmitted diseases in Riyadh, Saudi Arabia. A study of patients attending a teaching hospital clinic**. *British Journal of Venereal Diseases* 1981, **57**(5):343-345.

150. Sabri M, Salih M: **Investigation on Some Ethological Agent Associated with Male Urethritis**. *Medical Journal of Babylon* 2007, **4**(1-2):49-52.

151. Saleem K, Mumtaz B, Raza N: **A clinicopathological study of urethritis in males**. *J Coll Physicians Surg Pak* 2009, **19**(12):772-775.

152. Sellami A, Kharfi M, Youssef S, Zghal M, Fazaa B, Mokhtar I, Kamoun MR: **[Epidemiologic profile of sexually transmitted diseases (STD) through a specialized consultation of STD]**. *Tunis Med* 2003, **81**(3):162-166.

153. Soleimani Rahbar AA, Niakan M, Fayaz F, Taheri S, Mahmoudian J, Nejadmoghaddam MR, Kolahi AA: **Crystal Violet in Culture Media for Diagnosis of Neisseria Species**. *Research-in-Medicine* 2008, **32**(3):201-206.

154. Waseem H, Naeem A, Ali S, Sarfraz S, Usman J, Gilani M: **Multiplex polymerase chain reaction (PCR) for the detection of neisseria gonorrhoeae and the quinolone resistance gene in Pakistan**. *Pak Armed Forces Med J* 2021, **71**(3):866-869.

155. Zabaneh H, Bacos M, Nassar NT, Alami SY, Kurban AK: **Gonorrhea in the male: some aspects of treatment in Lebanon**. *Le Journal medical libanais* 1980, **31**(1):89-93.

156. Znazen A, Trigui B, Zghal-Trigui Y, Gdoura R, Zouari N, Hammami A: **The male gonococcal urethritis in the area of Sfax(1996-2000). [French]**. *Tunisie Medicale* 2003, **81(5)**:329-333.

157. Ali F, Aziz AA, Helmy MF, Mobdy AA, Darwish M: **Prevalence of certain sexually transmitted diseases in Egypt**. *J Egypt Public Health Assoc* 1996, **71**(5-6):553-575.

158. Tsai AY, Dueger E, Macalino GE, Montano SM, Tilley DH, Mbuchi M, Wurapa EK, Saylors K, Duplessis CC, Puplampu N *et al*: **The U.S. military's Neisseria gonorrhoeae resistance surveillance initiatives in selected populations of five countries**. *MSMR* 2013, **20**(2):25-27.

159. Abu Khanjar RH, Al-Azawi IH: **Diagnosis of Neisseria gonorrhea Using Molecular Method in Infertile Iraqi Male**. *International Journal of Drug Delivery Technology* 2022, **12(1)**:361-365.

160. AL-Douri IM, Abood R, Mukleaf AA, Hoom KI: **Women's infertility with gonorrhea infection in Baghdad**. *Journal of Techniques* 2008, **21**(3):33-38.

161. Al-Hadrawi KKA, Al- Kaabi SJM, Al-Saimary IE: **Study of bacterial species associated with cases of infertility in men in the province of Najaf. دراسة الانواع البكتيرية المرافقة لحالات العقم لدى الرجال في محافظة النجف الاشرف**. *Al-Kufa University Journal for Biology* 2015, **7**(2):29-42.

162. Al-Janabi A, Jubair A, Pemmaraju S, Pruthi P, Pruthi V: **The role of bacterial infections on male infertility in Al-Anbar province of Iraq**. *Int J Med Sci Public Health* 2014, **3**(2):177-180.

163. Bakir TM, Hossain A, De-Silva S, Siddiqui A, Sengupta BS, el-Sheikh MM, Bakir AF: **Enzyme immunoassay in the diagnosis of Chlamydia trachomatis infections in diverse patient groups**. *Journal of hygiene, epidemiology, microbiology, and immunology* 1989, **33(2)**:189-197.

164. Farooq Faisal S, Adnan Abdul Hameed W, Alwasiti E: **The Influence of Vaginal Dysbiosis on Intracytoplasmic Sperm Injection Outcome**. *Archives of Razi Institute* 2023, **78**(1):221-226.

165. Gdoura R, Kchaou W, Znazen A, Chakroun N, Fourati M, Ammar‐Keskes L, Hammami A: **Screening for bacterial pathogens in semen samples from infertile men with and without leukocytospermia**. *Andrologia* 2008, **40**(4):209-218.

166. Golshani M, Taheri S, Eslami G, Suleimani Rahbar AA, Fallah F, Goudarzi H: **Genital tract infection in asymptomatic infertile men and its effect on semen quality**. *Iranian Journal of Public Health* 2006, **35(3)**:81-84.

167. Ramezani M, Zainodini N, Rahnama A, Sayadi A: **Survey on the Prevalence of Chlamydia trachomatis and Neisseria gonorrhoeae Infections and Their Possible Effects on Seminal Quality in Infertile Men**. *INTERNATIONAL JOURNAL OF INFECTION* 2019, **6**(4):0-0.

168. Rehab Sh Al-Maliki رحاب شفيق ا: **Effect of aqueous extract of turmeric on pathogenic bacteria isolated from semen in a sample of Iraqi infertile men**. *Iraqi Journal of Medical Sciences* 2012, **10**(2):105-110.

169. Sellami H, Znazen A, Sellami A, Mnif H, Louati N, Zarrouk SB, Keskes L, Rebai T, Gdoura R, Hammami A: **Molecular detection of Chlamydia trachomatis and other sexually transmitted bacteria in semen of male partners of infertile couples in Tunisia: The effect on semen parameters and spermatozoa apoptosis markers**. *PLoS ONE* 2014, **9(7) (no pagination)**.

170. Sinan BI, Samah HK, Maad MS: **Study on asymptomatic Neisseria gonorrhoeae cases among infertile subjects in Tikrit city and its surroundings دراسة عن حالات أعراض النيسرية البنية بين الموضوعات العقم في مدينة تكريت والمناطق المحيطة بها**. *The Medical Journal of Tikrit University* 2008, **1**(141):169-202.

171. Sinan BIسب: **A study on Mycoplasma and Ureaplasma species and their association with gonorrhea in infertile females in Tikrit district دراسة عن نوع المفطورة والميورة وارتباطهم 98 مع مرض السيلان في الإناث يعانون من العقم في منطقة تكريت**. *The Medical Journal of Tikrit University* 2010, **1**(161):98-104.

172. Al-Owaish RA, Anwar S, Sharma P, Shah SF: **HIV/AIDS prevalence among male patients in Kuwait**. *Saudi medical journal* 2000, **21(9)**:852-859.

173. Al Yazachi M, Al Mufti AW, Al Deliamy F: **Epidemiology of sexually transmitted diseases in Baghdad city/Iraq**. *Journal of Community Medicine* 1994, **7**(1):13-21.

174. Bhutto AM, Shah AH, Ahuja DK, Solangi AH, Shah SA: **Pattern of sexually transmitted infections in males in interior Sindh: a 10-year-study**. *Journal of Ayub Medical College, Abbottabad : JAMC* 2011, **23(3)**:110-114.

175. El Beayni N, Hamad L, Nakad C, Keleshian S, Yazbek SN, Mahfouz R: **Molecular prevalence of eight different sexually transmitted infections in a lebanese major tertiary care center: Impact on public health**. *International Journal of Molecular Epidemiology and Genetics* 2021, **12(2)**:16-23.

176. Heikel J, Sekkat S, Bouqdir F, Rich H, Takourt B, Radouani F, Hda N, Ibrahimy S, Benslimane A: **The prevalence of sexually transmitted pathogens in patients presenting to a casablanca STD clinic**. *European Journal of Epidemiology* 1999, **15(8)**:711-715.

177. WHO: **The 2004 First National Second Generation HIV/AIDS/STI Sentinel Surveillance Survey**. 2005b.

178. Behzadi MA, Davarpanah MA, Namayandeh M, Pourabbas B, Allahyari S, Ziyaeyan M: **Molecular diagnosis of genital tract infections among hiv-positive women in iran**. *Iranian Journal of Microbiology* 2018, **10(4)**:233-241.

179. Hashemi-Shahri SM, Sharifi-Mood B, Kouhpayeh HR, Moazen J, Farrokhian M, Salehi M: **Sexually transmitted infections among hospitalized patients with human immunodeficiency virus infection and acquired immune deficiency syndrome (HIV/AIDS) in Zahedan, Southeastern Iran**. *International journal of high risk behaviors & addiction* 2016, **5**(3):e28028.

180. Aneed MK, Ali NAH: **التشخيص الجزيئي والتحري عن جين الضراوة rmp لبكتريا gonorrhoeae Neisseria المعزولة من المرضى العراقيين**. *Al-Nahrain Journal of Science* 2014, **17**(2):30-40.

181. Fageeh WM: **Sexually transmitted infections among patients with herpes simplex virus at King Abdulaziz University Hospital**. *BMC research notes* 2013, **6**:301.

182. Ghafoor RTA, Yassin HAL: **Study of some virulence factors and virulence genes produced by gram (+Ve) and gram (-Ve) cocci isolated from clinical samples in Al-Anbar Province/Iraq**. *European Journal of Molecular and Clinical Medicine* 2020, **7(9)**:535-547.

183. Ghalib AK: **Chlamydia trachomatis infection in antenatal and gynecological patients in Kirkuk city. عدوى الكلاميديا ​​الحثرية في المرضى قبل الولادة وأمراض النساء في مدينة كركوك**. *The Medical Journal of Tikrit University* 2013, **19**(1):1-9.

184. Gharsallah H, Frikha-Gargouri O, Sellami H, Besbes F, Znazen A, Hammami A: **Chlamydia trachomatis genovar distribution in clinical urogenital specimens from Tunisian patients: High prevalence of C. trachomatis genovar E and mixed infections**. *BMC Infectious Diseases* 2012, **12 (no pagination)**.

185. Khanani R, Memon AR, Shaikh RB, Ali G, Shaikh M, Sandila T, Hussain Z, Parveen N: **Beta-lactamase producing Neisseria gonorrhea strains in Karachi**. *J Pak Med Assoc* 1994, **44**(3):70-71.

186. Ranjha KM, Dar NR, Hussain K: **Antibiotic sensitivity patterns of Neisseria gonorrhoeae and prevalence of PPNG amongst local clinical isolates**. *J Pak Med Assoc* 2001, **51**(9):339-340.

187. Taha OM, Ali MH, Omer EE, Ahmed MA, Abbaro SA: **Study of STDs in patients attending venereal disease clinics in Khartoum, Sudan**. *Br J Vener Dis* 1979, **55**(5):313-315.

188. Abdelaziz ZA, Ibrahim ME, Bilal NE, Hamid ME: **Vaginal infections among pregnant women at Omdurman Maternity Hospital in Khartoum, Sudan**. *Journal of Infection in Developing Countries* 2014, **8(4)**:490-497.

189. Ahmed HJ, Ilardi I, Antognoli A, Leone F, Sebastiani A, Amiconi G: **An epidemic of Neisseria gonorrhoeae in a Somali orphanage**. *Int J STD AIDS* 1992, **3**(1):52-53.

190. Al-Khafajii KA, Alwash B, Al-Hassnawi HH: **Etiological Agents of Pruritus Vulvae in Prepupertal Girls, In Hilla City**. *Medical Journal of Babylon* 2011, **8**(4):651-658.

191. Filemban SM, Yasein YA, Abdalla MHH, Al-Hakeem R, Al-Tawfiq JA, Memish ZA: **Prevalence and behavioral risk factors for STIs/HIV among attendees of the Ministry of Health hospitals in Saudi Arabia**. *Journal of Infection in Developing Countries* 2015, **9(4)**:402-408.

192. Kaushik P, Malaviya AN, Rotimi VO: **Infective arthritis in adults - Experience at a teaching hospital in Kuwait**. *Rheumatology International* 1999, **19(1-2)**:1-5.

193. Rehan N, Bokhari A, Nizamani NM, Jackson D, Naqvi HR, Qayyum K, Mansoor S, Muzaffar R: **National study of reproductive tract infections among high risk groups of Lahore and Karachi**. *Journal of the College of Physicians and Surgeons Pakistan* 2009, **19(4)**:228-231.

194. Hancali A, Bellaji B, Jennane S, Soraa N, Bennani A, Ghargui L, Rhilani H, Alami K, Rhajaoui M: **Prevalence of chlamydia trachomatis and neisseria gonorrhoeae among MSM in Morocco**. *Sexually Transmitted Infections* 2019, **95(Supplement 1)**:A244.

195. Gouya MM, Nabai S: **Prevalence of some sexually transmitted infections in a family planning service**. *Razi Journal of Medical Sciences* 2007, **14**(54):143-150.

196. Hancali A, Bellaji B, Jennane S, Bennani A, Kettani A, Ouanaim C, Oukouchoud H, Oumzil H, Charof R: **Trend of STIs prevalence among women and men in Morocco between 1999 to 2011**. *Sexually Transmitted Infections* 2015, **2)**:A155.

197. Hasanabad MH, Bahador A, Mohammadzadeh M, Haghighi F: **P3.272 Prevalence of Chlamydia Trachomatis, Neisseria Gonorrhoeae and Ureaplasma Urealyticum in Pregnant Women of Sabzevar - Iran**. *Sexually Transmitted Infections* 2013, **89**(Suppl 1):A233.233-A234.

198. Mahmood MA, Saniotis A: **Use of syndromic management algorithm for sexually transmitted infections and reproductive tract infections management in community settings in Karachi**. *Journal of the Pakistan Medical Association* 2011, **61(5)**:453-457.

199. Algeria Ministry of Health: **Enquete Algerienne Sur La Sante De La Famille**. In*.*; 2002.

200. Burans JP, Fox E, Omar MA, Farah AH, Abbass S, Yusef S, Guled A, Mansour M, Abu-Elyazeed R, Woody JN: **HIV infection surveillance in Mogadishu, Somalia**. *East African medical journal* 1990, **67(7)**:466-472.

201. Osama M: **Rate of STI among selected MSM and transgenders in 5 cities of Pakistan**. *Sexually Transmitted Infections* 2017, **93(Supplement 2)**:A18.

202. Al Jaufy AY, Al Rabie AM, Gharama FA: **Prevalence and determinants of sexually transmitted diseases in women attending antenatal clinics and gynecology health services in Sana’a, Yemen**. *Med J Cairo Univ* 2007, **75**(1):217-225.

203. Alwazer I (ed.): **Sexually transmitted diseases and reproductive health of women in Sana'a, Yemen**. Cairo, Egypt: WHO - EMRO; 2004.

204. Bokaeian M, Eghbal Ghoreyshi M, Dabiri S: **Antibiotic resistance of Neisseria gonorrhoeae isolated from gonorrhoeae patients**. *Zahedan Journal of Research in Medical Sciences* 2010, **12**(2):18-23.

205. Ramia S, Kobeissi L, el Kak F, Shamra S, Kreidieh K, Zurayk H: **Reproductive tract infections (RTIs) among married non-pregnant women living in a low-income suburb of Beirut, Lebanon**. *Journal of Infection in Developing Countries* 2012, **6(9)**:680-683.

206. Rushwan H: **Etiologic factors in pelvic inflammatory disease in Sudanese women**. *Am J Obstet Gynecol* 1980, **138**(7 Pt 2):877-879.

207. Zargooshi J: **Characteristics of gonorrhoea in Kermanshah, Iran**. *Sexually Transmitted Infections* 2002, **78(6)**:460-461.

208. Jaballah N: **Male infertility in Tunisia: apropos of 373 cases. [French]**. *Andrologia* 1987, **19 Spec No**:242-246.

209. Ashraf B, Shaukat S, Zahoor M, Ahmad TJ, Hussain I: **Frequency of HIV seropositivity in patients with sexually transmitted infections presenting in the dermatology department of a tertiary care hospital**. *J Pak Assoc Derma* 2020, **30**(2):249-255.

210. Ghanaat J, Sadeghian A, Ghazvini K, Nassiri MR: **Prevalence and risk factors for hepatitis B virus infections among STD patients in northeast region of Iran**. *Medical Science Monitor* 2003, **9(2)**:CR91-CR94.

211. Maan MA, Hussain F, Iqbal J, Akhtar SJ: **Sexually transmitted infections in Pakistan**. *Annals of Saudi Medicine* 2011, **31(3)**:263-269.

212. Maatouk I, Assi M, Jaspal R: **How can we enhance sexual health outcomes in men who have sex with men in Lebanon?** *BMJ Sex Reprod Health* 2020, **47(2)**:152-153.

213. Razvi SK, Najeeb S, Nazar HS: **Pattern of sexually transmitted diseases in patients presenting at Ayub teaching hospital, Abbottabad**. *Journal of Ayub Medical College, Abbottabad : JAMC* 2014, **26(4)**:582-583.

214. Ghassabi F, Malekzadegan Y, Sedigh Ebrahim-Saraie H, Heidari H, Sabet M, Bagheri A, Bagheri N, Raeisi Shahraki H, Hasanabadi A, Motamedifar M: **Gonorrhea and syphilis co-infection and related risk factors in HIV patients from Shiraz, South of Iran**. *Caspian J Intern Med* 2018, **9**(4):397-402.

215. Al-Mahroos F, Al-Amer E: **Reported child sexual abuse in Bahrain: 2000-2009**. *Ann Saudi Med* 2011, **31**(4):376-382.

216. Singh S, Singh SK: **Sexually transmitted infections in children: a prospective cross-sectional hospital-based study**. *Journal of Pakistan Association of Dermatologists* 2018, **28**(4):452-457.

217. Almroth L, Elmusharaf S, El Hadi N, Obeid A, El Sheikh MAA, Elfadil SM, Bergstrom S: **Primary infertility after genital mutilation in girlhood in Sudan: A case-control study**. *Lancet* 2005, **366(9483)**:385-391.

218. Dhamraa w A: **Effects of Interleukin-2 (IL-2) and Interleukin-6 (IL-6)in Recurrent Spontaneous Abortion (RSA)**. *Iraqi Journal of Pharmaceutical Sciences* 2008, **17**(2):74-79.

219. Diab KM: **Gonococcal and chlamydial antibodies in Egyptian women with ectopic pregnancy**. *New Egyptian Journal of Medicine [The]* 1993, **8**(4):1006-1010.

220. Elmusharaf S, Elkhidir I, Hoffmann S, Almroth L: **A case-control study on the association between female genital mutilation and sexually transmitted infections in Sudan**. *BJOG* 2006, **113**(4):469-474.
